# Synthesis and antimicrobial activity of sulfonyl-imidazole linked fused isoxazolo[3,4-b][1,2,3]triazolo[4,5-d]pyridines:PEG-400 mediated one-pot reaction under ultrasonic irradiation

**DOI:** 10.3389/fchem.2026.1784084

**Published:** 2026-03-13

**Authors:** Karukuri Premalatha, Ravikumar Kapavarapu, Sridhar Kavela, Sirassu Narsimha

**Affiliations:** 1 Department of Chemistry, Chaitanya (Deemed to be University), Hyderabad, India; 2 Department of Pharmaceutical Chemistry and Phytochemistry, Nirmala College of Pharmacy, Mangalgiri, Andhra Pradesh, India; 3 Department of Biotechnology, Chaitanya (Deemed to be University), Hyderabad, India

**Keywords:** antibacterial agents, antibiofilm activity, isoxazolo-triazolopyridines, MRSA, TLR4 docking, ultrasonication, VRSA

## Abstract

**Introduction:**

The rapid emergence of methicillin-resistant and vancomycin-resistant *Staphylococcus aureus* (MRSA and VRSA) represents a major global health challenge, necessitating the development of new antibacterial scaffolds with improved efficacy and safety.

**Methods:**

In this study, a novel series of sulfonyl-imidazole-linked fused isoxazolo[3,4-b][1,2,3]triazolo[4,5-d]pyridine derivatives (6a–6o) was synthesized using a PEG-400-mediated, ultrasound-assisted one-pot Cu(I)-catalysed strategy under environmentally benign conditions. The synthesized compounds were evaluated for antibacterial activity against MSSA, MRSA, and VRSA strains, along with antibiofilm activity, hemolytic potential using mouse erythrocytes, and cytotoxicity in RAW 264.7, THP-1, and BoMac cells. Immunomodulatory effects were assessed through cytokine induction studies.

**Results:**

Several derivatives exhibited potent antibacterial activity, with compound 6k emerging as the most active candidate, displaying MIC values of 1.56–3.12 μg/mL and outperforming the reference drug dicloxacillin. Selected compounds also showed significant antibiofilm activity against resistant *S. aureus* strains. Hemolysis and cytotoxicity assays demonstrated minimal toxicity, indicating good biocompatibility. Immunomodulatory analysis revealed moderate cytokine induction, suggesting a controlled immune response.

**Discussion:**

Overall, compound 6k was identified as a promising lead with potent antibacterial, antibiofilm, and immunomodulatory properties, combined with a favourable safety profile, warranting further preclinical development against drug-resistant *S. aureus* infections.

## Introduction

Bacteria are microscopic organisms that exert a profound impact on human health. Their pathogenic potential is evident in a wide spectrum of infections, ranging from relatively common ailments such as pharyngitis and urinary tract infections to severe and life-threatening diseases including meningitis and pneumonia (Awadh and Ahmed, 2025; Doron and Gorbach, 2008). In addition to direct infections, bacteria can release potent toxins that damage host tissues and organs, leading to long-term health complications (Lucas et al., 2020). Furthermore, disruptions in the gut microbiome caused by pathogenic or opportunistic bacteria have been linked to digestive, metabolic, and neuropsychiatric disorders (Xiong et al., 2023).

Among bacterial pathogens, *Staphylococcus aureus* (*S. aureus*) is a Gram-positive bacterium frequently colonizing the skin and mucosal surfaces of humans and animals (Boucher and Corey, 2008). Its extensive arsenal of virulence factors enables adhesion, invasion, immune evasion, and biofilm formation, allowing it to cause diseases ranging from mild skin infections to severe systemic conditions such as endocarditis, osteomyelitis, and sepsis (Rayner et al., 2005). Due to its adaptability and pathogenic versatility, *S. aureus* is regarded as one of the most dangerous human pathogens. Pietrocola et al. (2022) highlighted that *S. aureus* represents a major threat in hospital environments because of its ability to colonize medical devices and abiotic surfaces, resulting in healthcare-associated infections. Surgical wounds, indwelling catheters, and ventilator systems are particularly susceptible to colonization, placing hospitalized patients at increased risk. Moreover, close interpersonal contact facilitates community transmission, further amplifying its clinical relevance (Song et al., 2011).

The initial success of antibiotic therapy against *S. aureus* has been severely undermined by the widespread and often indiscriminate use of antibiotics, leading to the emergence of resistant strains (Davies and Davies, 2010). According to the World Health Organization (WHO), antimicrobial resistance has become a critical global health challenge, significantly complicating the treatment and management of bacterial infections (Salam et al., 2023). Bacteria have evolved multiple defense mechanisms—including enzymatic degradation, target modification, and efflux pumps—rendering many antibiotics ineffective. As a consequence, treatment failures, prolonged hospital stays, and increased healthcare costs have become increasingly common. Alarmingly, antimicrobial-resistant infections are estimated to cause approximately 1.27 million deaths annually, with an even greater burden in terms of disability-adjusted life years (DALYs) (Murray et al., 2022). These realities underscore the urgent need for the development of new antibacterial agents with novel chemical scaffolds and improved mechanisms of action (Lee et al., 2007).

**FIGURE 1:**
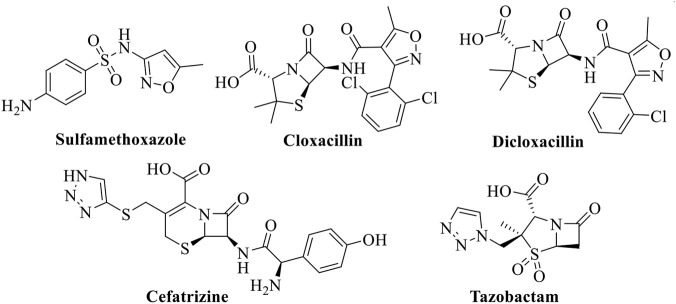
Examples are isoxazole and triazole-containing drugs in market.

**FIGURE 2:**
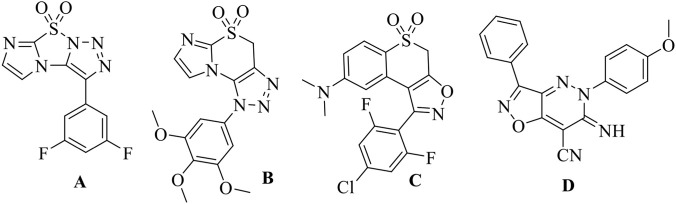
Antibacterially active fused triazoles and isoxazoles.

In recent years, heterocyclic compounds have emerged as promising candidates for addressing antimicrobial resistance. Heterocycles containing nitrogen and oxygen atoms constitute key structural motifs in numerous natural products and approved drugs (Dua et al., 2011). Among these, **1,2,3-triazoles** have received significant attention owing to their unique chemical stability, hydrogen-bonding capability, and broad spectrum of biological activities (Dheer et al., 2017; Rao and Srinivas, 2014; Ratcliffe et al., 2011; Suman et al., 2015). Triazole-based compounds have demonstrated potent antibacterial (Ashok et al., 2018), antifungal (Cao et al., 2017), antiprotozoal (Keivanloo et al., 2016), and antiviral activities (Niu et al., 2013). Importantly, the triazole nucleus is present in several clinically used drugs, such as tazobactam and cefatrizine (Figure 1), highlighting its pharmaceutical relevance. Recently, our research group reported sulfonyl-imidazole containing fused 1,2,3-triazoles (A–B) exhibiting promising antibacterial activity (Figure 2) (Premalatha et al., 2024; Ramu et al., 2023).

Similarly, isoxazoles represent another important class of heterocyclic compounds with wide-ranging biological and synthetic applications (Bacheley et al., 2024; Gaied and Zantour, 2000). Isoxazole derivatives frequently serve as key intermediates in the synthesis of active pharmaceutical ingredients and are present in marketed drugs (Figure 1) (Hu and Szostak, 2015). Several fused isoxazole-based compounds have been reported to exhibit potent antibacterial and antibiofilm activities. For example, compound C showed significant antibacterial and antibiofilm efficacy as reported by Nagu et al. (Deepavath et al., 2025), while compound D demonstrated notable antibacterial properties according to Abdel Reheim and co-workers (Abdel Reheim et al., 2025) (Figure 2). These findings suggest that hybridization of triazole and isoxazole pharmacophores could lead to enhanced antibacterial performance.

Alongside pharmacological considerations, the adoption of **green chemistry principles** has become increasingly important in modern synthetic methodologies. Green chemistry aims to minimize environmental impact while improving efficiency and safety in chemical processes (Kaur, 2018; Sucharitha et al., 2021; Swami et al., 2025). Non-classical synthetic techniques have gained prominence due to their ability to reduce hazardous by-products and eliminate toxic volatile organic solvents. Among these approaches, **ultrasound-assisted synthesis** has emerged as a powerful and environmentally friendly technique for accelerating organic transformations, often resulting in improved yields and reduced reaction times (Ahmed and Mohamed, 2024; Banerjee, 2017; Haseli et al., 2025; Jo et al., 2024; Johnpasha et al., 2024; Samala et al., 2023). In addition, **polyethylene glycols (PEGs)** have attracted considerable attention as green reaction media because of their low toxicity, thermal stability, recyclability, and biodegradability (Ballini et al., 2008; Jo et al., 2024; Johnpasha et al., 2024; Samala et al., 2023; Suman et al., 2007).

**SCHEME 1:**
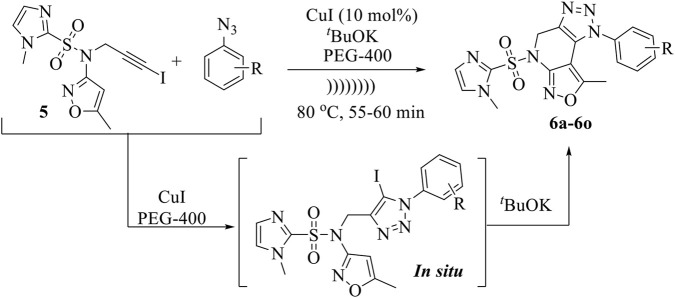
Synthesis of fused isoxazolo [3,4-b][1,2,3]triazolo [4,5-d]pyridines.

Guided by (i) the established biological importance of **isoxazole and 1,2,3-triazole scaffolds**, (ii) our continued interest in the development of biologically active fused heterocyclic systems (Bandgar et al., 2025; Chirra et al., 2024; Samala et al., 2022; Samala et al., 2024; Sucharitha et al., 2023; Udayasree et al., 2025), and (iii) the principles of sustainable synthesis using **ultrasonic irradiation and PEG-400**, we report herein the design and synthesis of a novel series of **imidazole-sulfonyl-1H-isoxazolo[3,4-b][1,2,3]triazolo[4,5-d]pyridine hybrids**. These compounds were synthesized via a **one-pot sequential 1,3-dipolar cycloaddition followed by intramolecular C–C bond coupling** using a low-cost Cu catalyst (Scheme 1).

Furthermore, the synthesized compounds were subjected to **comprehensive biological evaluation**, including antibacterial activity against MSSA, MRSA, and VRSA strains, antibiofilm efficacy, hemolytic toxicity, cytotoxicity on mammalian immune cells, and immunomodulatory potential. This integrated chemical–biological approach aims to identify lead molecules with **potent antibacterial activity, biofilm inhibition, and favorable safety profiles**, addressing the urgent need for new therapeutic agents against drug-resistant *S. aureus* infections.

## Results and discussion

**SCHEME 2:**
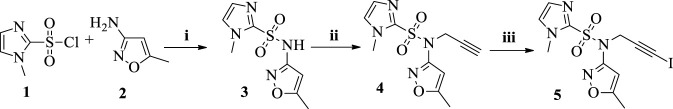
*Reagents & condition*. (i) K_2_CO_3_, Acetone, 60 °C, 6h. (ii) Propargyl bromide, Cs_2_CO_3_, DMF, rt, 6h. (iii) CuI, N-iodomorpholine, THF, rt, 2h.

As a starting point, 1-methyl-N-(5-methylisoxazol-3-yl)-1H-imidazole-2-sulfonamide (3) was produced in 78% yield by reacting 1-methyl-1H-imidazole-2-sulfonyl chloride (1) with 5-methylisoxazol-3-amine (2) in the presence of K_2_CO_3_ in Acetone for 6 h (Ramu et al., 2024). The intermediate alkyne 1-methyl-N-(5-methylisoxazol-3-yl)-N-(prop-2-yn-1-yl)-1H-imidazole-2-sulfonamide (4) was achieved in 73% yield when compound 3 reacted with propargyl bromide in the presence of Cs_2_CO_3_ in DMF at room temperature for 6 h (Narsimha et al., 2020). The corresponding key intermediate N-(3-iodoprop-2-yn-1-yl)-1-methyl-N-(5-methylisoxazol-3-yl)-1H-imidazole-2-sulfonamide (5) was synthesized through the CuI catalyzed reaction involving **4** and N-iodomorpholine in THF at room temperature over a duration of 2 h (Scheme 2) (Kumar et al., 2022).

Later, we broadened the synthetic application of a previously described Cu catalyzed [3 + 2]cycloaddition followed by C-C bond coupling reaction in one-pot technique (Jo et al., 2024; Johnpasha et al., 2024; Samala et al., 2023) for the synthesis of our desired fused isoxazolo [3,4-b][1,2,3]triazolo [4,5-d]pyridines (**6a-6o**) using iodo-alkyne (**5**), which reacts with various aryl azides using [3 + 2]cycloaddition reaction followed intramolecular C–C bond coupling reaction.

Several solvents, including DMF, DMSO, CH_3_CN, THF, and PEG-400, was used to compare the role and utility of green solvent systems. PEG-400 was identified as the most acceptable solvent system, which aids in the development of the product, 8-methyl-5-((1-methyl-1H-imidazol-2-yl)sulfonyl)-1-phenyl-4,5-dihydro-1H-isoxazolo [3,4-b][1,2,3]triazolo [4,5-d]pyridine (6a) under standard conditions at 70◦C–100 °C, 10 mol% CuI catalyst and 2 equivalent ^*t*^BuOK produced good yields of (6a). After running the procedure with several solvent conditions, a very low yield was obtained (entry 1–6, Table 1). Individual successes in PEG-400 for the synthesis of triazole derivatives inspired them to investigate the model reaction in their solvent system. The model reaction was tested at different temperatures and times in the PEG-400 solvent solution. The experimental results (entry 7–13, Table 1) revealed that the reaction was successfully carried out at 10/15 mol% CuI and 3 equivalents of ^*t*^BuOK in a PEG-400 solvent system while being irradiated with ultrasound waves. In addition to our interest, positive results have been observed in using sonication to create products with high yields in less reaction time; the optimal reaction condition was investigated in ultrasonication, and the temperature effect on the reaction was investigated (entry 7–10, Table 1). The results showed that ultrasonication at 80 °C resulted in a higher yield of product 6a in a shorter reaction time (60 min) than in traditional settings. Furthermore, the quantity of catalytic effect on the model reaction was explored by loading the catalyst in 15 and 20 mol% ratios (entry 11–12, Table 1), and it was discovered that the findings are not significantly different from those of 10 mol% CuI. And also increase base quantity in 3 equivalents of ^*t*^BuOK (entry 13, Table 1), and it was discovered that the findings are not significantly different from those of 2 equivalents of ^*t*^BuOK.

**TABLE 1:** Investigation of the reaction conditions for the synthesis of fused 1,2,3-triazole (6a)^a^.

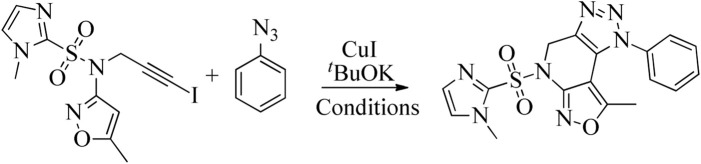					
Entry	Solvent	Condition	Temp (^o^C)	Time (h)	Yield (%)^b^
1	DMSO	Conventional	100	20	42
2	DMF	Conventional	100	20	49
3	THF	Conventional	70	24	36
4	CH_3_CN	Conventional	80	24	29
5	PEG-400	Conventional	100	20	58
6	PEG-400	Conventional	120	24	57
7	PEG-400	Ultrasonication	rt	60 min	61
8	PEG-400	Ultrasonication	60	50 min	71
9	PEG-400	Ultrasonication	80	60 min	83
10	PEG-400	Ultrasonication	100	50 min	78
11^c^	PEG-400	Ultrasonication	80	60 min	79
12^d^	PEG-400	Ultrasonication	80	50 min	81
13^e^	PEG-400	Ultrasonication	80	50 min	80

[a] = Reactions were carried out in solvent (8 mL) with 1.0 equiv. of **5**, 1.2 equiv of phenylazide, and 2 equiv of ^*t*^BuOK.

[b] = Yields are given for isolated products. [c] = 15 mol% of CuI. [d] = 20 mol% of CuI. [e] = 3 equiv of ^t^BuOK.

**SCHEME 3:**
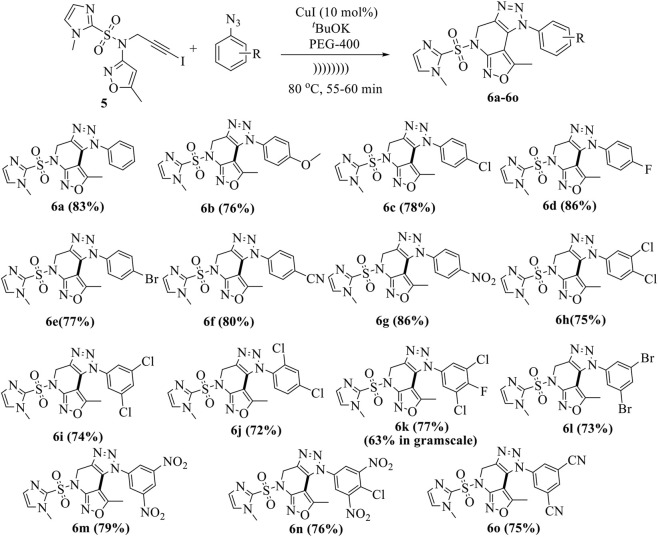
Synthesis of fused isoxazolo [3,4-b][1,2,3]triazolo [4,5-d]pyridines (6a-6o).

**SCHEME 4:**
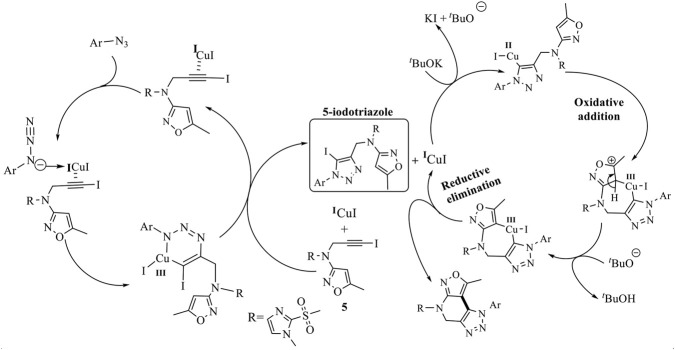
The proposed mechanism for the formation of fused isoxazolo [3,4-b][1,2,3]triazolo [4,5-*d*]pyridines.

Using the above optimum reaction conditions (i.e., iodo-alkyne 5 (1.0 mmol), arylazide (1.2 mmol), and CuI (10 mol%) and PEG-400 (8 mL), temperature 80 °C in US), the suggested methodology’s universality was then expanded to a variety of substituted azides to produce a broad range of fused isoxazolo [3,4-b][1,2,3]triazolo [4,5-d]pyridines in good to exceptional yields (Scheme 3). The proposed mechanism of the formation of fused derivatives is shown in Scheme 4.

### Minimum inhibitory concentration assay

**FIGURE 3:**
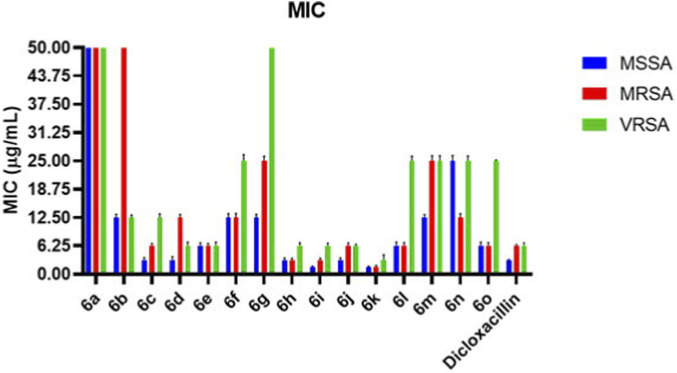
Comparative bar graph showing MIC values (µg/mL) of compounds 6a–6o against MSSA, MRSA, and VRSA. Data are expressed as mean values from triplicate experiments. Dicloxacillin was included as the reference standard. Several halogenated derivatives demonstrated activity comparable to or superior to that of the reference drug against resistant strains.

The antibacterial activity of compounds 6a–6o was evaluated against *Staphylococcus aureus* strains, including methicillin-susceptible (MSSA), methicillin-resistant (MRSA), and vancomycin-resistant (VRSA) isolates, using the broth microdilution method. MIC values are summarised in Table 2 and represent the mean of three independent experiments. The synthesised compounds exhibited MIC values of 1.56–50 μg/mL, demonstrating a strong dependence of antibacterial potency on aryl substitution. Several derivatives showed activity equal to or superior to that of dicloxacillin, particularly against resistant strains. Against MSSA, compounds 6i and 6k were the most potent, each with an MIC of 1.56 μg/mL, approximately 2-fold more active than dicloxacillin (MIC = 3.12 μg/mL). Compounds 6c, 6d, 6h, and 6j also showed strong inhibition (MIC = 3.12 μg/mL), whereas the unsubstituted analogue 6a and the methoxy-substituted compound 6b exhibited significantly weaker activity (MIC ≥12.5 μg/mL). A comparable trend was observed against MRSA. Compound 6k retained excellent potency (MIC = 1.56 μg/mL), followed by 6i (MIC = 3.12 μg/mL), both of which were significantly more active (≥4-fold) than dicloxacillin (MIC = 6.25 μg/mL). Mono-halogenated derivatives showed moderate activity, whereas nitro- and cyano-substituted compounds exhibited reduced efficacy. Notably, several compounds maintained activities against the highly resistant VRSA strain. Compound 6k emerged as the most potent VRSA inhibitor (MIC = 3.12 μg/mL), while 6h, 6i, and 6j showed consistent activity (MIC = 6.25 μg/mL), comparable to or better than dicloxacillin. In contrast, compounds bearing strongly polar substituents showed diminished or undetectable activity against VRSA (Figure 3). Overall, the MIC data indicate that halogen-rich aryl substitution, particularly dihalogenated and mixed-halogen patterns, significantly enhances antibacterial potency across all tested strains. The superior, statistically significant activity of compound 6k, observed across MSSA, MRSA, and VRSA in replicate assays, identifies it as the most promising antibacterial lead in this series.

**TABLE 2:** *In vitro* antibacterial activity data of compounds **6a-6o** ^[a]^.

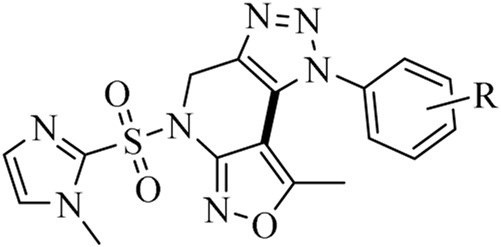				
​	​	Minimum inhibitory concentration (MIC)		
Compound	R	MSSA	MRSA	VRSA
6a	H	50 ± 1.31	50 ± 1.46	-
6b	4-OMe	12.5 ± 0.76	50 ± 1.14	12.5 ± 0.73
6c	4-Cl	3.12 ± 0.51	6.25 ± 0.52	12.5 ± 0.61
6d	4-F	3.12 ± 0.72	12.5 ± 0.73	6.25 ± 0.84
6e	4-Br	6.25 ± 0.63	6.25 ± 0.54	6.25 ± 0.81
6f	4-CN	12.5 ± 0.88	12.5 ± 0.92	25 ± 0.83
6g	4-NO_2_	12.5 ± 0.75	25 ± 1.05	-
6h	3,4-diCl	3.12 ± 0.43	3.12 ± 0.37	6.25 ± 0.60
6i	3,5-diCl	1.56 ± 0.21	3.12 ± 0.35	6.25 ± 0.63
6j	2,4-diCl	3.12 ± 0.46	6.25 ± 0.61	6.25 ± 0.57
6k	3,5-diCl,4-F	1.56 ± 0.16	1.56 ± 0.36	3.12 ± 0.32
6l	3,5-diBr	6.25 ± 0.82	6.25 ± 0.72	25 ± 1.13
6m	3,5-diNO_2_	12.5 ± 0.69	25 ± 1.16	25 ± 1.05
6n	3,5-diNO_2_, 4-Cl	25 ± 1.18	12.5 ± 0.88	25 ± 1.15
6o	3,5-diCN	6.25 ± 0.83	6.25 ± 0.69	25 ± 1.12
Dicloxacillin	**-**	3.12 ± 0.11	6.25 ± 0.27	6.25 ± 0.23

‘‘–’’Indicates concentration >50 μg/mL^[a]^MIC: i.e., the lowest concentration of the test compound to inhibit the growth of bacteria. Values are expressed as Mean ± SD.

### Antibiofilm activity

**FIGURE 4:**
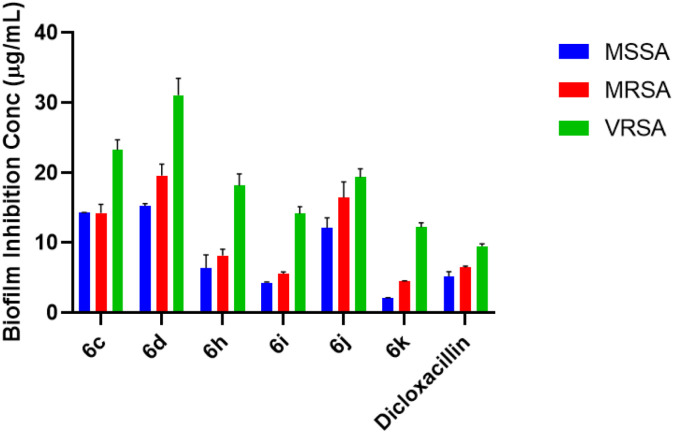
**Anti-biofilm activity of selected compounds against *Staphylococcus aureus*.** The bar graph represents the percentage inhibition of biofilm formation by compounds 6c, 6d, 6h, 6i, 6j, and 6k against MSSA, MRSA, and VRSA strains. Data are expressed as mean ± SD of triplicate experiments. Dicloxacillin was used as the reference control. Compounds 6k and 6i exhibited significantly enhanced anti-biofilm activity, including against VRSA.

The anti-biofilm potential of selected compounds was evaluated against MSSA, MRSA, and VRSA strains, as shown in Figure 4. All tested compounds demonstrated measurable inhibition of biofilm formation, with inhibition levels varying depending on strain resistance and substituent pattern. Among the tested derivatives, **compound 6k** displayed the **highest antibiofilm activity, demonstrating pronounced inhibition of** MSSA, MRSA, and VRSA. **Compound 6i** also exhibited strong and consistent biofilm suppression across all strains, followed by **compound 6h**, indicating that these derivatives effectively interfere with biofilm establishment even in resistant *S. aureus* phenotypes. In comparison, compounds **6c, 6d, and 6j** showed moderate biofilm inhibition, while dicloxacillin exhibited comparatively lower activity, particularly against MRSA and VRSA (Table 3). The observed trend correlates well with the MIC results, where **multi-halogenated derivatives (6k and 6i)** showed superior antibacterial potency. This suggests that enhanced hydrophobicity and optimal aryl substitution not only improve planktonic growth inhibition but also disrupt biofilm formation, potentially correlating with bacterial adhesion, membrane integrity, or quorum-sensing pathways. Mechanistic validation studies (e.g., membrane permeabilisation assays, gene expression studies, or quorum-sensing analyses) will be required in future work to confirm these hypotheses. Importantly, the ability of compounds 6k and 6i to inhibit biofilms formed by VRSA underscores their potential to overcome one of the most challenging resistance mechanisms in chronic *S. aureus* infections.

**TABLE 3:** Bacterial Film Inhibition Concentration (BFIC in *µ*g/mL) of compounds **6c**, **6d, 6h, 6i, 6j,** and **6k**.

Bacteria	*MSSA*	*MRSA*	*VRSA*
6c	14.34 ± 0.05**	14.33 ± 1.21*	23.33 ± 1.41
6d	15.32 ± 0.32*	19.65 ± 1.63	31.13 ± 2.39
6h	6.42 ± 1.85**	8.19 ± 0.89**	18.20 ± 1.69
6i	4.31 ± 0.12***	5.58 ± 0.26***	14.20 ± 1.01**
6j	12.13 ± 1.42*	16.53 ± 2.21*	19.43 ± 1.19
6k	2.12 ± 0.06***	4.50 ± 0.08***	12.23 ± 0.62*
Dicloxacillin	5.23 ± 0.65	6.47 ± 0.20	9.50 ± 0.34

Values are expressed as Mean ± Standard Deviation, ***P < 0.001, **P < 0.05.

### Hemolytic activity on mouse erythrocytes

**FIGURE 5:**
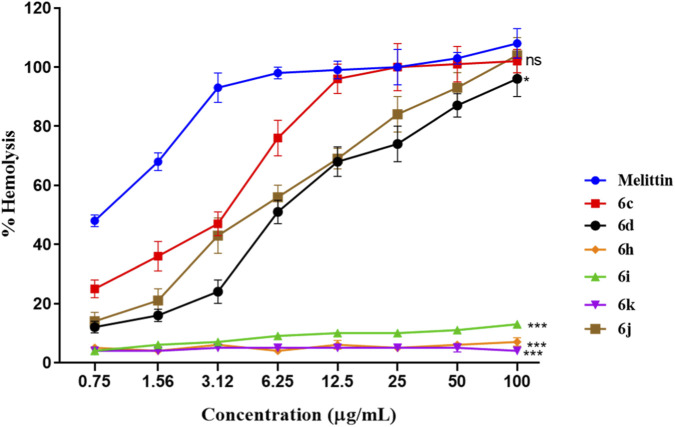
Hemolytic activity of selected compounds on mouse erythrocytes. Mouse red blood cells were incubated with increasing concentrations of compounds 6c, 6d, 6h, 6i, 6j, and 6k. Hemolysis (%) was quantified spectrophotometrically and compared with melittin as the positive control. Data are expressed as mean ± SD of three independent experiments. All tested compounds exhibited significantly lower hemolytic activity than melittin, indicating favorable hemocompatibility.

The hemolytic potential of selected compounds was evaluated using freshly isolated mouse red blood cells (RBCs) to assess membrane selectivity and hemocompatibility. Hemolysis was measured at increasing concentrations of the compound and compared with melittin, a well-established hemolytic peptide used as the positive control (Figure 5). All experiments were performed in triplicate, and data are expressed as mean ± SD. As expected, melittin induced rapid, concentration-dependent hemolysis, reaching near-complete erythrocyte lysis at higher concentrations, thereby confirming the assay’s sensitivity and validity. In contrast, all tested compounds exhibited significantly lower hemolytic activity across the entire concentration range (p < 0.05 vs. melittin). Among the evaluated derivatives, compound 6k showed a slight, dose-dependent increase in hemolysis at higher concentrations; however, the extent of erythrocyte lysis remained substantially lower than that induced by melittin at equivalent concentrations. Importantly, compounds 6i and 6 h demonstrated minimal hemolysis, even at the highest tested concentrations, indicating excellent erythrocyte compatibility. Other compounds, including 6c, 6d, and 6j, displayed negligible hemolytic effects, comparable to the negative control. From a biological perspective, hemolytic activity is a critical indicator of membrane selectivity and systemic safety for antimicrobial agents, particularly those designed to interact with lipid bilayers. While membrane-active peptides such as melittin exhibit strong antibacterial effects, their high hemolytic toxicity severely limits therapeutic applicability. In the present study, the markedly reduced hemolysis observed for all tested compounds—even at concentrations exceeding their antibacterial MIC values—suggests a preferential interaction with bacterial membranes over mammalian erythrocyte membranes. Notably, compound 6k, which displayed the most potent antibacterial and antibiofilm activity, maintained a favorablehemolytic profile, indicating a viable therapeutic window in which strong antibacterial efficacy is achieved without significant host cell damage. The consistently low hemolysis observed for 6i and 6 h further supports the conclusion that optimized halogen substitution and controlled hydrophobicity enhance antibacterial performance while minimizing nonspecific membrane disruption. Overall, the hemolysis data demonstrate that the synthesized compounds exhibit excellent hemocompatibility, reinforcing their suitability for further biological evaluation as antibacterial agents against drug-resistant *S. aureus*.

### Molecular docking

The binding interactions of the potential antimicrobial candidates were assessed through molecular docking studies performed using AutoDock Vina, implemented via the PyRx 0.8 virtual screening interface (Doron and Gorbach, 2008). Compounds 6c, 6d, 6h, 6i, 6j, and 6k, identified as potent inhibitors with notable MIC activity against MSSA, MRSA, and VRSA, were subjected to docking simulations to probe species-specific interactions with TLR4. To delineate differences in binding preferences, their affinities were evaluated against human (PDB ID: 3FXI) (Awadh and Ahmed, 2025), bovine (PDB ID: 3RG1) (Lucas et al., 2020), and mouse (PDB ID: 3VQ1) (Xiong et al., 2023) TLR4 structures.

In this docking study, the TLR4–MD-2 (Myeloid Differentiation Factor 2) binding pocket was investigated using the co-crystal structure with Eritoran (E55) (PDB ID: 2Z65) (Boucher and Corey, 2008). Eritoran, a synthetic lipid analogue of bacterial lipopolysaccharides developed by Eisai Pharmaceuticals, mimics the structural features of Lipid A and binds within the MD-2 cavity to prevent its recognition by the TLR4–MD-2 complex. By competitively blocking Lipid A, the endotoxin component essential for gram-negative bacterial pathogenicity and immune activation. Eritoran serves as a reference antagonist to map key residues involved in small-molecule interactions. Despite Eritoran’s clinical trial failure, its interaction profile provides critical structural insights that can inform the rational design of safer and more effective small-molecule modulators of TLR4.

**FIGURE 6:**
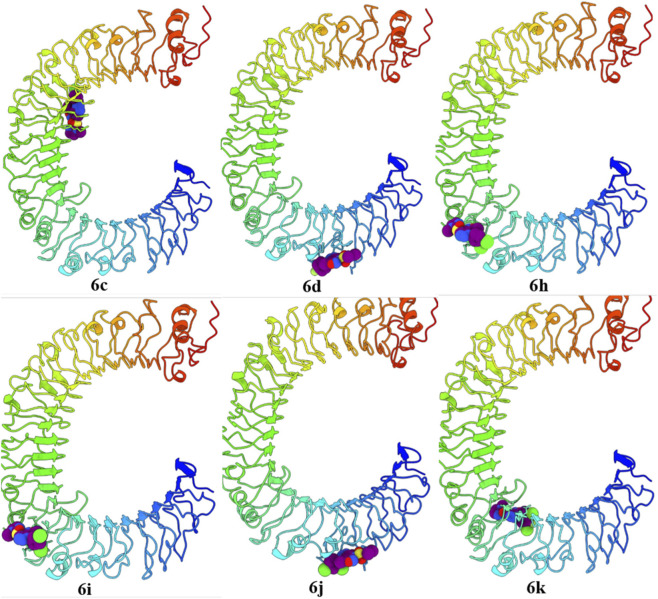
Binding sites and orientations of 6c, 6d, 6h, 6i, 6j and 6k compounds with human TLR4 proteins.

**Binding affinity profile of compounds with Human, Bovine and Mouse TLR4**: Docking simulations with human, mouse, and bovine TLR4 proteins showed that compound 6k had the highest binding affinity for human TLR4 (−8.3 kcal/mol), closely followed by compounds 6h, 6c, and 6i (−8.2 kcal/mol) (Table 4). Overall, the compounds exhibited marginally stronger interactions with human TLR4, suggesting a preference for this species and potential selective activity. Compound 6k displayed the highest binding affinity for human and bovine TLR4 variants, while compound 6 h showed superior affinity toward mouse TLR4. For most compounds, the binding sites were primarily located in the lower and curved region of human TLR4, whereas compound 6c bound at a distinct site near the top of the protein. This variation in binding site localization is illustrated in Figure 6.

**TABLE 4:** Binding affinity (kcal/mol) outcomes of compounds with human, bovine and mouse TLR4 from the docking assessments.

Compounds	Human TLR4	Bovine TLR4	Mouse TLR4
6c	−8.2	−7.4	−7.3
6d	−8.1	−7.3	−7.6
6h	−8.2	−7.3	−7.7
6i	−8.2	−7.1	−7.3
6j	−8.0	−7.3	−7.3
6k	−8.3	−7.5	−7.5
Dicloxacillin	−5.7	−5.6	−6.0

### Molecular interaction profile of compounds with human TLR4-MD2

Compounds 6i, 6c, 6h, and 6k, identified as high-affinity binders, possess a common structural feature, a chloro substitution on the phenyl ring linked to the triazolo–pyridine–oxazole scaffold. Among them, 6i and 6 h carry dichloro substitutions, 6k combines chloro and fluoro substituents, while 6c incorporates a single chloro group. Compound 6c exhibited a diverse set of non-covalent interactions stabilizing its binding within the active site. The oxazole, imidazole, and chloro-substituted phenyl ring engaged in π–σ interactions with VAL153, ILE153, and ILE63, respectively. The central triazole and pyridine rings formed a strong π–π stacking interaction with PHE151, reinforcing scaffold anchoring. A distinctive π–sulfur contact was observed between CYS133 and the tetrahydropyridine ring, complemented by additional π–alkyl interactions with the oxazole moiety. Furthermore, LEU61, ILE46, and VAL135 established π–alkyl interactions with both the halogenated phenyl ring and triazole units, while the chloro substituent specifically engaged in alkyl contacts with PHE76, PHE147, and ILE63 (Table 5).

**TABLE 5:** Molecular interaction summary of top compounds in E55 binding site of humanTLR4-MD2 protein.

Compounds	Binding energy (K.cal/mol)	Interacting amino acids	Nature of interactions
6c	−8.3	PHE151, ILE153, VAL135, CYS133, ILE63, LEU61, ILE46, PHE47, PHE76, VAL48, LEU149, ILE52, LEU78, LEU54, ILE124, PHE121, TYR131	π-sigma, π-sulfur, π-π stacked, π-alkyl, alkyl, van der waals
6d	−8.1	PHE151, CYS133, VAL135, ILE153, ILE63, LEU61, ILE46, PHE76, PHE147, LEU149, LEU78, TYR131, PHE121, LEU54, ILE52, VAL48	π-sigma, π-sulfur, π-π stacked, π-alkyl, van der waals
6h	−8.2	PHE151, PHE104, PHE76, ILE63, CYS133, VAL135, VAL113, LEU71, LEU74, LEU78, ILE46, PHE147, ILE117, THR115	π-sigma, π-π stacked, π-π T shaped, π-alkyl, alkyl, van der waals
6i	−8.4	TYR131, PHE151, PHE121, ILE124, VAL82, ILE80, CYS133, ILE153, ILE52, PHE119, VAL48, ILE32, ILE46, VAL24, LEU61, LEU54, LEU87	π-sigma, π-sulfur, π-π stacked, π-alkyl, alkyl, van der waals
6j	−8.1	PHE151, VAL135, CYS133, ILE153, TYR131, ILE124, ILE46, ILE63, LEU61, PHE147, PHE76, LEU78, ILE80, PHE121, VAL82, LEU54, ILE52, ILE32	π-sulfur, π-π stacked, π-alkyl, alkyl, van der waals
6k	−8.2	PHE151, ILE153, CYS133, LEU149, ILE46, PHE147, PHE76, LEU61, VAL135, ILE63, LEU78, TYR131, ILE124, PHE121, LEU54, ILE52, ILE32, VAL48	π-sigma, π-sulfur, π-π stacked, π-alkyl, alkyl, van der waals
E55	−7.2	**SER120,** ILE46, ILE63, TYR65, LEU87, TYR102, PHE104, ILE117, SER118, PHE119, PHE121, GLY123, CYS133, PHE151	H-bond, π-sigma, alkyl, π-alkyl, van der waals

Compound 6d retained the π–σ interaction profile of 6c, engaging VAL135, ILE63, and ILE153. The central triazole and aromatic scaffold engaged in π–π stacking with PHE151, while a notable π–sulfur interaction was retained with CYS133. In addition, LEU61 and VAL135 contributed π–alkyl interactions involving the triazole core and the fluoro-substituted phenyl ring, contributing to the binding stability.

Compound 6h exhibited a distinct hydrophobic interaction profile compared to 6c and 6d. The dichloro-substituted phenyl ring and central pyridine moiety mediated π–σ interactions with ILE63 and LEU61. The scaffold further engaged in π–π stacking and T-shaped interactions with PHE151, PHE76, and PHE104. Additionally, VAL135 and CYS133 formed π–alkyl contacts with the methyl-substituted oxazole and imidazole rings, while the dichloro substituents contributed to multiple alkyl interactions involving LEU74, LEU71, and VAL113. Compound 6i, a positional dichloro isomer of 6h, exhibited a markedly different interaction profile. The methyl-substituted imidazole ring engaged in π–π stacking with TYR131, while the sulphonyl group established a π–sulfur interaction with PHE121. Its binding was predominantly stabilized by numerous π–alkyl and alkyl contacts. The dichloro substituents contributed to extensive alkyl interactions with PHE151, VAL24, ILE46, ILE32, VAL48, PHE119, and ILE52. Furthermore, the aromatic heterocyclic rings of the scaffold formed multiple π–alkyl interactions with ILE124, VAL82, ILE80, CYS133, ILE153, ILE52, VAL48, and ILE32.

Compound 6j, a halogen positional isomer of 6h and 6i, combined overlapping and unique binding features. PHE151 was engaging in both π–sulfur bonding with the sulfonyl group, as in 6i, and π–π stacking with the methyl-substituted imidazole. Additional stabilization arose from π–alkyl interactions of the methyl-imidazole, methyl-oxazole, and dichloro-phenyl moieties with VAL135, CYS133, and ILE153. The methyl group on the imidazole and one chlorine atom of the dichloro substituent further reinforced binding through alkyl contacts with ILE46, ILE63, LEU61, ILE124, and TYR131. Compound 6k, bearing dichloro and fluoro substituents, displayed π–π stacking with PHE151 and a π–σ interaction with ILE153 through the methyl-imidazole ring of the central scaffold. Consistent with 6c and 6d, CYS133 contributed a π–sulfur interaction. Both chlorine atoms were engaged in hydrophobic alkyl contacts with LEU149, ILE46, PHE147, PHE76, and LEU61. Additional stabilization arose from π–alkyl interactions involving VAL135, LEU61, and CYS133.

**FIGURE 7:**
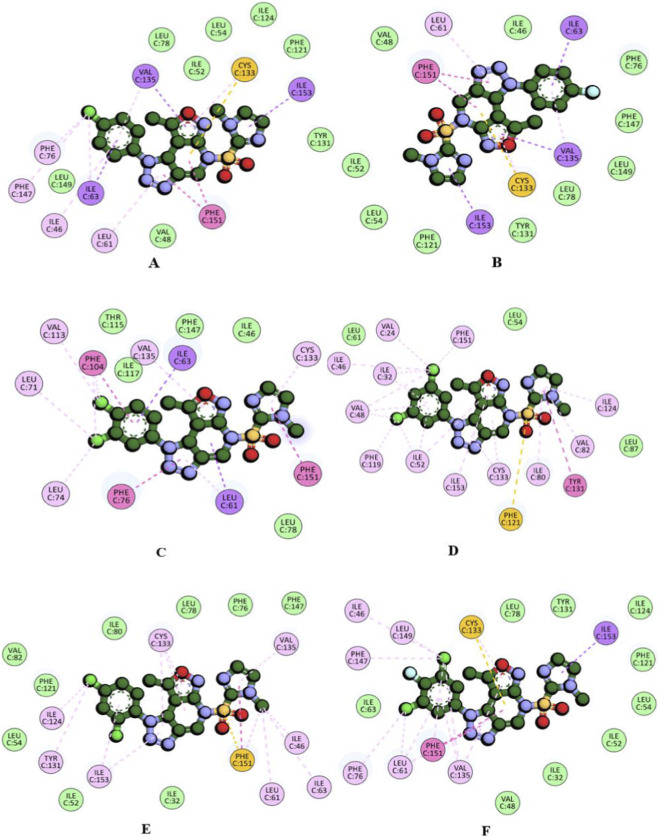
2D molecular interactions of **(A)** 6c, **(B)** 6d, **(C)** 6h, **(D)** 6i, **(E)** 6j, and **(F)** 6k compound with the active site residues of the TLR4 protein. Interactions were displayed as color coded dashed lines; green lines indicated the H–bonds.

Within the TLR4–MD2 complex, the co-crystallized ligand E55 is predominantly stabilized by hydrophobic contacts, consistent with the lipophilic character of the binding pocket and its extended aliphatic chains. A single polar interaction is observed, where the carbonyl group of one aliphatic chain forms a hydrogen bond with SER120. Similarly, all tested compounds relied mainly on hydrophobic interactions, reflecting the nonpolar nature of the pocket (Figure 5). The 2D interaction maps (Figure 7) illustrate these interactions, underscoring the extensive hydrophobic engagement of both E55 and the analogues within the TLR4–MD2 cavity. Molecular alignment of the evaluated compounds with E55 revealed similar binding modes within the MD2 active site pocket. Compounds 6i, 6c, 6h, and 6k occupied the binding pocket in a manner closely resembling the orientation of E55, as illustrated in Figures 5A,B. These compounds engaged in hydrophobic interactions with residues PHE151, CYS133, ILE153, VAL135, ILE63, LEU61, PHE76, ILE32, PHE147, ILE124, ILE46, and TYR131, as illustrated in Figure 4. Through its elongated aliphatic chains, E55 engaged in widespread hydrophobic alkyl interactions, reinforcing its binding. To further evaluate the stability of the docked, molecular dynamics (MD) simulations were performed for compound **6k** in complex with mouse TLR4 (PDB ID: 3VQI). The backbone RMSD profile of the protein–ligand complex remained within ∼0.2–0.45 nm throughout the 20 ns simulation, with an initial equilibration phase during the first ∼2 ns followed by stabilization around ∼0.28–0.35 nm. No large structural deviations or progressive drift were observed, indicating that the protein–ligand complex maintained overall structural stability during the simulation. A transient fluctuation reaching ∼0.48 nm was observed near 10 ns, which subsequently returned to the equilibrium range, suggesting local conformational adjustments rather than global instability. The absence of sustained RMSD escalation supports the structural compatibility of compound 6k within the TLR4 binding pocket (Supplementary Figure S1A). Root mean square fluctuation (RMSF) analysis revealed that most residues exhibited low fluctuations (<0.15 nm), indicating a stable protein backbone. Higher flexibility was observed in terminal regions and loop segments (notably residues ∼1–120 and ∼560–620), which is consistent with typical protein dynamics and not directly associated with the ligand-binding region. Importantly, residues within the predicted binding pocket showed relatively low fluctuation, suggesting stable ligand engagement during the simulation period (Supplementary Figure S1B). Overall, the MD simulation supports the stability of the docked 6k–TLR4 complex and reinforces the docking results by demonstrating that the ligand remains accommodated within the binding site without inducing major structural perturbations. These findings provide additional computational support for the proposed interaction between compound 6k and TLR4.

### Cytotoxicity evaluation on mammalian cells

**FIGURE 8:**
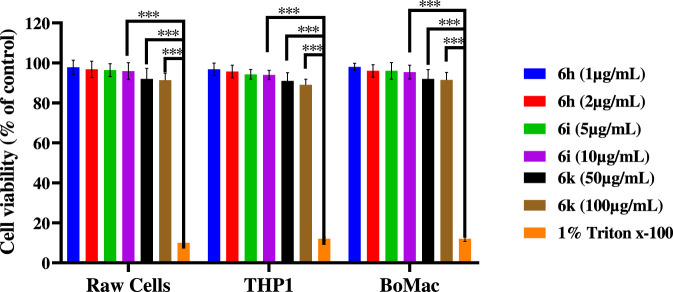
Cytotoxicity assessment of selected compounds on mammalian cells. RAW 264.7, THP-1, and BoMac cells were treated with compounds 6h, 6i, and 6k, and cell viability (%) was determined using a colorimetric assay. Data are presented as mean ± SD of triplicate experiments. Melittin was used as a positive cytotoxic control. All tested compounds exhibited minimal cytotoxicity and maintained high cell viability across all cell lines.

The cytotoxic effects of selected compounds were assessed in RAW 264.7 (mouse macrophages), THP-1 (human monocytic cells), and BoMac (bovine macrophage cells) to evaluate biocompatibility toward mammalian immune cells. Cell viability was determined using a colorimetric assay following compound exposure and expressed as a percentage relative to untreated controls (Figure 8). All experiments were performed in triplicate, and data are reported as mean ± SD. As shown in Figure 8, all tested compounds maintained high cell viability (>80–90%) across the three cell lines, indicating low cytotoxic potential. Compounds 6h, 6i, and 6k exhibited minimal cytotoxicity, with viability values statistically comparable to untreated controls (p > 0.01). No significant differences in viability were observed among RAW 264.7, THP-1, and BoMac cells, demonstrating consistent cellular tolerance across species and cell types. In contrast, the positive control 1% Triton X-100 induced a pronounced reduction in cell viability across all cell lines (p < 0.01 vs. untreated control), confirming the sensitivity and reliability of the assay. Vehicle-treated cells maintained near-complete viability, serving as an appropriate negative control. From a biological standpoint, cytocompatibility is a critical requirement for antimicrobial agents, particularly those designed to interact with bacterial membranes or modulate immune responses (Kavela et al., 2025). The absence of significant cytotoxic effects for the tested compounds—even at concentrations exceeding their antibacterial MIC values—indicates a favorable selectivity toward bacterial cells over mammalian immune cells. Importantly, compounds 6k and 6i, which displayed the strongest antibacterial and antibiofilm activities, showed no measurable cytotoxicity, suggesting a favorable therapeutic window and a high selectivity index. This observation supports the hypothesis that optimized halogen substitution and balanced hydrophobicity enhance antibacterial efficacy without causing nonspecific host cell damage. The consistently low cytotoxicity observed across murine, human, and bovine macrophage-derived cells further suggests the absence of species-specific toxicity. Overall, the cytotoxicity data demonstrate that the synthesized compounds possess excellent cytocompatibility, reinforcing their potential for further development as antibacterial agents, particularly for applications involving immune cells or biofilm-associated infections.

### Modulation of cytokine production in LPS-stimulated immune cells

**FIGURE 9:**
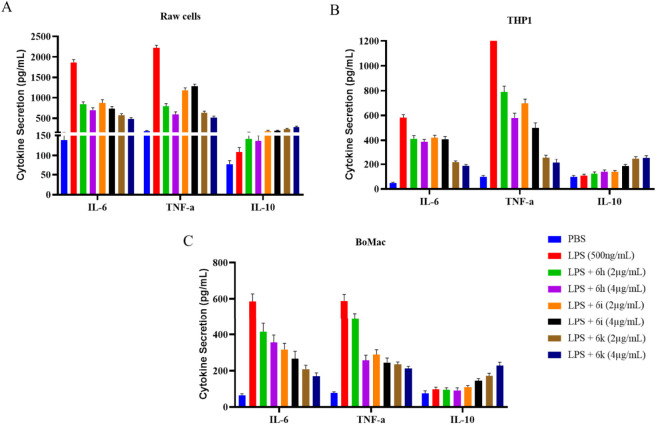
Anti-inflammatory activity of selected compounds in LPS-stimulated immune cells. Effect of selected compounds on lipopolysaccharide (LPS)–induced pro-inflammatory cytokine production in **(A)** RAW 264.7 (mouse macrophages), **(B)** THP-1 (human monocytic cells), and **(C)** BoMac (bovine macrophage cells). Cells were stimulated with LPS to induce an inflammatory response and subsequently treated with the test compounds. Cytokine levels were quantified using an immunoassay and expressed as mean ± SD of three independent experiments. LPS-treated cells served as the inflammatory control, while untreated cells were used as the basal control. Compounds 6k and 6i significantly reduced LPS-induced cytokine levels compared to the LPS control (*p* < 0.05–0.01), indicating effective anti-inflammatory activity without complete suppression of basal cytokine production.

The immunomodulatory potential of selected compounds was evaluated by examining their effects on lipopolysaccharide (LPS)–induced cytokine production in RAW 264.7, THP-1, and BoMac cells (Figure 9). Cells were stimulated with LPS to induce an inflammatory response, followed by treatment with the test compounds. The levels of key cytokines, including IL-6 and TNF-α (pro-inflammatory) and IL-10 (regulatory cytokine), were quantified and expressed as mean ± SD from three independent experiments. In RAW 264.7 cells, LPS stimulation produced a marked increase in IL-6 and TNF-α levels compared with unstimulated controls, confirming effective activation of inflammatory signaling. Treatment with the synthesized compounds resulted in a reduction in LPS-induced cytokine levels, with compound 6k showing the most pronounced effect, followed by 6i and 6 h. Importantly, although cytokine levels in compound-treated groups were significantly lower than in the LPS control (p < 0.01), they generally remained above basal (unstimulated) control levels, indicating modulation rather than complete suppression of cytokine production (Figure 9A). A comparable trend was observed in THP-1 cells (Figure 9B). Compounds 6k and 6i reduced LPS-induced IL-6 and TNF-α levels relative to LPS-treated controls (p < 0.01), while compound 6 h produced a moderate but consistent reduction. In BoMac cells, LPS stimulation again increased cytokine production, and treatment with the compounds led to a measurable decrease in these levels, with 6k showing the strongest modulatory effect. The overall cytokine response pattern in BoMac cells was like that observed in murine and human cell models, suggesting consistent cytokine modulation across species (Figure 9C). Across all three cell lines, the compounds reduced LPS-induced cytokine production relative to LPS-treated controls (p < 0.01) while maintaining cytokine levels above basal unstimulated conditions. These findings support the interpretation that the compounds modulate LPS-induced cytokine responses rather than causing broad immunosuppression. The observed reduction in LPS-induced cytokine levels suggests that the compounds may help moderate exaggerated inflammatory responses while preserving basal cytokine signaling. Notably, compounds 6k and 6i, which demonstrated strong antibacterial and antibiofilm activity, also showed consistent cytokine-modulating effects *in vitro*. However, further mechanistic studies will be required to determine the precise pathways involved in this modulation. Overall, the data indicate that selected compounds—particularly 6k and 6i—can attenuate LPS-induced cytokine elevation in immune cells while maintaining basal cytokine production. These findings support their potential as antibacterial agents with additional cytokine-modulating properties, warranting further mechanistic and *in vivo* investigation.

## Material and methods

### Synthesis of 1-methyl-N-(5-methylisoxazol-3-yl)-1H-imidazole-2-sulfonamide (3)

1-methyl-1H-imidazole-2-sulfonyl chloride (1) (3.6g, 0.02 mol) was introduced to a mixture of 5-methylisoxazol-3-amine (2) (1.96g, 0.02 mol) and K_2_CO_3_ (0.04 mol) in Acetone (40 mL) at 60 °C. The resulting solution was then agitated for duration of 7 h. The reaction mixture was concentrated under vacuum to yield a crude product, as confirmed by TLC analysis. After adding 50 mL of cold water to the unrefined substance, it was agitated for 15 min. The precipitate was collected and then the crude product was refined using silica gel chromatography. The eluent used for purification was a mixture of 10% ethyl acetate in hexane. White solid (Yield 78%). ^1^H-NMR (400 MHz, DMSO-d_6_) δ 10.13 (s, 1H, -NH), 7.83 (d, *J* = 8.0 Hz, 1H), 7.19 (d, *J* = 8.0 Hz, 1H), 6.69 (s, 1H), 3.61 (s, 3H, N-CH_3_), 2.18 (s, 3H, -CH_3_); ESI-MS: 243 [M + H]^+^.

### Synthesis of 1-methyl-N-(5-methylisoxazol-3-yl)-N-(prop-2-yn-1-yl)-1H-imidazole-2-sulfonamide (4)

A solution of 1-methyl-N-(5-methylisoxazol-3-yl)-1H-imidazole-2-sulfonamide (3) (2.5g, 0.01 mol), Propargyl bromide (0.013 mol) in DMF (30 mL), and Cs_2_CO_3_ (0.02 mol) was prepared. The reaction mixture was then agitated at room temperature for 3 h. The reaction process was assessed using thin-layer chromatography (TLC). Following this analysis, 50 mL of cold water was added to the crude product, and the mixture was agitated for 15 min. Following the collection of the solid particles, the impure substance was refined using the process of silica gel chromatography, using a solvent mixture of 10% ethyl acetate in hexane. The organic layers, when mixed, were cleansed with brine, dehydrated with anhydrous Na_2_SO_4_, and ultimately condensed under reduced pressure to provide compound **4**. White solid (Yield 73%). ^1^H-NMR (400 MHz, DMSO-d_6_) δ 7.82 (d, *J* = 8.0 Hz, 1H), 7.17 (d, *J* = 8.0 Hz, 1H), 6.67 (s, 1H), 4.21 (d, *J* = 4.0 Hz, 2H, N-CH_2_), 3.60 (s, 3H, N-CH_3_), 3.10 (t, *J* = 4.0Hz, 1H), 2.16 (s, 3H, -CH_3_); ESI-MS: 281 [M + H]^+^.

### Synthesis of N-(3-iodoprop-2-yn-1-yl)-1-methyl-N-(5-methylisoxazol-3-yl)-1H-imidazole-2-sulfonamide (5)

To 1-methyl-N-(5-methylisoxazol-3-yl)-N-(prop-2-yn-1-yl)-1H-imidazole-2-sulfonamide (4) (2g, 0.007 mol) was combined with THF (20 mL), CuI (2 mmol), and N-iodomorpholine (0.008 mol). The resulting reaction mixture was stirred at room temperature for 3 h, yielding a fine white precipitate. The suspension was introduced onto a pad of activated neutral alumina (30 mL), and the resulting filtrate was subsequently gathered. The solid phase underwent washing with CH_2_Cl_2_ (3 × 30 mL), and the resultant organic fractions were concentrated through evaporation under reduced pressure, yielding compound **5**. Yield (67%); ^1^H-NMR (400 MHz, DMSO-d_6_) δ 7.82 (d, *J* = 8.0 Hz, 1H), 7.15 (d, *J* = 8.0 Hz, 1H), 6.65 (s, 1H), 4.22 (s, 2H, N-CH_2_), 3.60 (s, 3H, N-CH_3_), 2.18 (s, 3H, -CH_3_); ESI-MS: 407 [M + H]^+^.

### General procedure for the synthesis of imidazole-sulfonyl-1H-isoxazolo [3,4-b][1,2,3]triazolo [4,5-d]pyridine (6a-6o)

A solution of alkyne (4) (0.001 mol) and aryl azide (5) (0.0012 mol) in PEG-400 (10 mL) was taken in a 50 mL round-bottom flask, followed by the addition of CuI (10 mol%). The reaction mixture was subjected to ultrasound irradiation under stirring at 80 °C for approximately 60 min, and the reaction progress was monitored by TLC. Upon completion, the mixture was extracted with diethyl ether, as PEG-400 is insoluble in ether. The ether layer was separated, dried over anhydrous sodium sulfate, and concentrated under reduced pressure. The crude product was purified by column chromatography using EtOAc/hexane (2:8) as the eluent. After extraction with dry ether, PEG-400 was recovered and reused several times without any noticeable loss in catalytic efficiency. This protocol afforded the desired compounds 6a–6o in good to excellent yields.

**8-methyl-5-((1-methyl-1H-imidazol-2-yl)sulfonyl)-1-phenyl-4,5-dihydro-1H-isoxazolo[3,4-b][1,2,3]triazolo[4,5-d]pyridine (6a):** Dirty white solid. M. p: 133–135 °C; ^1^H-NMR (400 MHz, DMSO-d_*6*_) δ 7.84 (d, *J* = 8.0 Hz, 1H), 7.64 (d, J = 8.0Hz, 2H), 7.33–7.28 (m, 3H), 7.16 (d, *J* = 8.0 Hz, 1H), 5.11 (s, 2H, N-CH_2_), 3.62 (s, 3H, N-CH_3_), 2.18 (s, 3H, -CH_3_); ^13^C-NMR (100 MHz, DMSO-d_*6*_): δ 171.24, 161.38, 158.67, 137.83, 129.40 (2C), 128.83, 126.37, 125.41, 124.29 (2C), 122.42, 121.26, 113.26, 42.31, 36.25, 16.63; ESI-MS: 398 [M + H]^+^. Anal. Calcd for C_17_H_15_N_7_O_3_S: C, 51.38; H, 3.80; N, 24.67. Found: C, 51.35; H, 3.77; N, 24.69.

**1-(4-methoxyphenyl)-8-methyl-5-((1-methyl-1H-imidazol-2-yl)sulfonyl)-4,5-dihydro-1H-isoxazolo[3,4-b][1,2,3]triazolo[4,5-d]pyridine (6b):** White solid. M. p: 158–160 °C; ^1^H-NMR (400 MHz, DMSO-d_6_) δ7.83 (d, *J* = 8.0 Hz, 1H), 7.70 (d, J = 8.0Hz, 2H), 7.16 (d, *J* = 8.0 Hz, 1H), 7.00 (d, J = 8.0Hz, 2H), 5.10 (s, 2H, N-CH_2_), 3.84 (s, 3H, OCH_3_), 3.62 (s, 3H, N-CH_3_), 2.18 (s, 3H, -CH_3_); ^13^C-NMR (100 MHz, DMSO-d_6_) δ 171.38, 161.33, 159.68, 158.34, 131.43, 127.04 (2C), 126.58, 125.95, 122.54, 121.18, 114.41 (2C), 113.26, 56.15, 42.57, 36.35, 16.46; ESI-MS: 428 [M + H]^+^. Anal. Calcd for C_18_H_17_N_7_O_4_S: C, 50.58; H, 4.01; N, 22.94. Found: C, 50.55; H, 3.98; N, 22.96.

**1-(4-chlorophenyl)-8-methyl-5-((1-methyl-1H-imidazol-2-yl)sulfonyl)-4,5-dihydro-1H-isoxazolo[3,4-b][1,2,3]triazolo[4,5-d]pyridine (6c):** Yellow solid. M. p: 145–147 °C; ^1^H-NMR (400 MHz, DMSO-d_6_) δ7.85 (d, *J* = 8.0 Hz, 1H), 7.73 (d, J = 8.0Hz, 2H), 7.39 (d, J = 8.0Hz, 2H), 7.17 (d, *J* = 8.0 Hz, 1H), 5.12 (s, 2H, N-CH_2_), 3.63 (s, 3H, N-CH_3_), 2.19 (s, 3H, -CH_3_); ^13^C-NMR (100 MHz, DMSO-d_6_): δ 171.30, 161.38, 158.36, 136.43, 133.39, 128.73 (2C), 126.85, 125.36 (2C), 124.32, 122.46, 121.31, 113.75, 42.68, 36.29, 16.79; ESI-MS: 432 [M + H]^+^. Anal. Calcd for C_17_H_14_ClN_7_O_3_S: C, 47.28; H, 3.27; N, 22.70. Found: C, 47.25; H, 3.25; N, 22.73.

**1-(4-fluorophenyl)-8-methyl-5-((1-methyl-1H-imidazol-2-yl)sulfonyl)-4,5-dihydro-1H-isoxazolo[3,4-b][1,2,3]triazolo[4,5-d]pyridine (6d):** Red solid. M. p: 139–141 °C; ^1^H-NMR (400 MHz, DMSO-d_6_) δ8.22 (d, J = 8.0Hz, 2H), 7.98 (d, J = 8.0Hz, 2H), 7.85 (d, *J* = 8.0 Hz, 1H), 7.16 (d, *J* = 8.0 Hz, 1H), 5.12 (s, 2H, N-CH_2_), 3.61 (s, 3H, N-CH_3_), 2.20 (s, 3H, -CH_3_); ^13^C-NMR (100 MHz, DMSO-d_6_): δ 171.24, 162.49, 161.24, 160.37, 158.36, 133.57, 126.81, 126.75, 125.41, 124.93, 122.59, 121.61, 116.73, 116.57, 113.26, 42.31, 36.33, 16.84; ESI-MS: 416 [M + H]^+^. Anal. Calcd for C_17_H_14_FN_7_O_3_S: C, 49.15; H, 3.40; N, 23.60. Found: C, 49.13; H, 3.38; N, 23.62.

**1-(4-bromophenyl)-8-methyl-5-((1-methyl-1H-imidazol-2-yl)sulfonyl)-4,5-dihydro-1H-isoxazolo[3,4-b][1,2,3]triazolo[4,5-d]pyridine (6e):** White solid. M. p: 153–155 °C; ^1^H-NMR (400 MHz, DMSO-d_6_) δ7.84 (d, *J* = 8.0 Hz, 1H), 7.64 (d, J = 8.0Hz, 2H), 7.49 (d, J = 8.0Hz, 2H), 7.16 (d, *J* = 8.0 Hz, 1H), 5.12 (s, 2H, N-CH_2_), 3.61 (s, 3H, N-CH_3_), 2.18 (s, 3H, -CH_3_); ^13^C-NMR (100 MHz, DMSO-d_6_): δ 171.56, 161.57, 158.47, 135.79, 132.31 (2C), 126.77, 125.49, 124.22 (2C), 122.23, 121.34, 120.66, 113.82, 42.57, 36.33, 16.81; ESI-MS: 476 [M + H]^+^. Anal. Calcd for C_17_H_14_BrN_7_O_3_S: C, 42.87; H, 2.96; N, 20.58. Found: C, 42.85; H, 2.94; N, 20.60.

**4-(8-methyl-5-((1-methyl-1H-imidazol-2-yl)sulfonyl)-4,5-dihydro-1H-isoxazolo[3,4-b][1,2,3]triazolo[4,5-d]pyridin-1-yl)benzonitrile (6f):** Pale red solid. M. p: 141–143 °C; ^1^H-NMR (400 MHz, DMSO-d_6_) δ7.92 (d, J = 8.0Hz, 2H), 7.83 (d, *J* = 8.0 Hz, 1H), 7.51 (d, *J* = 8.0 Hz, 2H), 7.17 (d, *J* = 8.0 Hz, 1H), 5.11 (s, 2H, N-CH_2_), 3.62 (s, 3H, N-CH_3_), 2.19 (s, 3H, -CH_3_); ^13^C-NMR (100 MHz, DMSO-d_6_): δ 171.37, 161.58, 158.40, 139.47, 127.71 (2C), 126.43 (2C), 125.68, 125.40, 122.77, 121.60, 119.59, 116.96, 113.31, 42.28, 36.64, 16.82; ESI-MS: 423 [M + H]^+^. Anal. Calcd for C_18_H_14_N_8_O_3_S: C, 51.18; H, 3.34; N, 26.53. Found: C, 51.16; H, 3.31; N, 26.55.

**8-methyl-5-((1-methyl-1H-imidazol-2-yl)sulfonyl)-1-(4-nitrophenyl)-4,5-dihydro-1H-isoxazolo[3,4-b][1,2,3]triazolo[4,5-d]pyridine (6g):** Yellow solid. M. p: 148–150 °C; ^1^H-NMR (400 MHz, DMSO-d_6_) δ8.34 (d, J = 8.0Hz, 2H), 8.14 (d, J = 8.0Hz, 2H), 7.85 (d, *J* = 8.0 Hz, 1H), 7.18 (d, *J* = 8.0 Hz, 1H), 5.12 (s, 2H, N-CH_2_), 3.63 (s, 3H, N-CH_3_), 2.20 (s, 3H, -CH_3_); ^13^C-NMR (100 MHz, DMSO-d_6_): δ 171.17, 161.62, 158.44, 148.71, 141.50, 127.73 (2C), 126.45, 125.44, 124.32 (2C), 122.38, 121.21, 113.33, 42.28, 36.65, 16.47; ESI-MS: 443 [M + H]^+^. Anal. Calcd for C_17_H_14_N_8_O_5_S: C, 46.15; H, 3.19; N, 25.33. Found: C, 46.13; H, 3.16; N, 25.37.

**1-(3,4-dichlorophenyl)-8-methyl-5-((1-methyl-1H-imidazol-2-yl)sulfonyl)-4,5-dihydro-1H-isoxazolo[3,4-b][1,2,3]triazolo[4,5-d]pyridine (6h):** Pale Yellow solid. M. p: 160–162 °C; ^1^H-NMR (400 MHz, DMSO-d_6_) δ7.85 (d, *J* = 8.0 Hz, 1H), 7.73 (s, 1H), 7.62 (d, J = 8.0Hz, 1H), 7.45 (d, J = 8.0Hz, 1H), 7.16 (d, *J* = 8.0 Hz, 1H), 5.11 (s, 2H, N-CH_2_), 3.61 (s, 3H, N-CH_3_), 2.18 (s, 3H, -CH_3_); ^13^C-NMR (100 MHz, DMSO-d_6_): δ 171.52, 161.34, 158.42, 138.06, 133.38, 131.61, 130.61, 126.73, 125.38, 124.80, 123.36, 122.48, 121.19, 113.30, 42.58, 36.61, 16.43; ESI-MS: 466 [M + H]^+^. Anal. Calcd for C_17_H_13_Cl_2_N_7_O_3_S: C, 43.79; H, 2.81; N, 21.03. Found: C, 43.76; H, 2.78; N, 21.05.

**1-(3,5-dichlorophenyl)-8-methyl-5-((1-methyl-1H-imidazol-2-yl)sulfonyl)-4,5-dihydro-1H-isoxazolo[3,4-b][1,2,3]triazolo[4,5-d]pyridine (6i):** Pale Yellow solid. M. p: 167–169 °C; ^1^H-NMR (400 MHz, DMSO-d_6_) δ7.85 (d, *J* = 8.0 Hz, 1H), 7.66 (s, 2H), 7.37 (s, 1H), 7.17 (d, *J* = 8.0 Hz, 1H), 5.12 (s, 2H, N-CH_2_), 3.62 (s, 3H, N-CH_3_), 2.18 (s, 3H, -CH_3_); ^13^C-NMR (100 MHz, DMSO-d_6_): δ 171.34, 161.15, 158.41, 139.51, 134.42 (2C), 126.69, 125.90, 124.84, 123.46 (2C), 122.40, 121.41, 113.28, 42.59, 36.29, 16.43; ESI-MS: 466 [M + H]^+^. Anal. Calcd for C_17_H_13_Cl_2_N_7_O_3_S: C, 43.79; H, 2.81; N, 21.03. Found: C, 43.76; H, 2.79; N, 21.05.

**1-(2,4-dichlorophenyl)-8-methyl-5-((1-methyl-1H-imidazol-2-yl)sulfonyl)-4,5-dihydro-1H-isoxazolo[3,4-b][1,2,3]triazolo[4,5-d]pyridine (6j):** Pale yellow solid. M. p: 164–166 °C; ^1^H-NMR (400 MHz, DMSO-d_6_) δ7.85 (d, *J* = 8.0 Hz, 1H), 7.70 (s, 1H), 7.62 (d, J = 8.0Hz, 1H), 7.43 (d, J = 8.0Hz, 1H),7.17 (d, *J* = 8.0 Hz, 1H), 5.12 (s, 2H, N-CH_2_), 3.62 (s, 3H, N-CH_3_), 2.19 (s, 3H, -CH_3_); ^13^C-NMR (100 MHz, DMSO-d_6_): δ 171.33, 161.40, 158.35, 136.45, 134.52, 133.52, 131.35, 130.33, 128.77, 126.35, 125.54, 121.82, 120.73, 113.28, 42.59, 36.32, 16.82; ESI-MS: 466 [M + H]^+^. Anal. Calcd for C_17_H_13_Cl_2_N_7_O_3_S: C, 43.79; H, 2.81; N, 21.03. Found: C, 43.75; H, 2.78; N, 21.06.

**1-(3,5-dichloro-4-fluorophenyl)-8-methyl-5-((1-methyl-1H-imidazol-2-yl)sulfonyl)-4,5-dihydro-1H-isoxazolo[3,4-b][1,2,3]triazolo[4,5-d]pyridine (6k):** Red solid. M. p: 173–175 °C; ^1^H-NMR (400 MHz, DMSO-d_6_) δ7.85 (d, *J* = 8.0 Hz, 1H), 7.72 (s, 2H), 7.17 (d, *J* = 8.0 Hz, 1H), 5.12 (s, 2H, N-CH_2_), 3.62 (s, 3H, N-CH_3_), 2.20 (s, 3H, -CH_3_); ^13^C-NMR (100 MHz, DMSO-d_6_): δ 171.42, 161.79, 158.46, 156.43, 154.32, 134.78, 126.74, 125.73, 125.28, 124.90, 124.31, 124.25, 122.21, 121.20, 113.35, 42.60, 36.36, 16.49; ESI-MS: 484 [M + H]^+^. Anal. Calcd for C_17_H_12_C_l2_FN_7_O_3_S: C, 42.16; H, 2.50; N, 20.25. Found: C, 42.15; H, 2.48; N, 20.27.

**1-(3,5-dibromophenyl)-8-methyl-5-((1-methyl-1H-imidazol-2-yl)sulfonyl)-4,5-dihydro-1H-isoxazolo[3,4-b][1,2,3]triazolo[4,5-d]pyridine (6L):** White solid. M. p: 167–169 °C; ^1^H-NMR (400 MHz, DMSO-d_6_) δ7.84 (d, *J* = 8.0 Hz, 1H), 7.57 (s, 2H), 7.39 (s, 1H), 7.17 (d, *J* = 8.0 Hz, 1H), 5.11 (s, 2H, N-CH_2_), 3.61 (s, 3H, N-CH_3_), 2.18 (s, 3H, -CH_3_); ^13^C-NMR (100 MHz, DMSO-d_6_): δ 171.50, 161.30, 158.40, 138.39, 130.64 (2C), 126.63, 125.98, 124.90 (2C), 123.16, 122.21, 121.20, 113.32, 42.47, 36.59, 16.48; ESI-MS: 556 [M+3H]^+^. Anal. Calcd for C_17_H_13_Br_2_N_7_O_3_S: C, 36.78; H, 2.36; N, 17.66. Found: C, 36.75; H, 2.34; N, 17.68.

**1-(3,5-dinitrophenyl)-8-methyl-5-((1-methyl-1H-imidazol-2-yl)sulfonyl)-4,5-dihydro-1H-isoxazolo[3,4-b][1,2,3]triazolo[4,5-d]pyridine (6m):** Yellow solid. M. p: 172–174 °C; ^1^H-NMR (400 MHz, DMSO-d_6_) δ8.33 (s, 2H), 8.11 (s, 1H), 7.87 (d, *J* = 8.0 Hz, 1H), 7.20 (d, *J* = 8.0 Hz, 1H), 5.13 (s, 2H, N-CH_2_), 3.63 (s, 3H, N-CH_3_), 2.20 (s, 3H, -CH_3_); ^13^C-NMR (100 MHz, DMSO-d_6_): δ 171.33, 161.68, 158.36, 149.63 (2C), 137.24, 126.85, 126.31, 125.45 (2C), 124.52, 122.72, 121.24, 113.35, 42.56, 36.63, 16.80; ESI-MS: 488 [M + H]^+^. Anal. Calcd for C_17_H_13_N_9_O_7_S: C, 41.89; H, 2.69; N, 25.86. Found: C, 41.84; H, 2.66; N, 25.88.

**1-(4-chloro-3,5-dinitrophenyl)-8-methyl-5-((1-methyl-1H-imidazol-2-yl)sulfonyl)-4,5-dihydro-1H-isoxazolo[3,4-b][1,2,3]triazolo[4,5-d]pyridine (6n):** Yellow solid. M. p: 178–180 °C; ^1^H-NMR (400 MHz, DMSO-d_6_) δ8.28 (s, 2H), 7.85 (d, *J* = 8.0 Hz, 1H), 7.19 (d, *J* = 8.0 Hz, 1H), 5.14 (s, 2H, N-CH_2_), 3.63 (s, 3H, N-CH_3_), 2.21 (s, 3H, -CH_3_); ^13^C-NMR (100 MHz, DMSO-d_6_): δ 171.28, 161.32, 158.30, 140.32 (2C), 137.10, 126.91, 126.39 (2C), 125.24, 123.61, 122.75, 121.25, 113.22, 42.58, 36.33, 16.43; ESI-MS: 522 [M + H]^+^. Anal. Calcd for C_17_H_12_ClN_9_O_7_S: C, 39.13; H, 2.32; N, 24.16. Found: C, 39.10; H, 2.30; N, 24.19.

**5-(8-methyl-5-((1-methyl-1H-imidazol-2-yl)sulfonyl)-4,5-dihydro-1H-isoxazolo[3,4-b][1,2,3]triazolo[4,5-d]pyridin-1-yl)isophthalonitrile (6o):** White solid. M. p: 161–163 °C; ^1^H-NMR (400 MHz, DMSO-d_6_) δ7.84 (d, *J* = 8.0 Hz, 1H), 7.77 (s, 2H), 7.47 (s, 1H), 7.18 (d, *J* = 8.0 Hz, 1H), 5.12 (s, 2H, N-CH_2_), 3.62 (s, 3H, N-CH_3_), 2.19 (s, 3H, -CH_3_); ^13^C-NMR (100 MHz, DMSO-d_6_): δ 171.66, 161.46, 158.30, 133.55, 128.44 (2C), 128.23, 126.40, 125.28, 122.73, 121.67, 118.62 (2C), 116.74 (2C), 113.23, 42.22, 36.54, 16.54; ESI-MS: 448 [M + H]^+^. Anal. Calcd for C_19_H_13_N_9_O_3_S: C, 51.00; H, 2.93; N, 28.17. Found: C, 50.98; H, 2.89; N, 28.21.

### Bacterial strains and culture conditions

Methicillin-susceptible *Staphylococcus aureus* (MSSA), methicillin-resistant *S. aureus* (MRSA), and vancomycin-resistant *S. aureus* (VRSA) strains were used for antibacterial and antibiofilm studies. Bacterial strains were cultured in Mueller–Hinton broth (MHB) at 37 °C under aerobic conditions. Fresh overnight cultures were diluted to the required inoculum density before each experiment.

### Determination of minimum inhibitory concentration (MIC)

The minimum inhibitory concentration (MIC) of the synthesized compounds (6a–6o) was determined using the broth microdilution method, following Clinical and Laboratory Standards Institute (CLSI) guidelines. Briefly, serial two-fold dilutions of the test compounds ranging from 0.78 to 100 μg/mL (All compounds wereinitially dissolved in 10% DMSO with in well conc less than 0.01%DMSO) were prepared in MHB in sterile 96-well microtiter plates. Bacterial suspensions were adjusted to approximately 1 × 10^6^ CFU/mL, and 100 µL of the suspension was added to each well containing 100 µL of the compound solution. Dicloxacillin was used as the reference antibiotic control, while wells containing broth and bacteria without compounds served as growth controls. Plates were incubated at 37 °C for 18–24 h, and bacterial growth was assessed visually and spectrophotometrically. The MIC was defined as the lowest concentration of compound that completely inhibited visible bacterial growth. All experiments were performed in triplicate.

### Anti-biofilm activity assay

The anti-biofilm activity of selected compounds was evaluated using a crystal violet–based microtiter plate assay as described (Kavela et al., 2025). Briefly, bacterial cultures were grown overnight and diluted in MHB supplemented with 1% glucose to a final concentration of 1 × 10^6^ CFU/mL. Aliquots (200 µL) were dispensed into sterile 96-well plates containing test compounds at the desired concentrations. Plates were incubated at 37 °C for 24 h to allow biofilm formation. Following incubation, non-adherent cells were removed by gently washing the wells with phosphate-buffered saline (PBS). The remaining biofilms were fixed with methanol, stained with 0.1% (w/v) crystal violet for 15 min, and excess stain was removed by washing with distilled water. The bound dye was solubilized using 95% ethanol, and absorbance was measured at 570 nm. Biofilm inhibition was calculated relative to untreated control wells. Dicloxacillin was included as a reference control. All assays were conducted in triplicate.

### Ethical approval for animal blood collection

All procedures involving animals were conducted in accordance with the guidelines of the Institutional Animal Ethics Committee (IAEC) and the Committee for Control and Supervision of Experiments on Animals (CCSEA), Government of India. Blood samples for the hemolysis assay were obtained from healthy mice following approval by the IAEC of Jeeva Life Sciences, Uppal, Hyderabad, India.

### Hemolytic activity assay

Hemolytic activity was evaluated using freshly isolated mouse red blood cells (RBCs) to assess hemocompatibility (Kakkerla et al., 2025). Whole blood was collected from healthy mice into EDTA-containing tubes and centrifuged at 1500 rpm for 10 min to separate RBCs. The cells were washed three times with PBS and resuspended to a final concentration of 2% (v/v) in PBS. Test compounds at various concentrations were incubated with the RBC suspension at 37 °C for 1 h. Melittin was used as a positive control, while PBS-treated RBCs served as the negative control. After incubation, samples were centrifuged, and the absorbance of the supernatant was measured at 540 nm to quantify released hemoglobin. Percentage hemolysis was calculated relative to the positive control. Experiments were performed in triplicate.

### Cell lines

The cell lines present in this study were obtained from the American Type Culture Collection (ATCC, Manassas, VA, United States of America).

### Cytotoxicity evaluation using MTT assay

The cytotoxic effects of selected compounds were assessed on RAW 264.7 TIB-71 (mouse macrophages), THP-1 TIB-202 (human monocytic cells), and BoMac (bovine macrophage cells) using the MTT assay. Cells were cultured in DMEM growth media supplemented with 10% fetal bovine serum and antibiotics and maintained at 37 °C in a humidified atmosphere containing 5% CO_2_. Cells were seeded into 96-well plates at a density of 1 × 10^4^ cells per well and allowed to adhere overnight. The cells were then treated with test compounds at the indicated concentrations and incubated for 24 h. 1% Triton X-100 was used as a positive cytotoxic control, and untreated cells served as negative controls. Following treatment, MTT solution (5 mg/mL) was added to each well and incubated for 4 h. The resulting formazan crystals were dissolved in dimethyl sulfoxide (DMSO), and absorbance was measured at 570 nm. Cell viability was expressed as a percentage relative to untreated control cells. All experiments were performed in triplicate.

### Immunomodulatory activity in LPS-stimulated cells

The anti-inflammatory activity of selected compounds was evaluated by measuring their ability to modulate lipopolysaccharide (LPS)–induced production of pro-inflammatory cytokines in RAW 264.7, THP-1, and BoMac cells. Cells were seeded in 96-well plates and pretreated with test compounds at non-cytotoxic concentrations. Inflammation was induced by stimulation with LPS (1 μg/mL) for the indicated time—LPS-treated cells without compounds served as inflammatory controls, while untreated cells were used as basal controls. Following incubation, culture supernatants were collected, and cytokine levels (IL-6, TNF-α and IL-10) were quantified using commercially available ELISA kits, according to the manufacturer’s instructions. Cytokine concentrations were calculated from standard curves and expressed as mean ± SD of three independent experiments.

### Molecular dynamics simulation

Molecular dynamics (MD) simulations were performed to assess the stability of the docked complex of compound 6k with mouse TLR4 (PDB ID: 3VQI). The protein–ligand complex obtained from docking was prepared and simulated using the GROMACS software package. The system was parameterised using the CHARMM force field, and ligand topology was generated using an appropriate parameterisation tool. The complex was solvated in a cubic box with TIP3P water molecules, and counter ions were added to neutralize the system. After energy minimization, the system was equilibrated under NVT and NPT conditions at 300 K and 1 atm. A 20 ns production MD simulation was then performed with a 2 fs time step under periodic boundary conditions. Structural stability of the complex was evaluated by calculating backbone root-mean-square deviation (RMSD) and residue-wise root-mean-square fluctuation (RMSF) throughout the simulation.

### Statistical analysis

All experiments were performed in triplicate, and data are presented as mean ± standard deviation (SD). Statistical significance was determined using appropriate statistical tests (e.g., one-way ANOVA followed by *post hoc* analysis), with p < 0.05 considered statistically significant.

## Data Availability

The original contributions presented in the study are included in the article/Supplementary Material, further inquiries can be directed to the corresponding authors.
