# Supplementary figures and images for: Synthesis and antimicrobial activity of sulfonyl-imidazole linked fused isoxazolo[3,4-b][1,2,3]triazolo[4,5-d]pyridines:PEG-400 mediated one-pot reaction under ultrasonic irradiation
Source: Front Chem. 2026 Mar 13;14:1784084. doi: 10.3389/fchem.2026.1784084 (PMC13035402; doi:10.3389/fchem.2026.1784084)

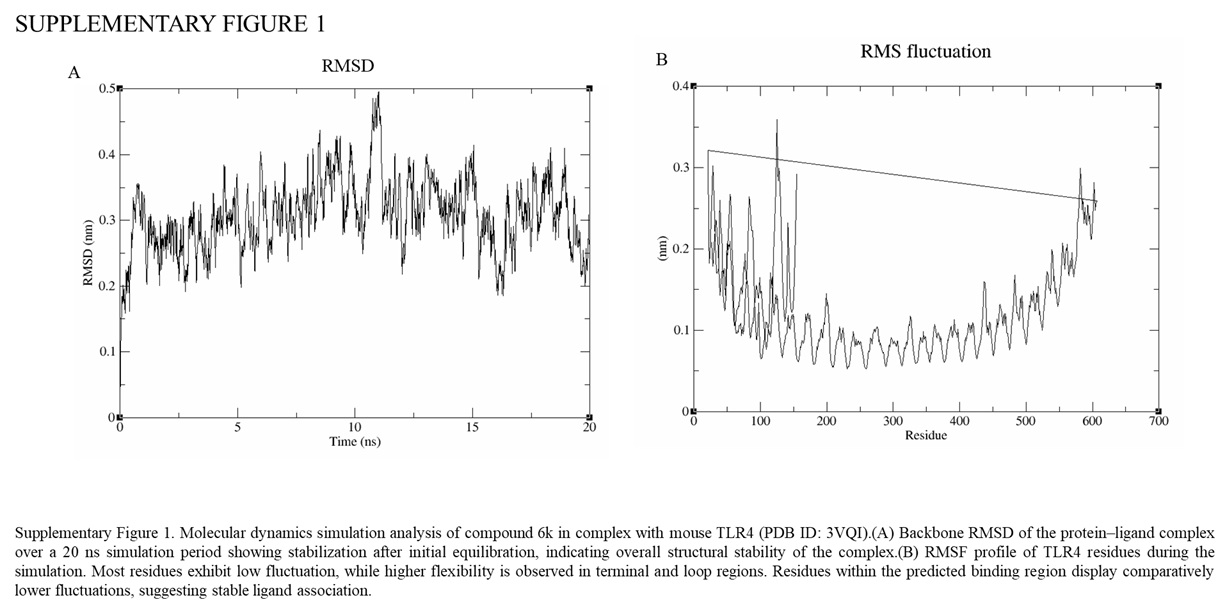

Supplement: Supplementary file 1 [file Image1.jpg]
