# Supplementary material for: Synthesis and antimicrobial activity of sulfonyl-imidazole linked fused isoxazolo[3,4-b][1,2,3]triazolo[4,5-d]pyridines:PEG-400 mediated one-pot reaction under ultrasonic irradiation
Source: Front Chem. 2026 Mar 13;14:1784084. doi: 10.3389/fchem.2026.1784084 (PMC13035402; doi:10.3389/fchem.2026.1784084)

**Supporting information**

**Synthesis and antimicrobial activity of Sulfonyl-imidazole linked fused isoxazolo[3,4-b][1,2,3]triazolo[4,5-d]pyridines:** **PEG-400 mediated one-pot reaction under ultrasonic irradiation**

Karukuri Premalatha^a^, Ravikumar Kapavarapu^c^, Sridhar Kavela^b*^, and Sirassu Narsimha*^a^

*^a^Department of Chemistry, Chaitanya (Deemed to be University), Hyderabad, India*

*^b^Department of Biotechnology, Chaitanya (Deemed to be University), Hyderabad, India*

*^c^Department of Pharmaceutical Chemistry and Phytochemistry, Nirmala College of Pharmacy, Atmakur, Mangalgiri, Andhra Pradesh, India*

*^*^Equal corresponding authors*

E-mail address: [narsimha.s88@chaitanya.edu.in](mailto:narsimha.s88@chaitanya.edu.in), & [sridharkavela@chaitanya.edu.in](mailto:sridharkavela@chaitanya.edu.in).

**Experimental**

**Synthesis of 1-methyl-N-(5-methylisoxazol-3-yl)-1H-imidazole-2-sulfonamide (3)**

1-methyl-1H-imidazole-2-sulfonyl chloride (1) (3.6g, 0.02 mol) was introduced to a mixture of 5-methylisoxazol-3-amine (2) (1.96g, 0.02 mol) and K_2_CO_3_ (0.04 mol) in Acetone (40 mL) at 60 ^o^C. The resulting solution was then agitated for duration of 7hours. The reaction mixture was subjected to vacuum concentration to yield a crude product, as confirmed by TLC analysis. Following the addition of 50 mL of cold water to the unrefined substance, it was agitated for duration of 15 minutes. The precipitate was collected and then the crude product was refined using silica gel chromatography. The eluent used for purification was a mixture of 10% ethyl acetate in hexane. White solid (Yield 78%). ^1^H-NMR (400 MHz, DMSO-d_6_) δ 10.13 (s, 1H, -NH), 7.83 (d, *J* = 8.0 Hz, 1H), 7.19 (d, *J* = 8.0 Hz, 1H), 6.69 (s, 1H), 3.61 (s, 3H, N-CH_3_), 2.18 (s, 3H, -CH_3_); ESI-MS: 243 [M+H]^+^.

**Synthesis of** **1-methyl-N-(5-methylisoxazol-3-yl)-N-(prop-2-yn-1-yl)-1H-imidazole-2-sulfonamide (4)**

A solution of 1-methyl-N-(5-methylisoxazol-3-yl)-1H-imidazole-2-sulfonamide (3) (2.5g, 0.01 mol), Propargyl bromide (0.013 mol) in DMF (30 mL), and Cs_2_CO_3_ (0.02 mol) was prepared. The reaction mixture was then agitated at room temperature for 3hours. The reaction process was assessed using thin-layer chromatography (TLC). Following this analysis, 50 mL of cold water was added to the crude product, and the mixture was agitated for 15 minutes. Following the collection of the solid particles, the impure substance was refined using the process of silica gel chromatography, using a solvent mixture of 10% ethyl acetate in hexane. The organic layers, when mixed, were cleansed with brine, dehydrated with anhydrous Na_2_SO_4_, and ultimately condensed under reduced pressure to provide compound **4**. White solid (Yield 73%). ^1^H-NMR (400 MHz, DMSO-d_6_) δ 7.82 (d, *J* = 8.0 Hz, 1H), 7.17 (d, *J* = 8.0 Hz, 1H), 6.67 (s, 1H), 4.21 (d, *J* = 4.0 Hz, 2H, N-CH_2_), 3.60 (s, 3H, N-CH_3_), 3.10 (t, *J* = 4.0Hz, 1H), 2.16 (s, 3H, -CH_3_); ESI-MS: 281 [M+H]^+^.

**Synthesis of N-(3-iodoprop-2-yn-1-yl)-1-methyl-N-(5-methylisoxazol-3-yl)-1H-imidazole-2-sulfonamide (5)**

To 1-methyl-N-(5-methylisoxazol-3-yl)-N-(prop-2-yn-1-yl)-1H-imidazole-2-sulfonamide (4) (2g, 0.007 mol) was combined with THF (20 ml), CuI (2 mmol), and N-iodomorpholine (0.008 mol). The resulting reaction mixture was stirred at room temperature for 3 hours, yielding a fine white precipitate. The suspension was introduced onto a pad of activated neutral alumina (30 ml), and the resulting filtrate was subsequently gathered. The solid phase underwent washing with CH_2_Cl_2_ (3 × 30 ml), and the resultant organic fractions were concentrated through evaporation under reduced pressure, yielding compound **5**. Yield (67%); ^1^H-NMR (400 MHz, DMSO-d_6_) δ 7.82 (d, *J* = 8.0 Hz, 1H), 7.15 (d, *J* = 8.0 Hz, 1H), 6.65 (s, 1H), 4.22 (s, 2H, N-CH_2_), 3.60 (s, 3H, N-CH_3_), 2.18 (s, 3H, -CH_3_); ESI-MS: 407 [M+H]^+^.

**General procedure for the synthesis of imidazole-sulfonyl-1H-isoxazolo[3,4-b][1,2,3]triazolo[4,5-d]pyridine (6a-6o).**

A solution of alkyne (4) (0.001 mol) and aryl azide (5) (0.0012 mmol) in THF (10 mL) was mixed with CuI (10 mol%). The resulting mixture was agitated at room temperature for 8-10 hours. Following the completion of the reaction, the reaction mixture was mixed with cold water (15 mL), and the resulting product was extracted using ethyl acetate (2 × 20 mL). The organic layer, which comprised a combination of substances, was cleansed with brine and dehydrated using anhydrous Na_2_SO_4_. Following filtration, the solvent was removed under reduced pressure and the impure product obtained was subjected to column chromatography using a gradient of hexane and ethyl acetate. This process resulted in the isolation of compounds 6a-6o with high yields ranging from good to excellent.

**8-methyl-5-((1-methyl-1H-imidazol-2-yl)sulfonyl)-1-phenyl-4,5-dihydro-1H-isoxazolo[3,4-b][1,2,3]triazolo[4,5-d]pyridine (6a):** Dirty white solid. M.p: 133-135 ^o^C; ^1^H-NMR (400 MHz, DMSO-d*_6_*) δ 7.84 (d, *J* = 8.0 Hz, 1H), 7.64 (d, J=8.0Hz, 2H), 7.33-7.28 (m, 3H), 7.16 (d, *J* = 8.0 Hz, 1H), 5.11 (s, 2H, N-CH_2_), 3.62 (s, 3H, N-CH_3_), 2.18 (s, 3H, -CH_3_); ^13^C-NMR (100 MHz, DMSO-d*_6_*): δ 171.24, 161.38, 158.67, 137.83, 129.40(2C), 128.83, 126.37, 125.41, 124.29(2C), 122.42, 121.26, 113.26, 42.31, 36.25, 16.63; ESI-MS: 398 [M+H]^+^. Anal. Calcd for C_17_H_15_N_7_O_3_S: C, 51.38; H, 3.80; N, 24.67. Found: C, 51.35; H, 3.77; N, 24.69.

**1-(4-methoxyphenyl)-8-methyl-5-((1-methyl-1H-imidazol-2-yl)sulfonyl)-4,5-dihydro-1H-isoxazolo[3,4-b][1,2,3]triazolo[4,5-d]pyridine (6b):** White solid. M.p: 158-160 ^o^C; ^1^H-NMR (400 MHz, DMSO-d_6_) δ 7.83 (d, *J* = 8.0 Hz, 1H), 7.70 (d, J=8.0Hz, 2H), 7.16 (d, *J* = 8.0 Hz, 1H), 7.00 (d, J=8.0Hz, 2H), 5.10 (s, 2H, N-CH_2_), 3.84 (s, 3H, OCH_3_), 3.62 (s, 3H, N-CH_3_), 2.18 (s, 3H, -CH_3_); ^13^C-NMR (100 MHz, DMSO-d_6_) δ 171.38, 161.33, 159.68, 158.34, 131.43, 127.04(2C), 126.58, 125.95, 122.54, 121.18, 114.41(2C), 113.26, 56.15, 42.57, 36.35, 16.46; ESI-MS: 428 [M+H]^+^. Anal. Calcd for C_18_H_17_N_7_O_4_S: C, 50.58; H, 4.01; N, 22.94. Found: C, 50.55; H, 3.98; N, 22.96.

**1-(4-chlorophenyl)-8-methyl-5-((1-methyl-1H-imidazol-2-yl)sulfonyl)-4,5-dihydro-1H-isoxazolo[3,4-b][1,2,3]triazolo[4,5-d]pyridine (6c):** Yellow solid. M.p: 145-147 ^o^C; ^1^H-NMR (400 MHz, DMSO-d_6_) δ 7.85 (d, *J* = 8.0 Hz, 1H), 7.73 (d, J=8.0Hz, 2H), 7.39 (d, J=8.0Hz, 2H), 7.17 (d, *J* = 8.0 Hz, 1H), 5.12 (s, 2H, N-CH_2_), 3.63 (s, 3H, N-CH_3_), 2.19 (s, 3H, -CH_3_); ^13^C-NMR (100 MHz, DMSO-d_6_): δ 171.30, 161.38, 158.36, 136.43, 133.39, 128.73(2C), 126.85, 125.36(2C), 124.32, 122.46, 121.31, 113.75, 42.68, 36.29, 16.79; ESI-MS: 432 [M+H]^+^. Anal. Calcd for C_17_H_14_ClN_7_O_3_S: C, 47.28; H, 3.27; N, 22.70. Found: C, 47.25; H, 3.25; N, 22.73.

**1-(4-fluorophenyl)-8-methyl-5-((1-methyl-1H-imidazol-2-yl)sulfonyl)-4,5-dihydro-1H-isoxazolo[3,4-b][1,2,3]triazolo[4,5-d]pyridine (6d):** Red solid. M.p: 139-141 ^o^C; ^1^H-NMR (400 MHz, DMSO-d_6_) δ 8.22 (d, J=8.0Hz, 2H), 7.98 (d, J=8.0Hz, 2H), 7.85 (d, *J* = 8.0 Hz, 1H), 7.16 (d, *J* = 8.0 Hz, 1H), 5.12 (s, 2H, N-CH_2_), 3.61 (s, 3H, N-CH_3_), 2.20 (s, 3H, -CH_3_); ^13^C-NMR (100 MHz, DMSO-d_6_): δ 171.24, 162.49, 161.24, 160.37, 158.36, 133.57, 126.81, 126.75, 125.41, 124.93, 122.59, 121.61, 116.73, 116.57, 113.26, 42.31, 36.33, 16.84; ESI-MS: 416 [M+H]^+^. Anal. Calcd for C_17_H_14_FN_7_O_3_S: C, 49.15; H, 3.40; N, 23.60. Found: C, 49.13; H, 3.38; N, 23.62.

**1-(4-bromophenyl)-8-methyl-5-((1-methyl-1H-imidazol-2-yl)sulfonyl)-4,5-dihydro-1H-isoxazolo[3,4-b][1,2,3]triazolo[4,5-d]pyridine (6e):** White solid. M.p: 153-155 ^o^C; ^1^H-NMR (400 MHz, DMSO-d_6_) δ 7.84 (d, *J* = 8.0 Hz, 1H), 7.64 (d, J=8.0Hz, 2H), 7.49 (d, J=8.0Hz, 2H), 7.16 (d, *J* = 8.0 Hz, 1H), 5.12 (s, 2H, N-CH_2_), 3.61 (s, 3H, N-CH_3_), 2.18 (s, 3H, -CH_3_); ^13^C-NMR (100 MHz, DMSO-d_6_): δ 171.56, 161.57, 158.47, 135.79, 132.31(2C), 126.77, 125.49, 124.22(2C), 122.23, 121.34, 120.66, 113.82, 42.57, 36.33, 16.81; ESI-MS: 476 [M+H]^+^. Anal. Calcd for C_17_H_14_BrN_7_O_3_S: C, 42.87; H, 2.96; N, 20.58. Found: C, 42.85; H, 2.94; N, 20.60.

**4-(8-methyl-5-((1-methyl-1H-imidazol-2-yl)sulfonyl)-4,5-dihydro-1H-isoxazolo[3,4-b][1,2,3]triazolo[4,5-d]pyridin-1-yl)benzonitrile (6f):** Pale red solid. M.p: 141-143 ^o^C; ^1^H-NMR (400 MHz, DMSO-d_6_) δ 7.92 (d, J=8.0Hz, 2H), 7.83 (d, *J* = 8.0 Hz, 1H), 7.51 (d, *J*=8.0 Hz, 2H), 7.17 (d, *J* = 8.0 Hz, 1H), 5.11 (s, 2H, N-CH_2_), 3.62 (s, 3H, N-CH_3_), 2.19 (s, 3H, -CH_3_);  ^13^C-NMR (100 MHz, DMSO-d_6_): δ 171.37, 161.58, 158.40, 139.47, 127.71(2C), 126.43(2C), 125.68, 125.40, 122.77, 121.60, 119.59, 116.96, 113.31, 42.28, 36.64, 16.82; ESI-MS: 423 [M+H]^+^. Anal. Calcd for C_18_H_14_N_8_O_3_S: C, 51.18; H, 3.34; N, 26.53. Found: C, 51.16; H, 3.31; N, 26.55.

**8-methyl-5-((1-methyl-1H-imidazol-2-yl)sulfonyl)-1-(4-nitrophenyl)-4,5-dihydro-1H-isoxazolo[3,4-b][1,2,3]triazolo[4,5-d]pyridine (6g):** Yellow solid. M.p: 148-150 ^o^C; ^1^H-NMR (400 MHz, DMSO-d_6_) δ 8.34 (d, J=8.0Hz, 2H), 8.14 (d, J=8.0Hz, 2H), 7.85 (d, *J* = 8.0 Hz, 1H), 7.18 (d, *J* = 8.0 Hz, 1H), 5.12 (s, 2H, N-CH_2_), 3.63 (s, 3H, N-CH_3_), 2.20 (s, 3H, -CH_3_);  ^13^C-NMR (100 MHz, DMSO-d_6_): δ 171.17, 161.62, 158.44, 148.71, 141.50, 127.73(2C), 126.45, 125.44, 124.32(2C), 122.38, 121.21, 113.33, 42.28, 36.65, 16.47; ESI-MS: 443 [M+H]^+^ . Anal. Calcd for C_17_H_14_N_8_O_5_S: C, 46.15; H, 3.19; N, 25.33. Found: C, 46.13; H, 3.16; N, 25.37.

**1-(3,4-dichlorophenyl)-8-methyl-5-((1-methyl-1H-imidazol-2-yl)sulfonyl)-4,5-dihydro-1H-isoxazolo[3,4-b][1,2,3]triazolo[4,5-d]pyridine (6h):** Pale Yellow solid. M.p: 160-162 ^o^C; ^1^H-NMR (400 MHz, DMSO-d_6_) δ 7.85 (d, *J* = 8.0 Hz, 1H), 7.73 (s, 1H), 7.62 (d, J=8.0Hz, 1H), 7.45 (d, J=8.0Hz, 1H), 7.16 (d, *J* = 8.0 Hz, 1H), 5.11 (s, 2H, N-CH_2_), 3.61 (s, 3H, N-CH_3_), 2.18 (s, 3H, -CH_3_); ^13^C-NMR (100 MHz, DMSO-d_6_): δ 171.52, 161.34, 158.42, 138.06, 133.38, 131.61, 130.61, 126.73, 125.38, 124.80, 123.36, 122.48, 121.19, 113.30, 42.58, 36.61, 16.43; ESI-MS: 466 [M+H]^+^. Anal. Calcd for C_17_H_13_Cl_2_N_7_O_3_S: C, 43.79; H, 2.81; N, 21.03. Found: C, 43.76; H, 2.78; N, 21.05.

**1-(3,5-dichlorophenyl)-8-methyl-5-((1-methyl-1H-imidazol-2-yl)sulfonyl)-4,5-dihydro-1H-isoxazolo[3,4-b][1,2,3]triazolo[4,5-d]pyridine (6i):** Pale Yellow solid. M.p: 167-169 ^o^C; ^1^H-NMR (400 MHz, DMSO-d_6_) δ 7.85 (d, *J* = 8.0 Hz, 1H), 7.66 (s, 2H), 7.37 (s, 1H), 7.17 (d, *J* = 8.0 Hz, 1H), 5.12 (s, 2H, N-CH_2_), 3.62 (s, 3H, N-CH_3_), 2.18 (s, 3H, -CH_3_);  ^13^C-NMR (100 MHz, DMSO-d_6_): δ 171.34, 161.15, 158.41, 139.51, 134.42(2C), 126.69, 125.90, 124.84, 123.46(2C), 122.40, 121.41, 113.28, 42.59, 36.29, 16.43; ESI-MS: 466 [M+H]^+^. Anal. Calcd for C_17_H_13_Cl_2_N_7_O_3_S: C, 43.79; H, 2.81; N, 21.03. Found: C, 43.76; H, 2.79; N, 21.05.

**1-(2,4-dichlorophenyl)-8-methyl-5-((1-methyl-1H-imidazol-2-yl)sulfonyl)-4,5-dihydro-1H-isoxazolo[3,4-b][1,2,3]triazolo[4,5-d]pyridine (6j):** Pale yellow solid. M.p: 164-166 ^o^C; ^1^H-NMR (400 MHz, DMSO-d_6_) δ 7.85 (d, *J* = 8.0 Hz, 1H), 7.70 (s, 1H), 7.62 (d, J=8.0Hz, 1H), 7.43 (d, J=8.0Hz, 1H),7.17 (d, *J* = 8.0 Hz, 1H), 5.12 (s, 2H, N-CH_2_), 3.62 (s, 3H, N-CH_3_), 2.19 (s, 3H, -CH_3_); ^13^C-NMR (100 MHz, DMSO-d_6_): δ 171.33, 161.40, 158.35, 136.45, 134.52, 133.52, 131.35, 130.33, 128.77, 126.35, 125.54, 121.82, 120.73, 113.28, 42.59, 36.32, 16.82; ESI-MS: 466 [M+H]^+^. Anal. Calcd for C_17_H_13_Cl_2_N_7_O_3_S: C, 43.79; H, 2.81; N, 21.03. Found: C, 43.75; H, 2.78; N, 21.06.

**1-(3,5-dichloro-4-fluorophenyl)-8-methyl-5-((1-methyl-1H-imidazol-2-yl)sulfonyl)-4,5-dihydro-1H-isoxazolo[3,4-b][1,2,3]triazolo[4,5-d]pyridine (6k):** Red solid. M.p: 173-175 ^o^C; ^1^H-NMR (400 MHz, DMSO-d_6_) δ 7.85 (d, *J* = 8.0 Hz, 1H), 7.72 (s, 2H), 7.17 (d, *J* = 8.0 Hz, 1H), 5.12 (s, 2H, N-CH_2_), 3.62 (s, 3H, N-CH_3_), 2.20 (s, 3H, -CH_3_); ^13^C-NMR (100 MHz, DMSO-d_6_): δ 171.42, 161.79, 158.46, 156.43, 154.32, 134.78, 126.74, 125.73, 125.28, 124.90, 124.31, 124.25, 122.21, 121.20, 113.35, 42.60, 36.36, 16.49; ESI-MS: 484 [M+H]^+^. Anal. Calcd for C_17_H_12_C_l2_FN_7_O_3_S: C, 42.16; H, 2.50; N, 20.25. Found: C, 42.15; H, 2.48; N, 20.27.

**1-(3,5-dibromophenyl)-8-methyl-5-((1-methyl-1H-imidazol-2-yl)sulfonyl)-4,5-dihydro-1H-isoxazolo[3,4-b][1,2,3]triazolo[4,5-d]pyridine (6l):** White solid. M.p: 167-169 ^o^C; ^1^H-NMR (400 MHz, DMSO-d_6_) δ 7.84 (d, *J* = 8.0 Hz, 1H), 7.57 (s, 2H), 7.39 (s, 1H), 7.17 (d, *J* = 8.0 Hz, 1H), 5.11 (s, 2H, N-CH_2_), 3.61 (s, 3H, N-CH_3_), 2.18 (s, 3H, -CH_3_); ^13^C-NMR (100 MHz, DMSO-d_6_): δ 171.50, 161.30, 158.40, 138.39, 130.64(2C), 126.63, 125.98, 124.90(2C), 123.16, 122.21, 121.20, 113.32, 42.47, 36.59, 16.48; ESI-MS: 556 [M+3H]^+^. Anal. Calcd for C_17_H_13_Br_2_N_7_O_3_S: C, 36.78; H, 2.36; N, 17.66. Found: C, 36.75; H, 2.34; N, 17.68.

**1-(3,5-dinitrophenyl)-8-methyl-5-((1-methyl-1H-imidazol-2-yl)sulfonyl)-4,5-dihydro-1H-isoxazolo[3,4-b][1,2,3]triazolo[4,5-d]pyridine (6m):** Yellow solid. M.p: 172-174 ^o^C; ^1^H-NMR (400 MHz, DMSO-d_6_) δ 8.33 (s, 2H), 8.11 (s, 1H), 7.87 (d, *J* = 8.0 Hz, 1H), 7.20 (d, *J* = 8.0 Hz, 1H), 5.13 (s, 2H, N-CH_2_), 3.63 (s, 3H, N-CH_3_), 2.20 (s, 3H, -CH_3_); ^13^C-NMR (100 MHz, DMSO-d_6_): δ 171.33, 161.68, 158.36, 149.63(2C), 137.24, 126.85, 126.31, 125.45(2C), 124.52, 122.72, 121.24, 113.35, 42.56, 36.63, 16.80; ESI-MS: 488 [M+H]^+^. Anal. Calcd for C_17_H_13_N_9_O_7_S: C, 41.89; H, 2.69; N, 25.86. Found: C, 41.84; H, 2.66; N, 25.88.

**1-(4-chloro-3,5-dinitrophenyl)-8-methyl-5-((1-methyl-1H-imidazol-2-yl)sulfonyl)-4,5-dihydro-1H-isoxazolo[3,4-b][1,2,3]triazolo[4,5-d]pyridine (6n):** Yellow solid. M.p: 178-180 ^o^C; ^1^H-NMR (400 MHz, DMSO-d_6_) δ 8.28 (s, 2H), 7.85 (d, *J* = 8.0 Hz, 1H), 7.19 (d, *J* = 8.0 Hz, 1H), 5.14 (s, 2H, N-CH_2_), 3.63 (s, 3H, N-CH_3_), 2.21 (s, 3H, -CH_3_); ^13^C-NMR (100 MHz, DMSO-d_6_): δ 171.28, 161.32, 158.30, 140.32(2C), 137.10, 126.91, 126.39(2C), 125.24, 123.61, 122.75, 121.25, 113.22, 42.58, 36.33, 16.43; ESI-MS: 522 [M+H]^+^. Anal. Calcd for C_17_H_12_ClN_9_O_7_S: C, 39.13; H, 2.32; N, 24.16. Found: C, 39.10; H, 2.30; N, 24.19.

**5-(8-methyl-5-((1-methyl-1H-imidazol-2-yl)sulfonyl)-4,5-dihydro-1H-isoxazolo[3,4-b][1,2,3]triazolo[4,5-d]pyridin-1-yl)isophthalonitrile (6o):** White solid. M.p: 161-163 ^o^C; ^1^H-NMR (400 MHz, DMSO-d_6_) δ 7.84 (d, *J* = 8.0 Hz, 1H), 7.77 (s, 2H), 7.47 (s, 1H), 7.18 (d, *J* = 8.0 Hz, 1H), 5.12 (s, 2H, N-CH_2_), 3.62 (s, 3H, N-CH_3_), 2.19 (s, 3H, -CH_3_); ^13^C-NMR (100 MHz, DMSO-d_6_): δ 171.66, 161.46, 158.30, 133.55, 128.44(2C), 128.23, 126.40, 125.28, 122.73, 121.67, 118.62(2C), 116.74(2C), 113.23, 42.22, 36.54, 16.54; ESI-MS: 448 [M+H]^+^. Anal. Calcd for C_19_H_13_N_9_O_3_S: C, 51.00; H, 2.93; N, 28.17. Found: C, 50.98; H, 2.89; N, 28.21.


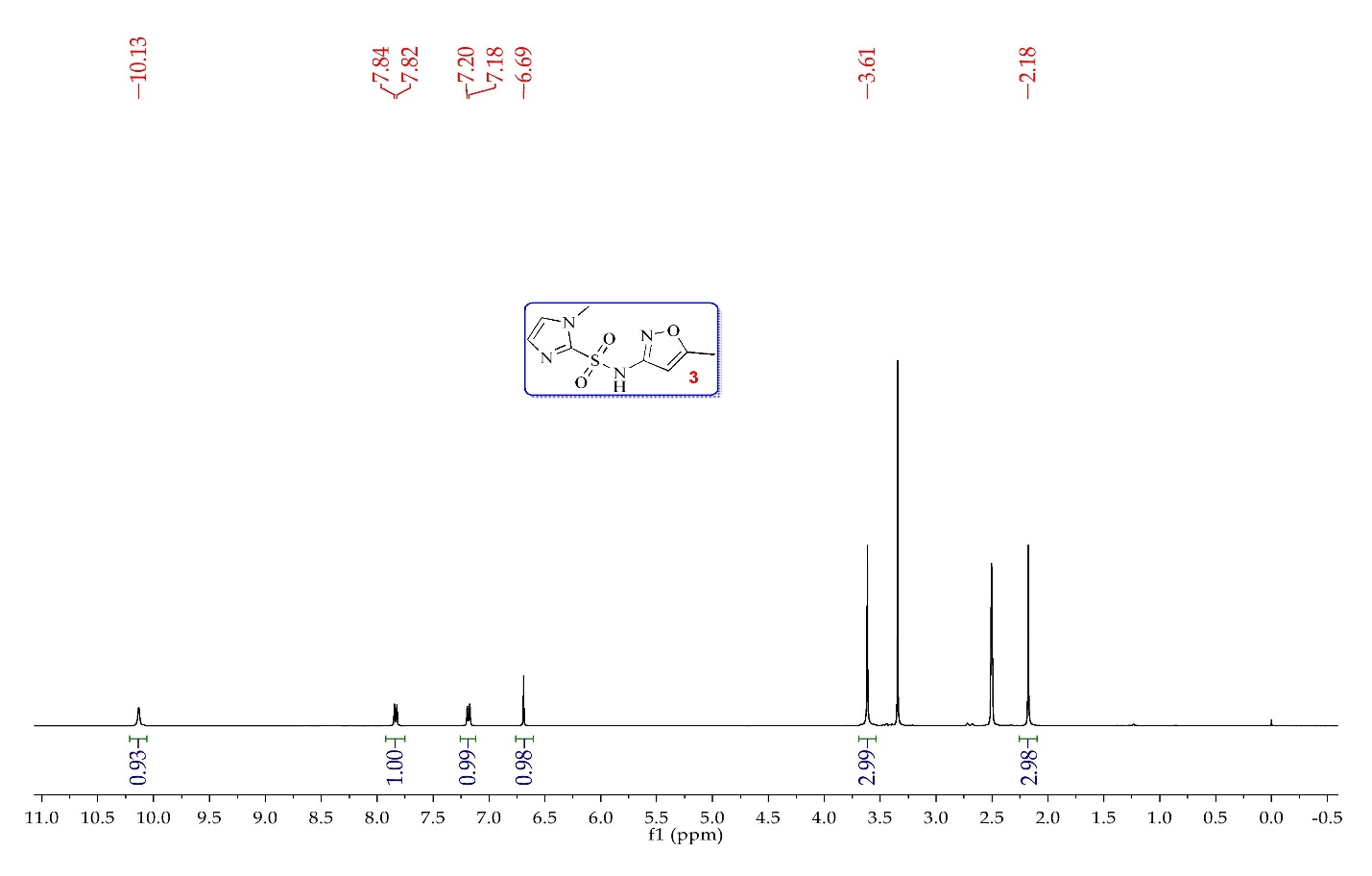

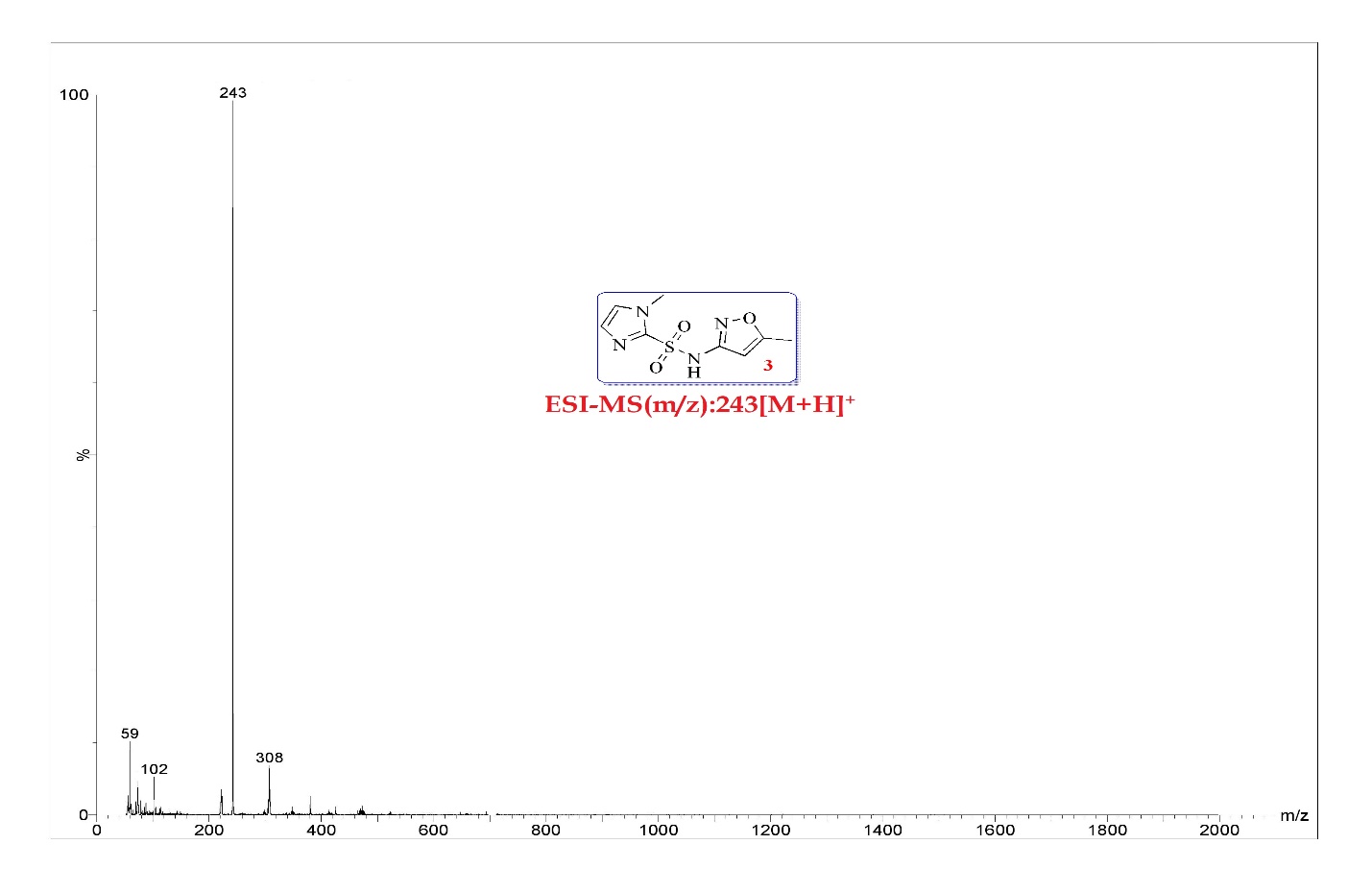

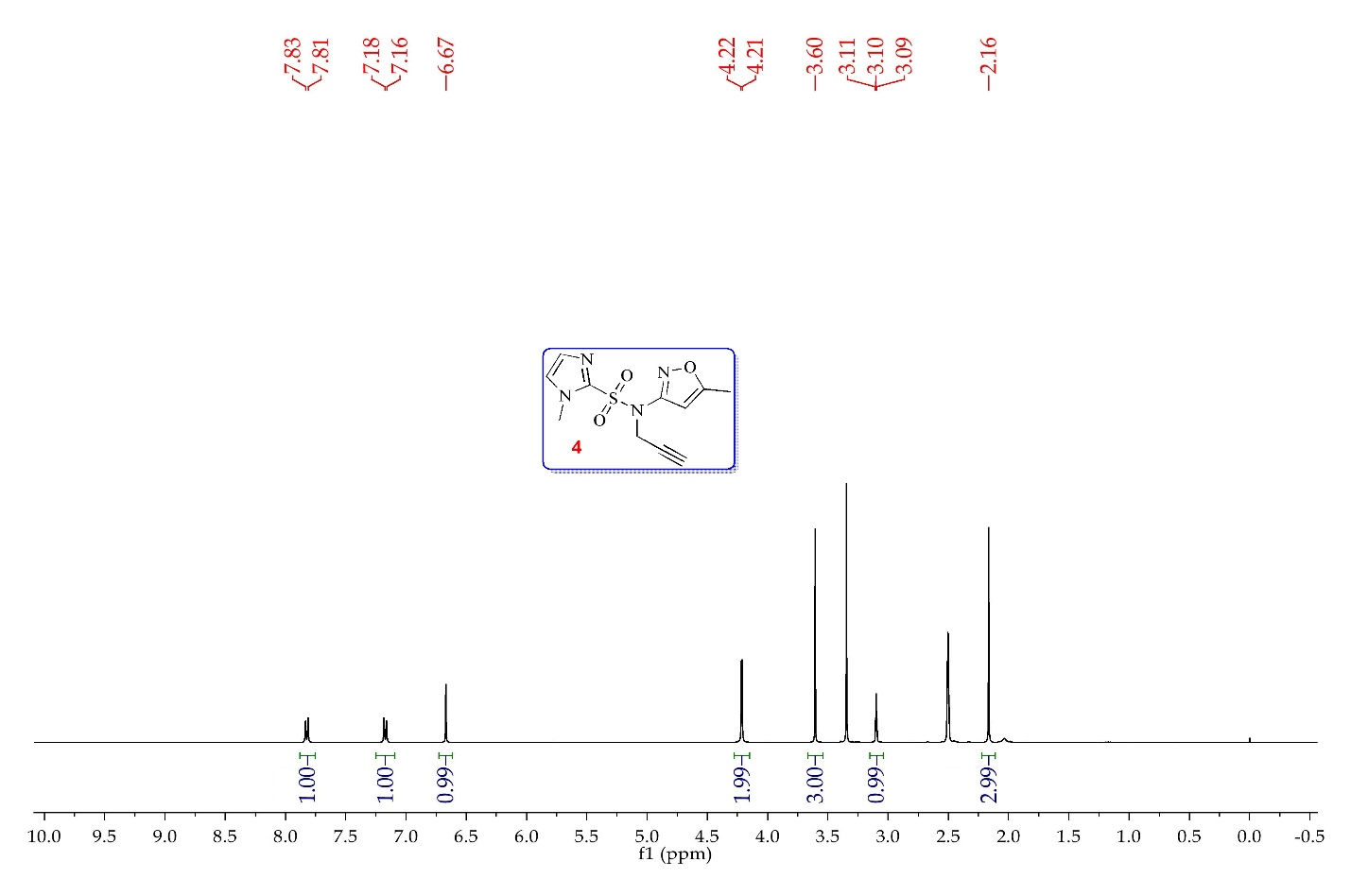

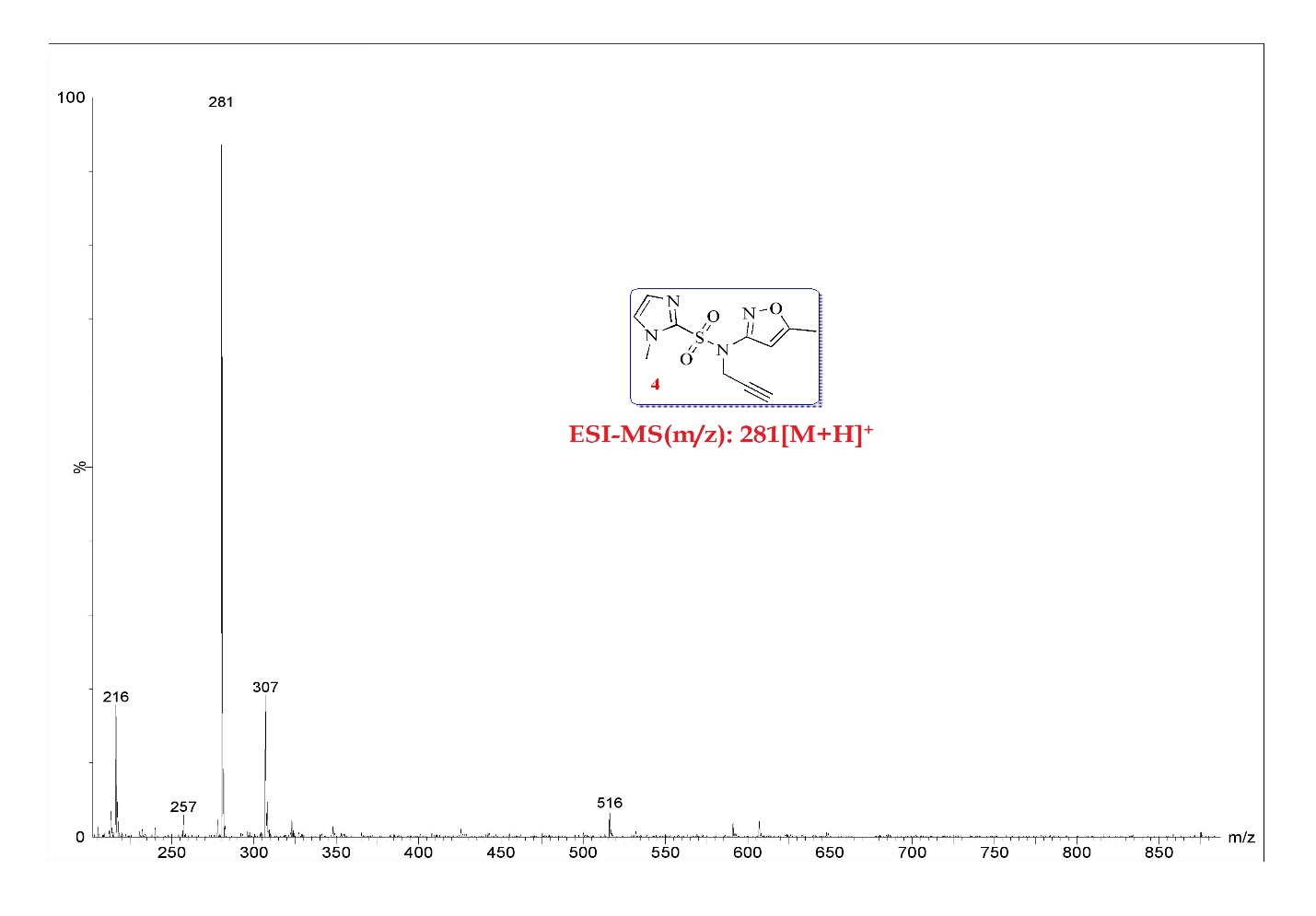


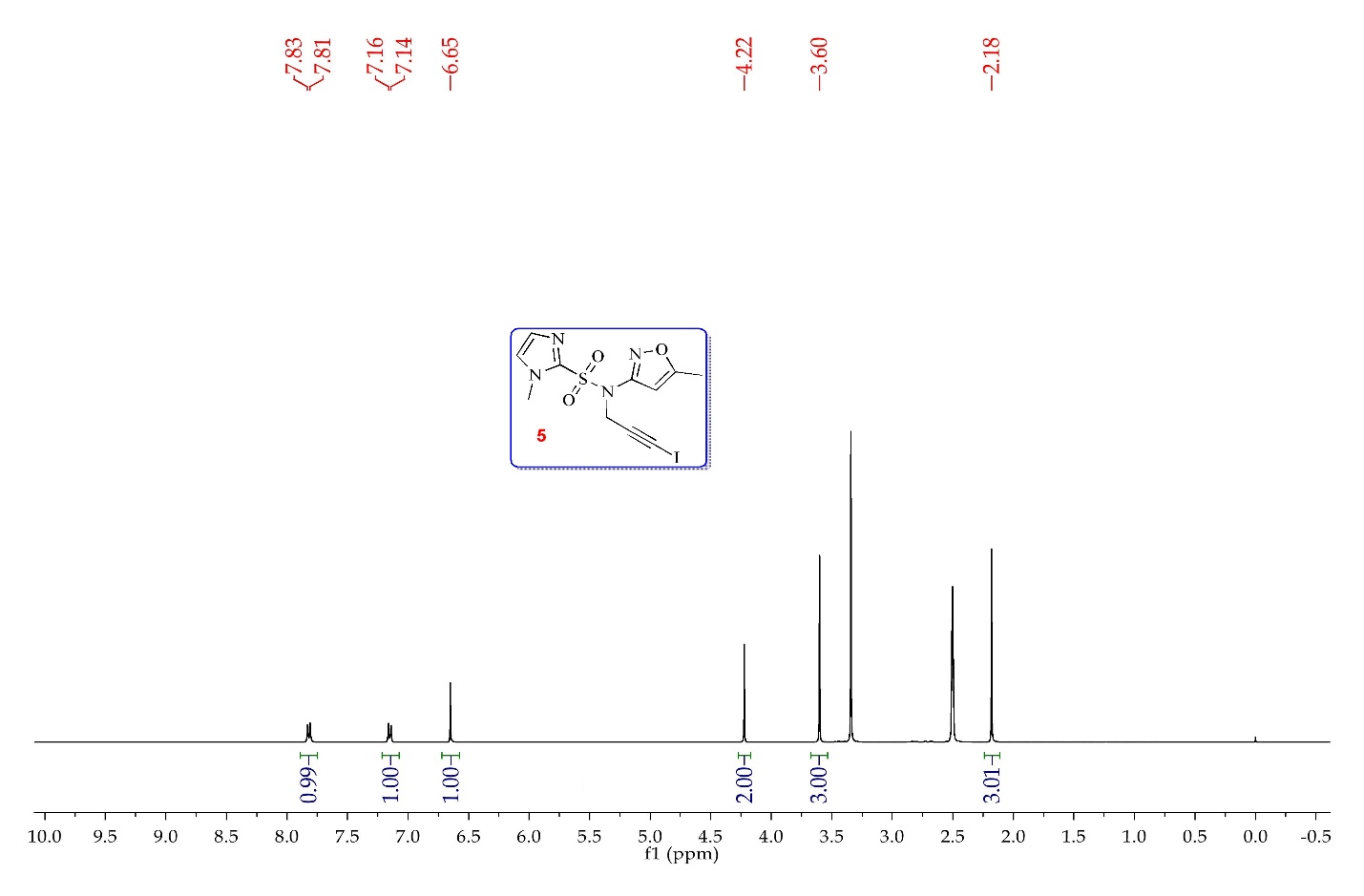

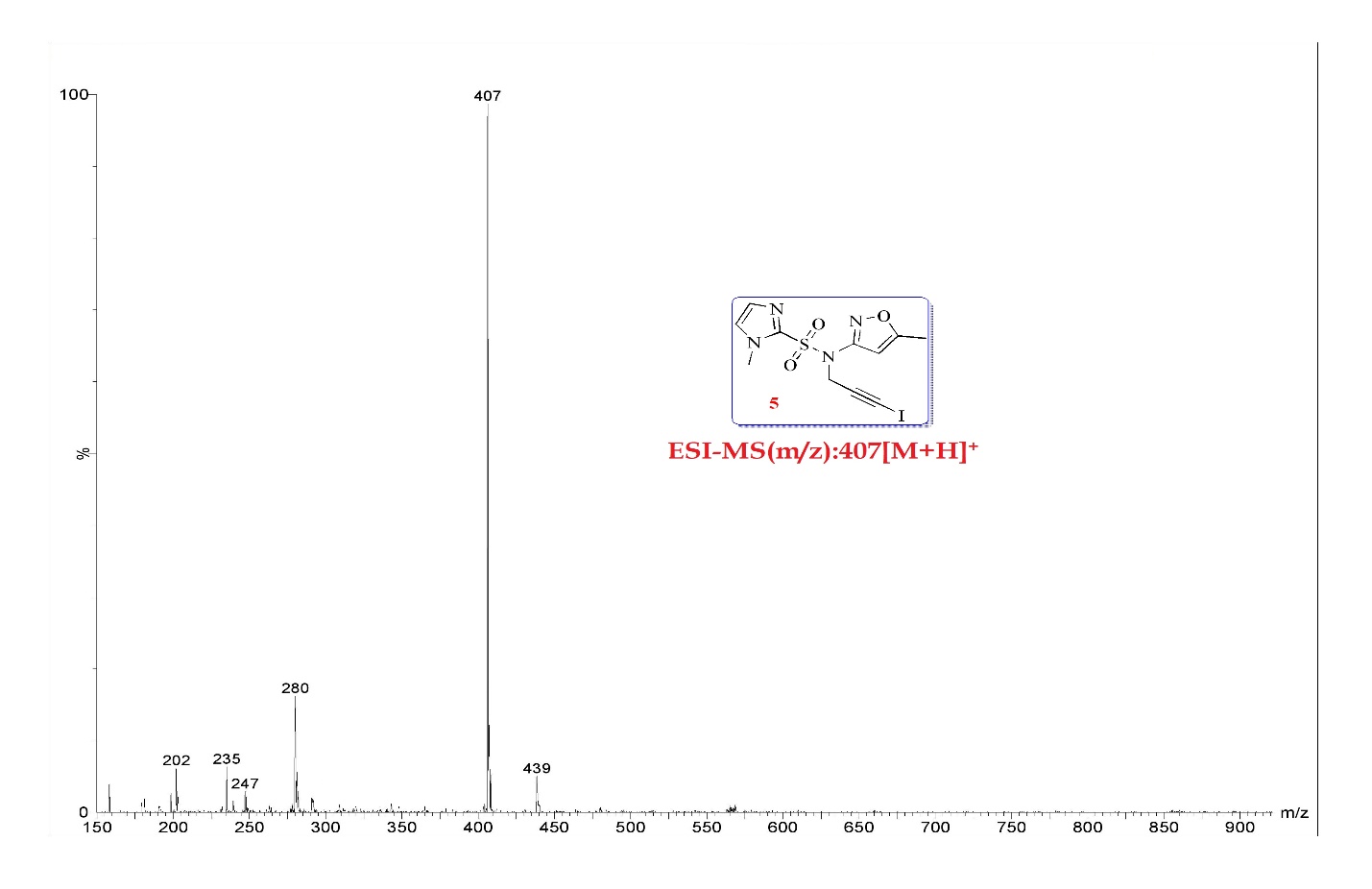


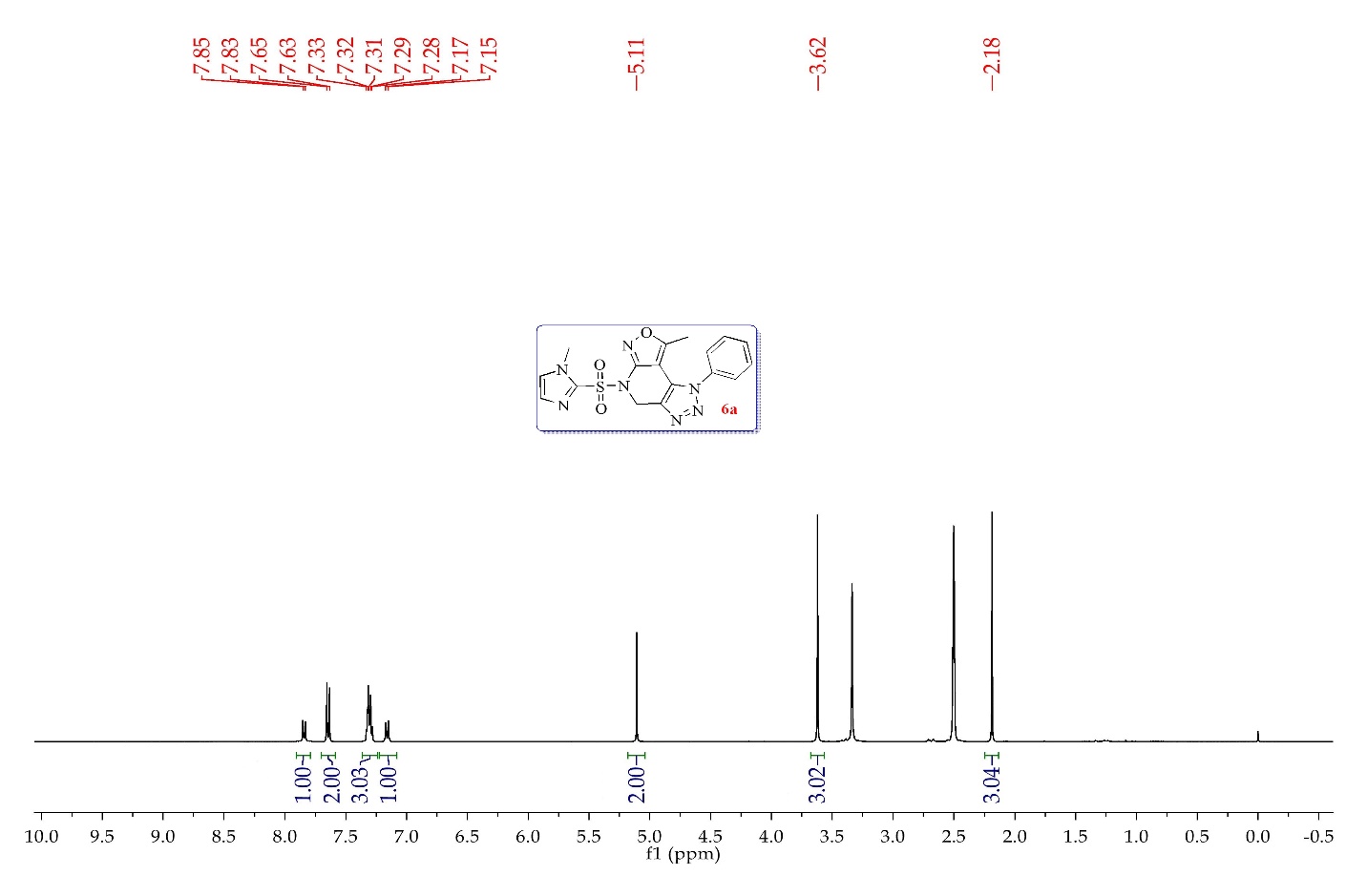

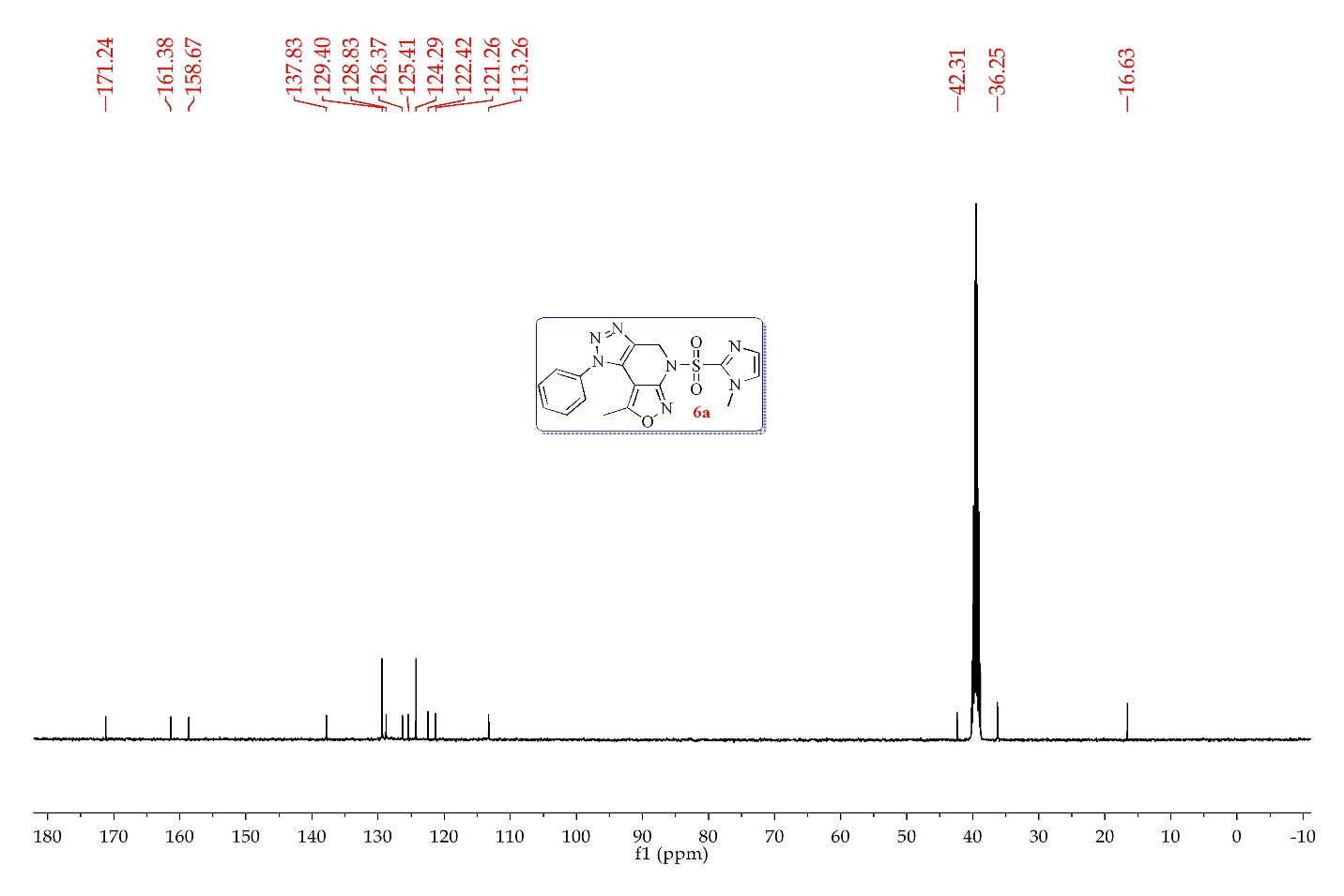

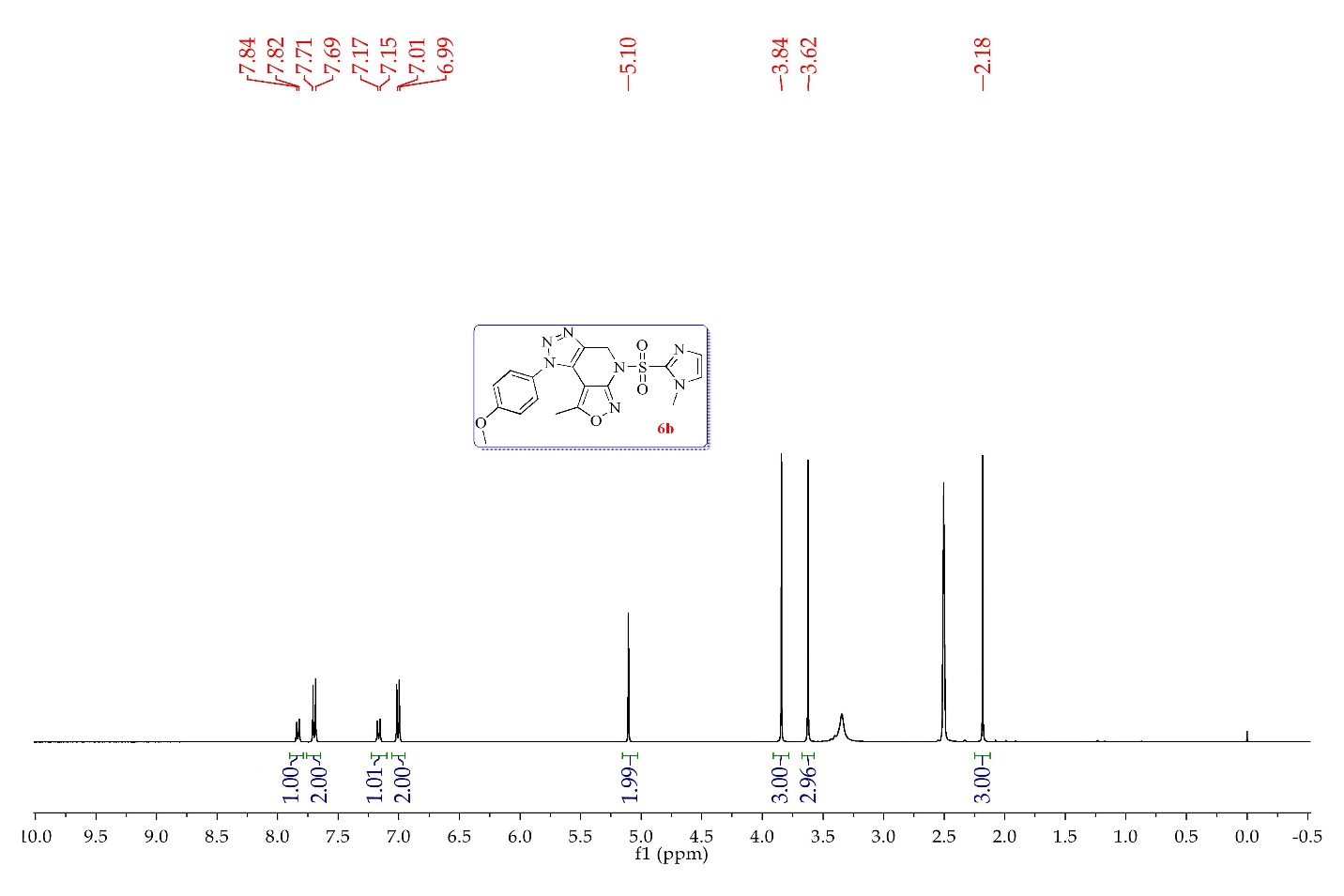

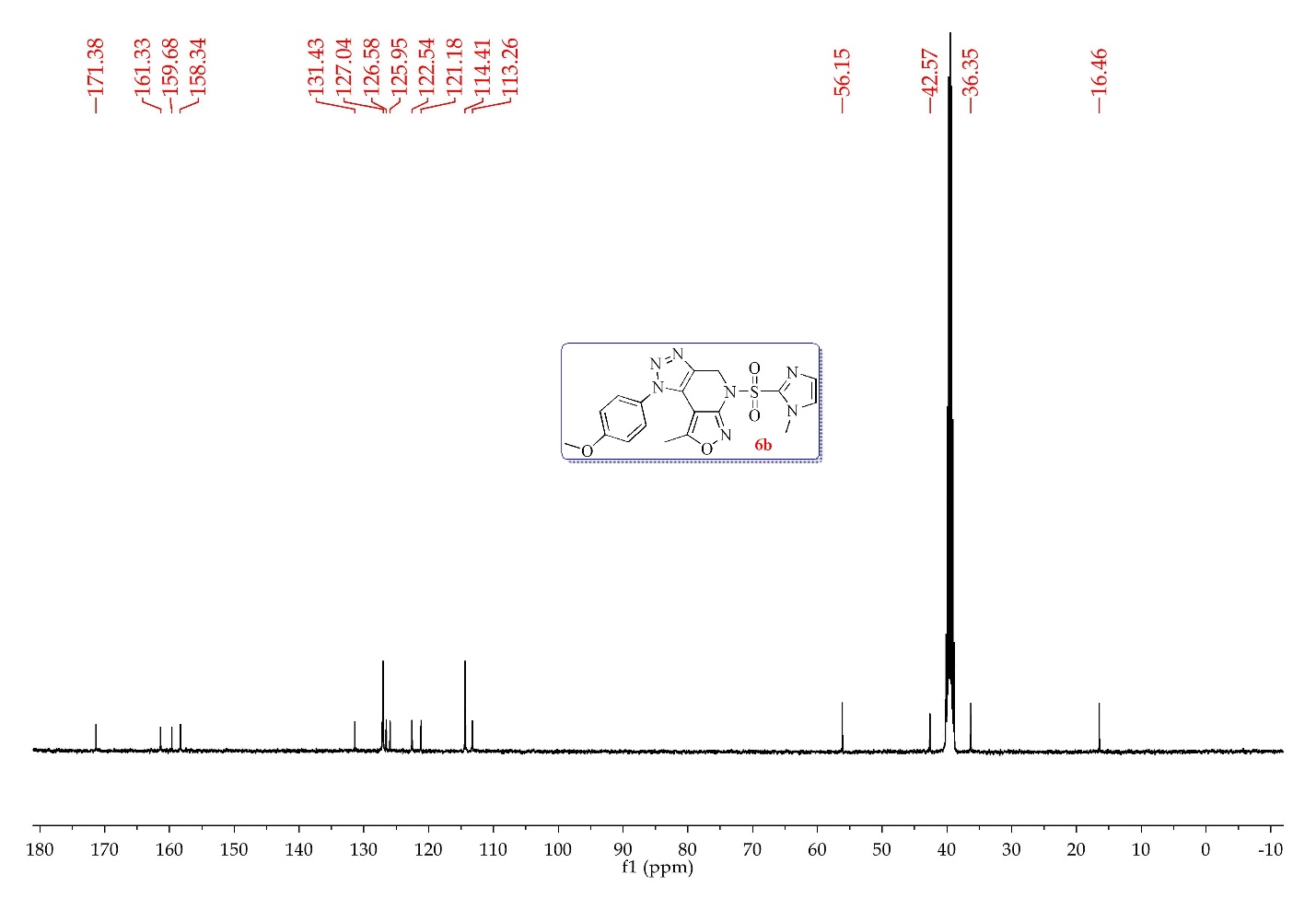

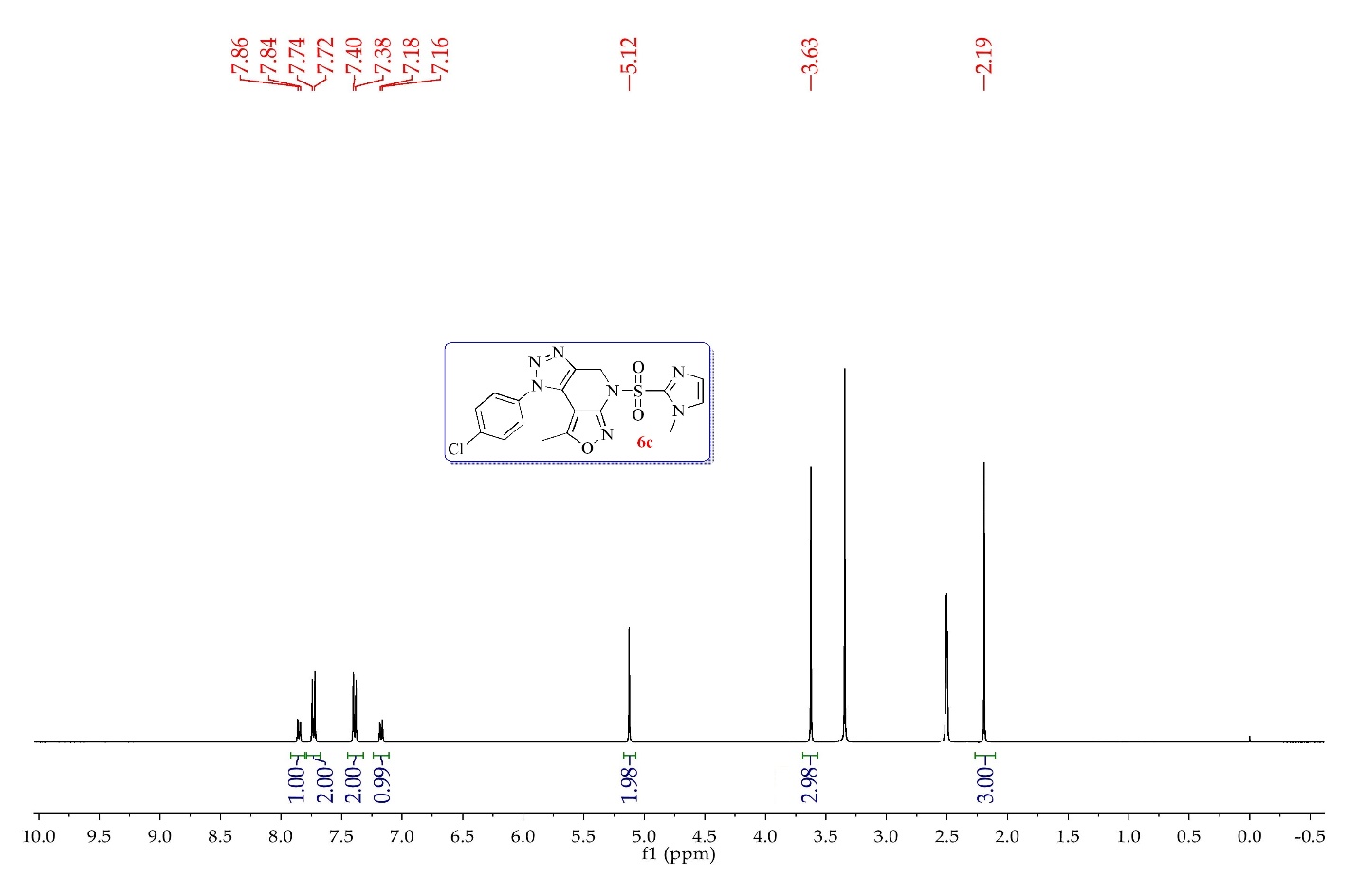

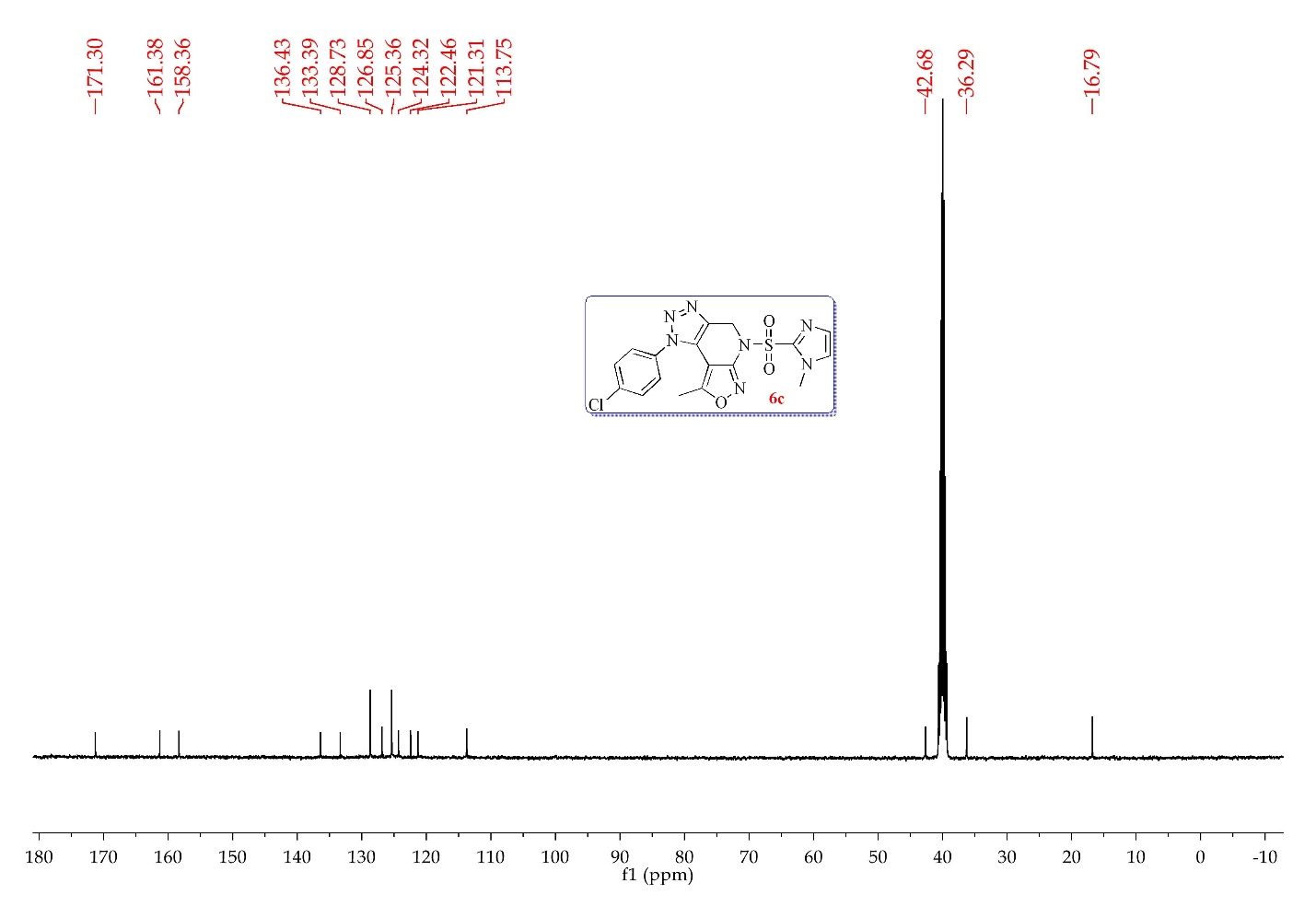

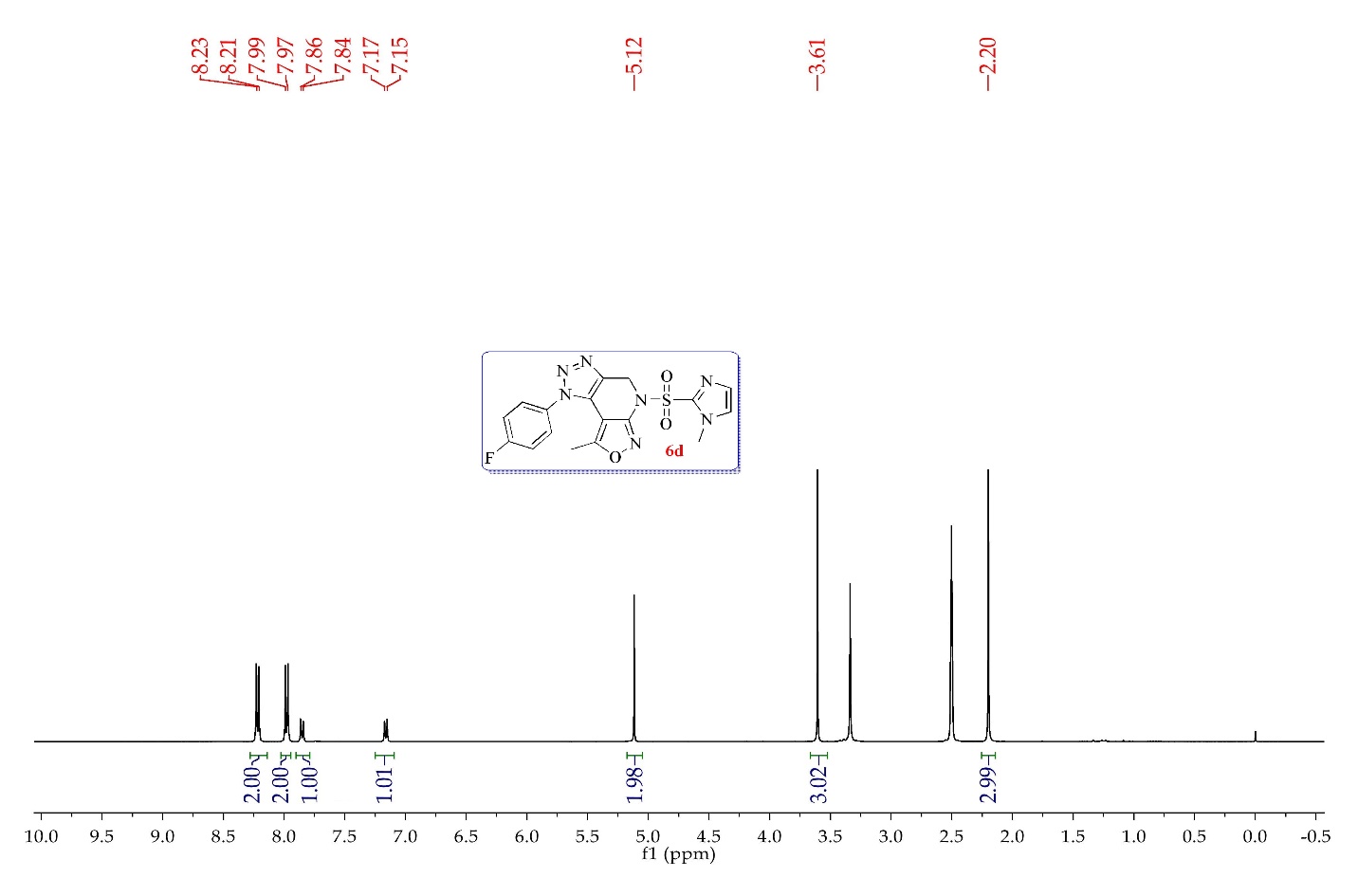

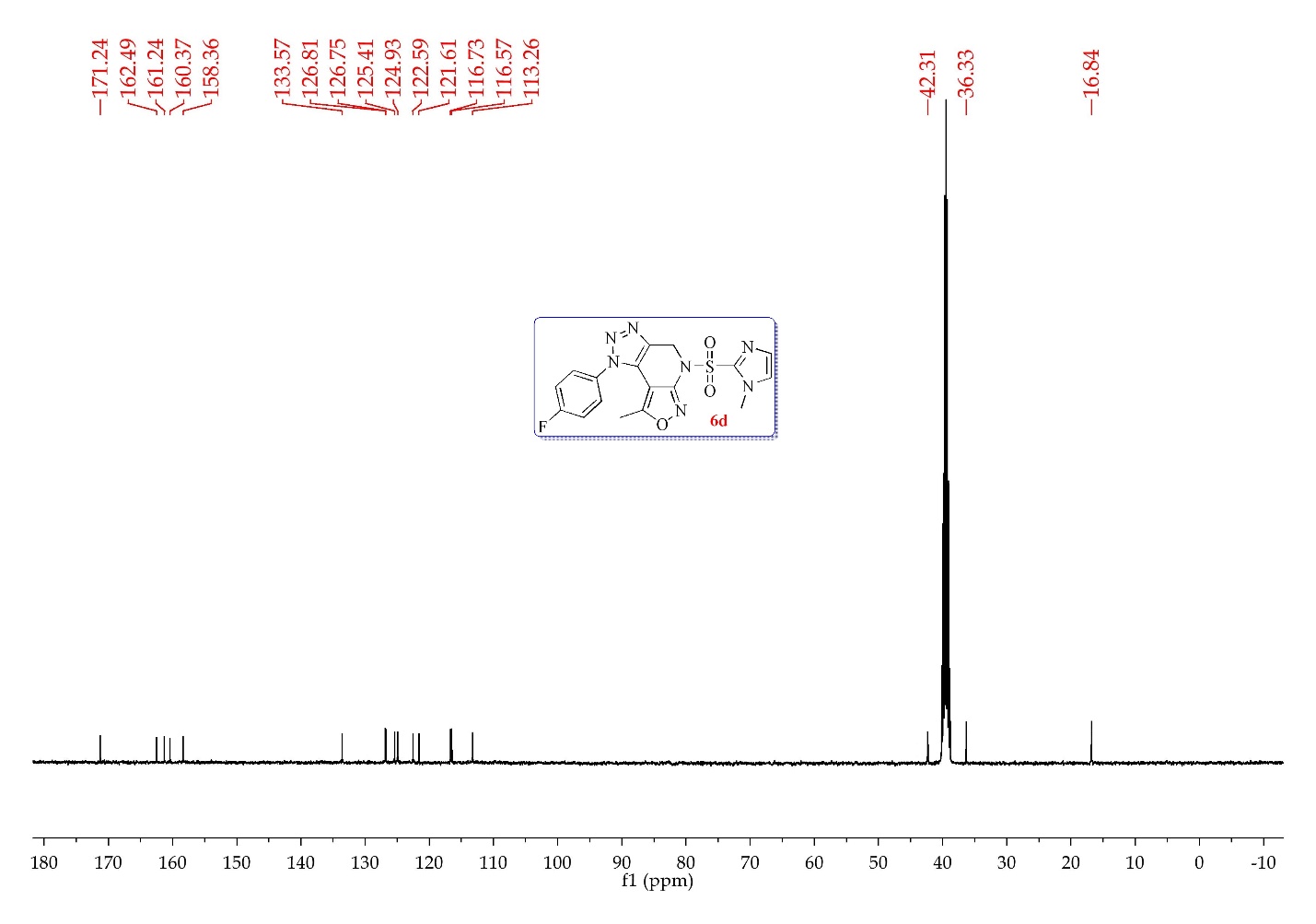

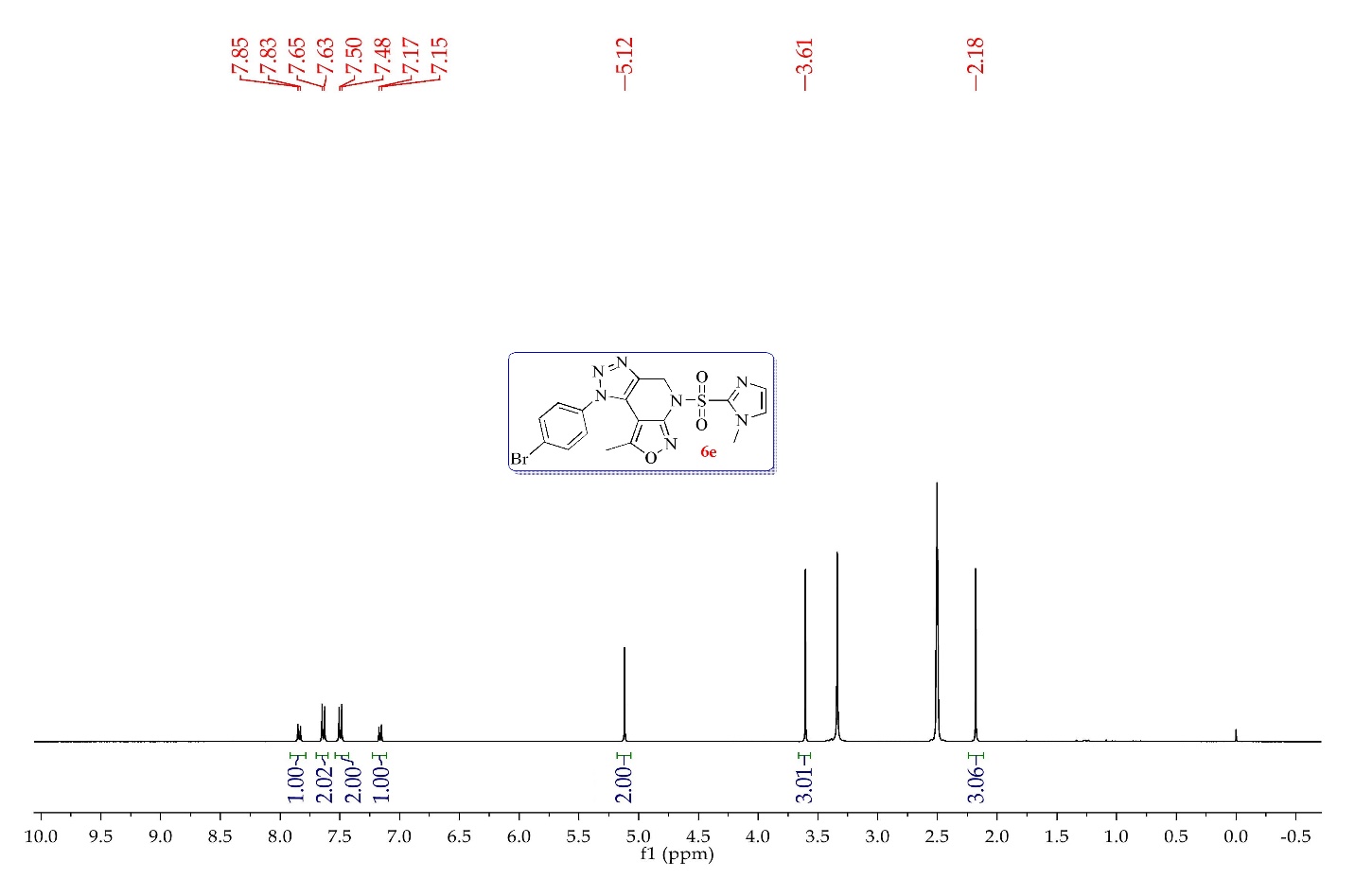

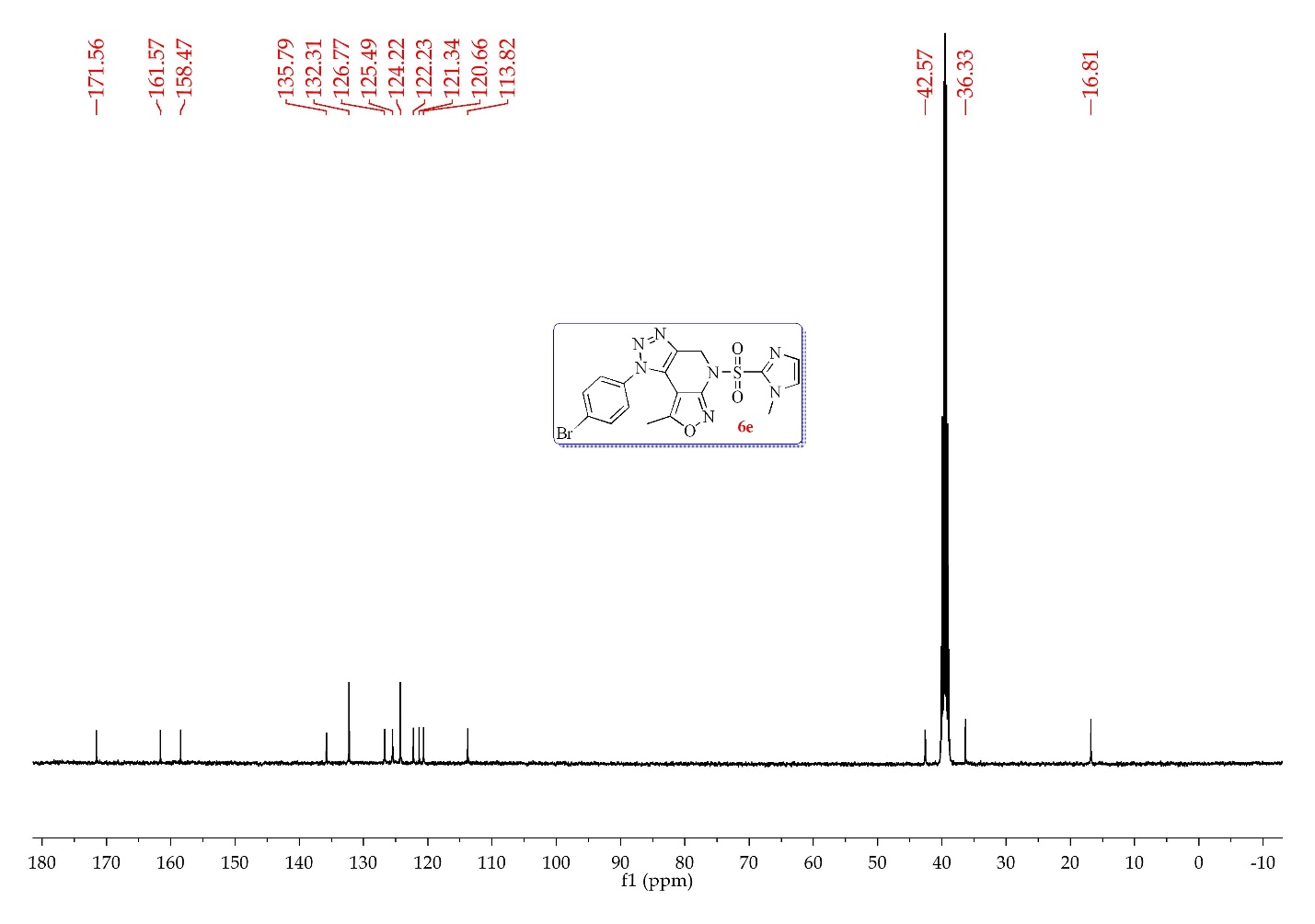

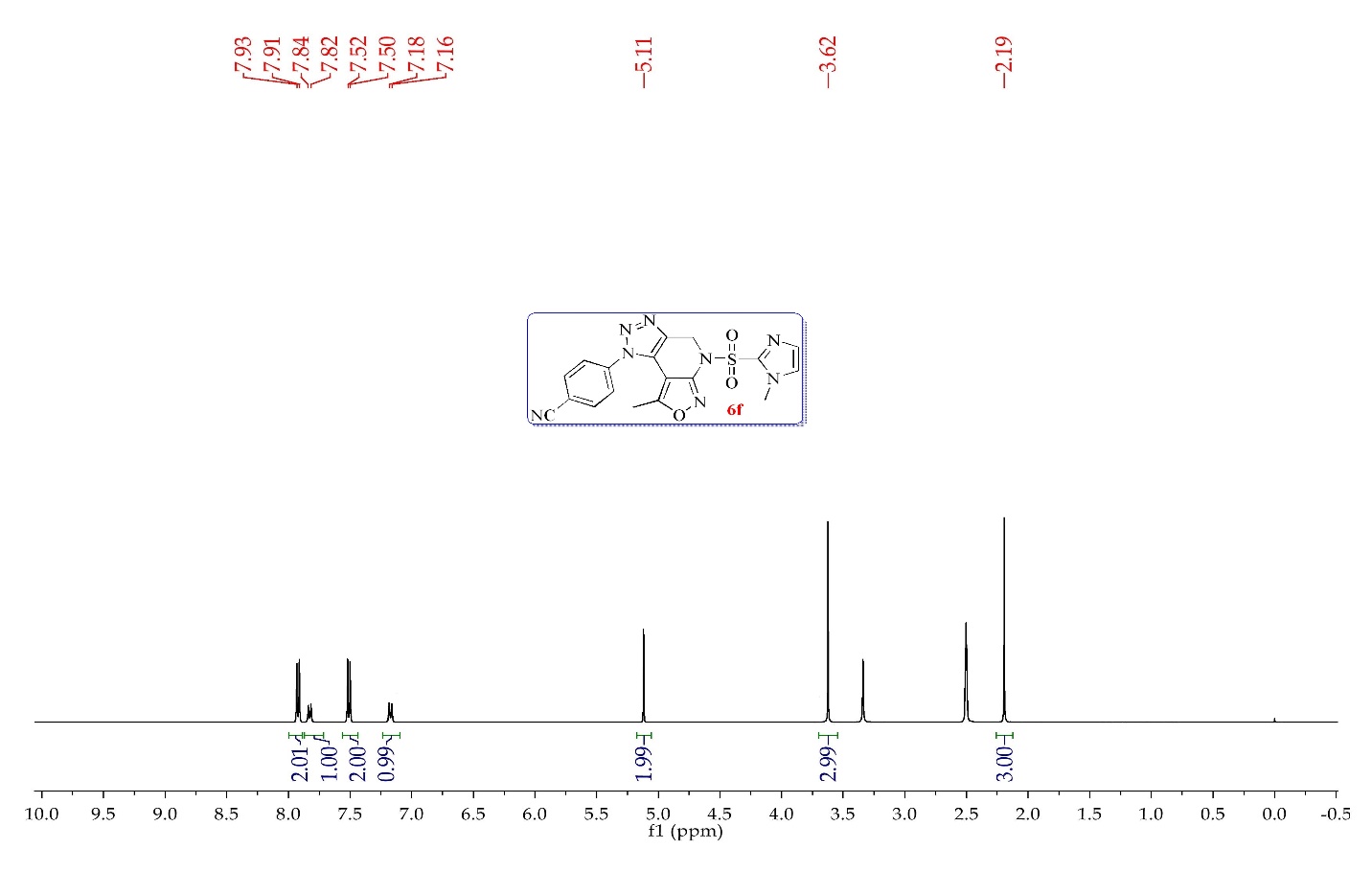

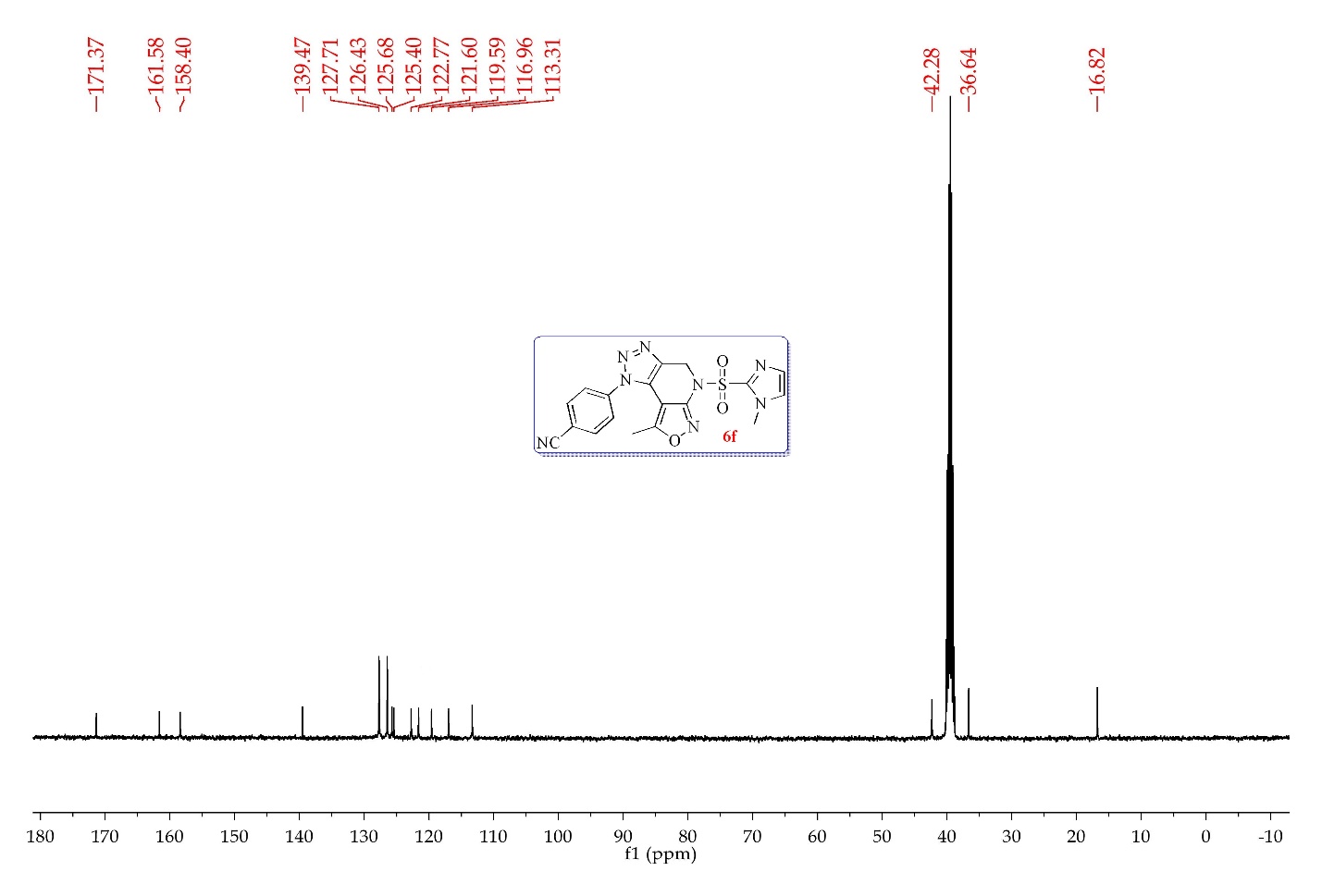

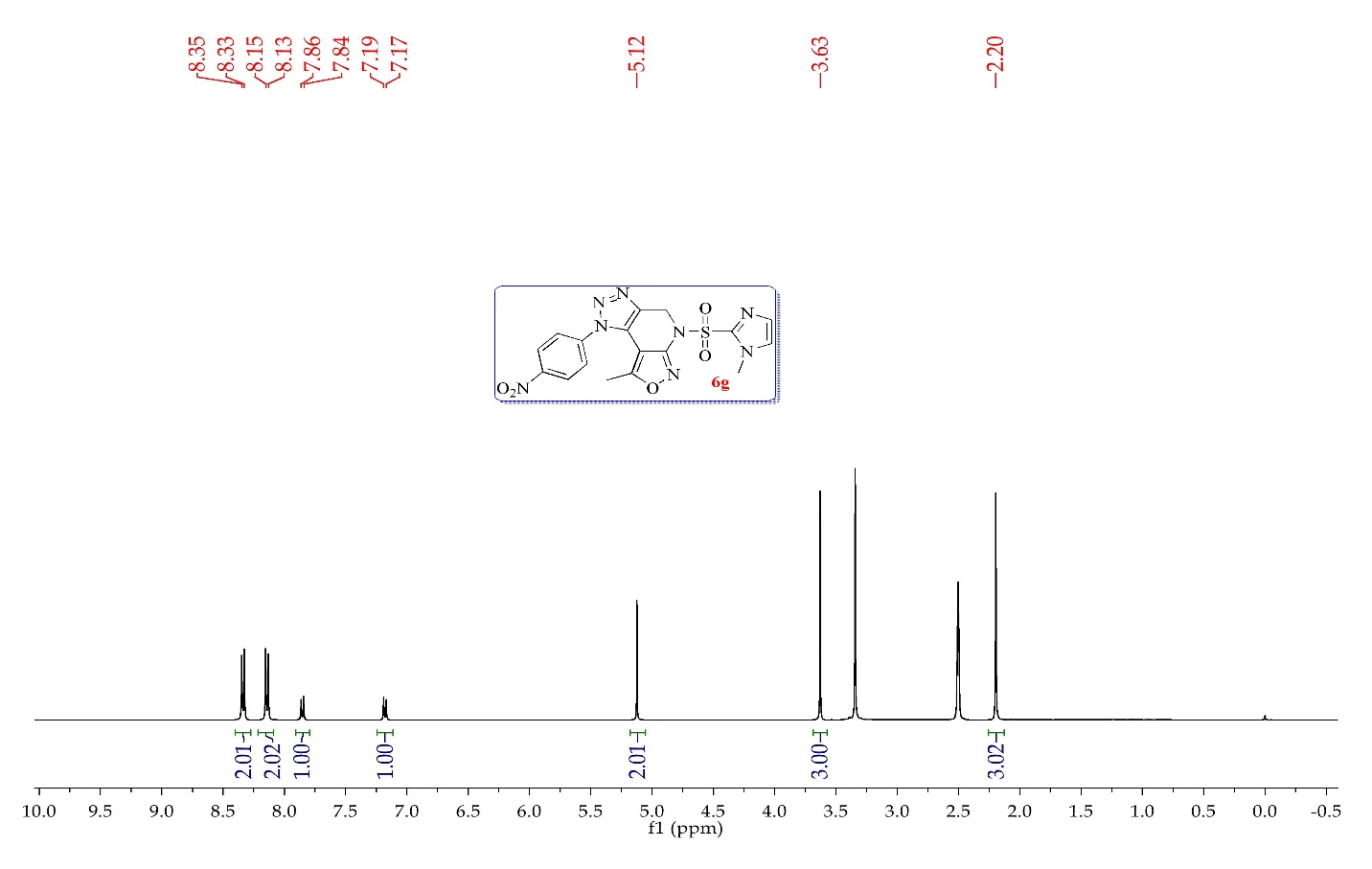

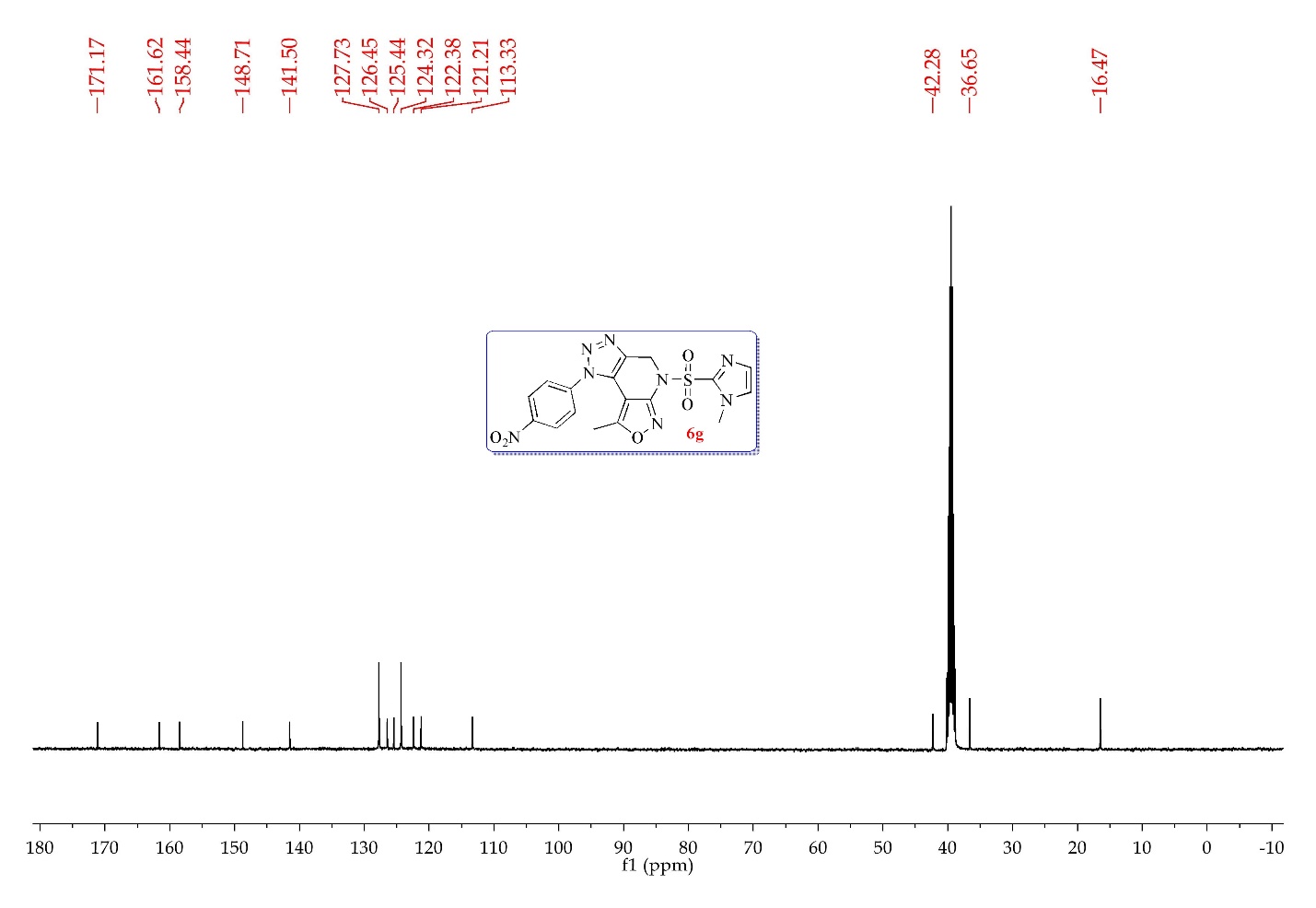

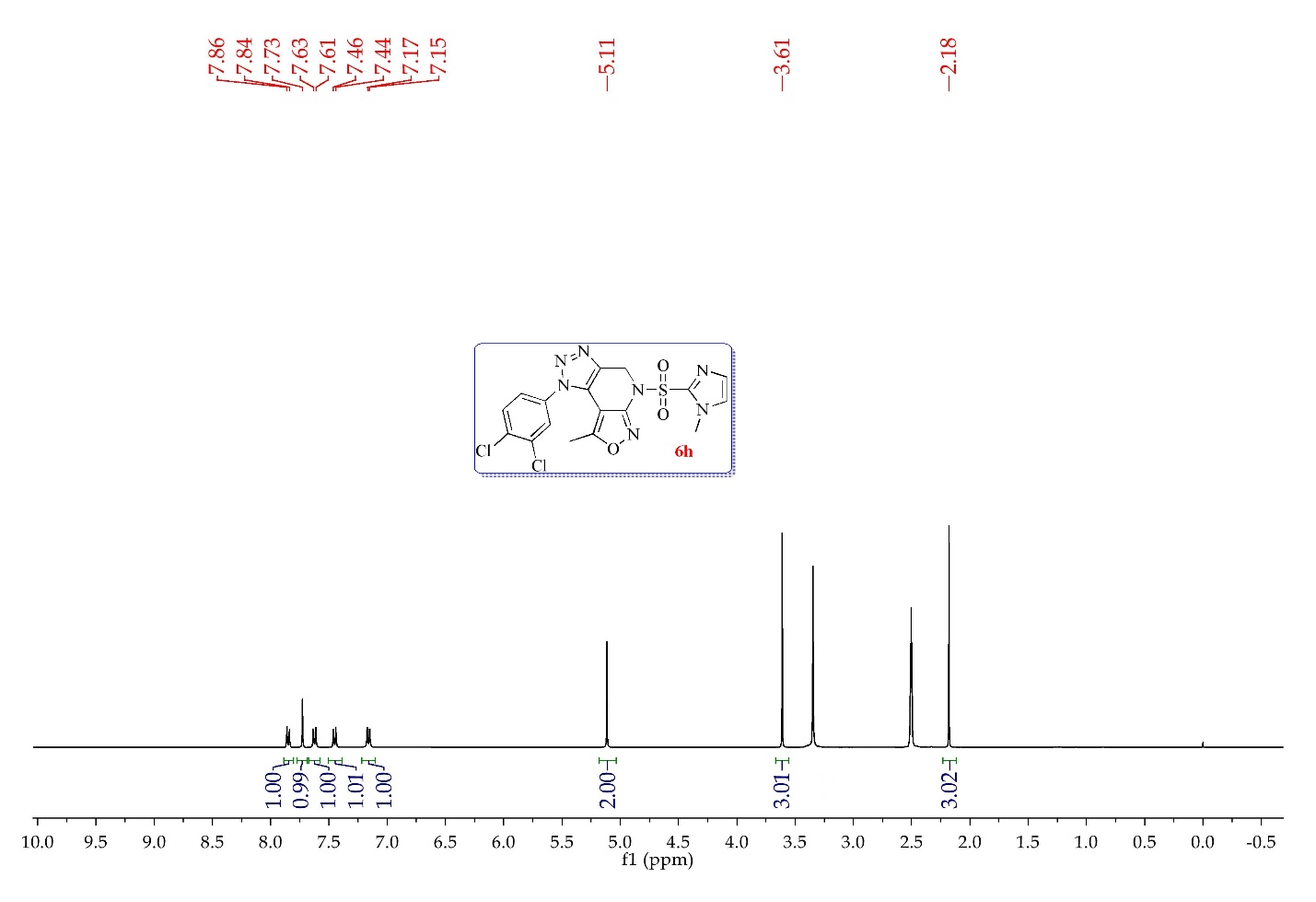

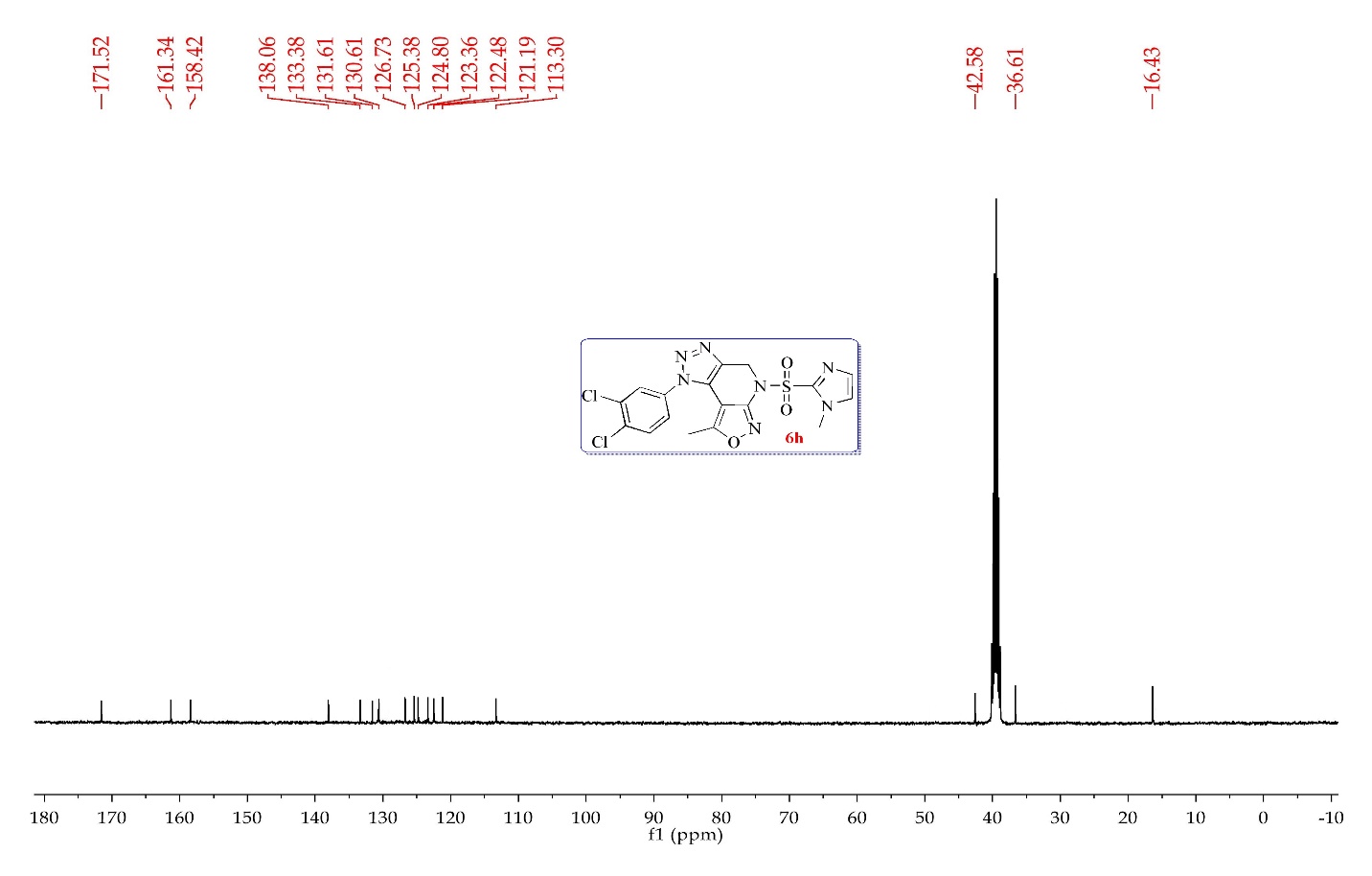

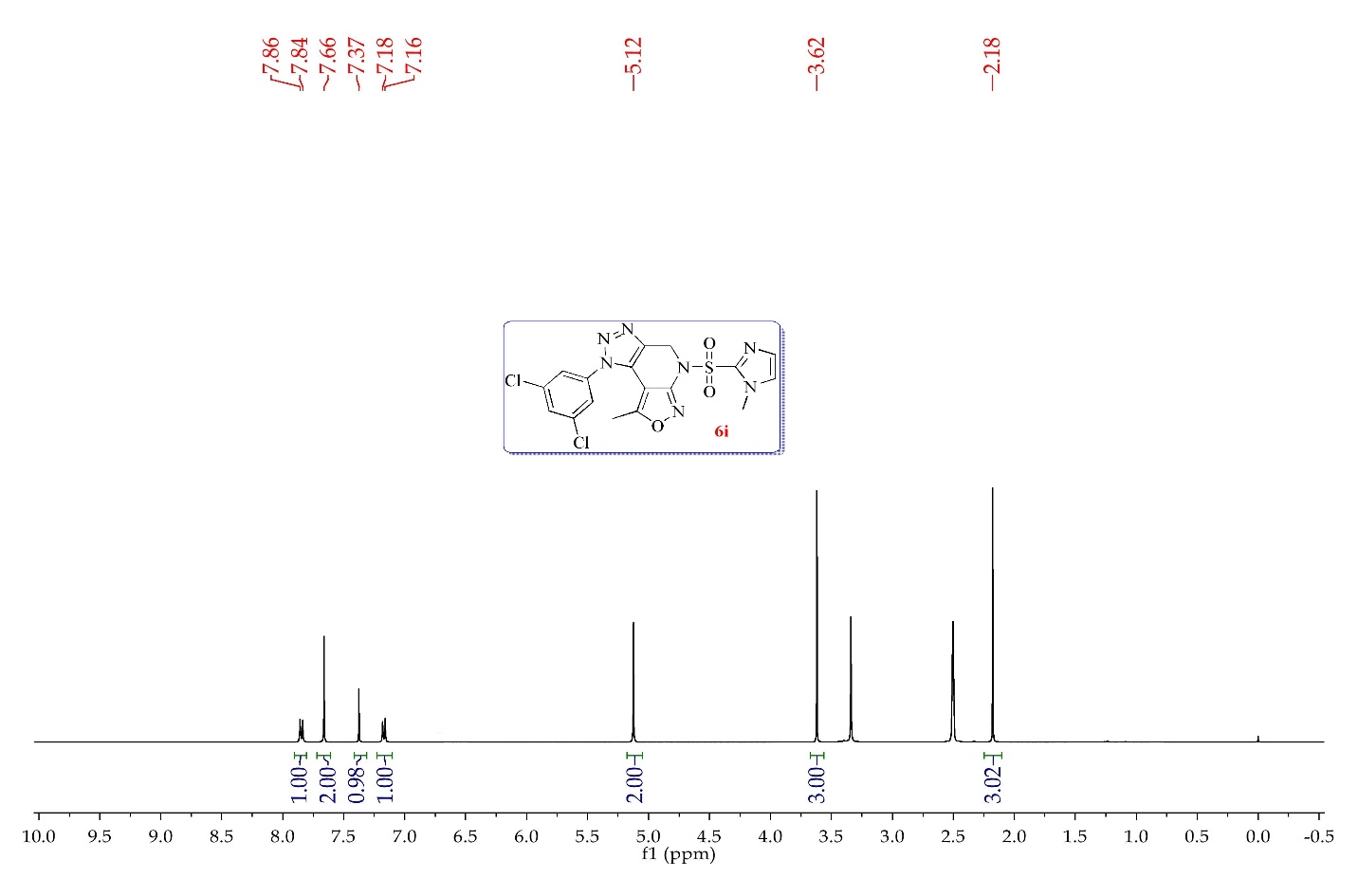

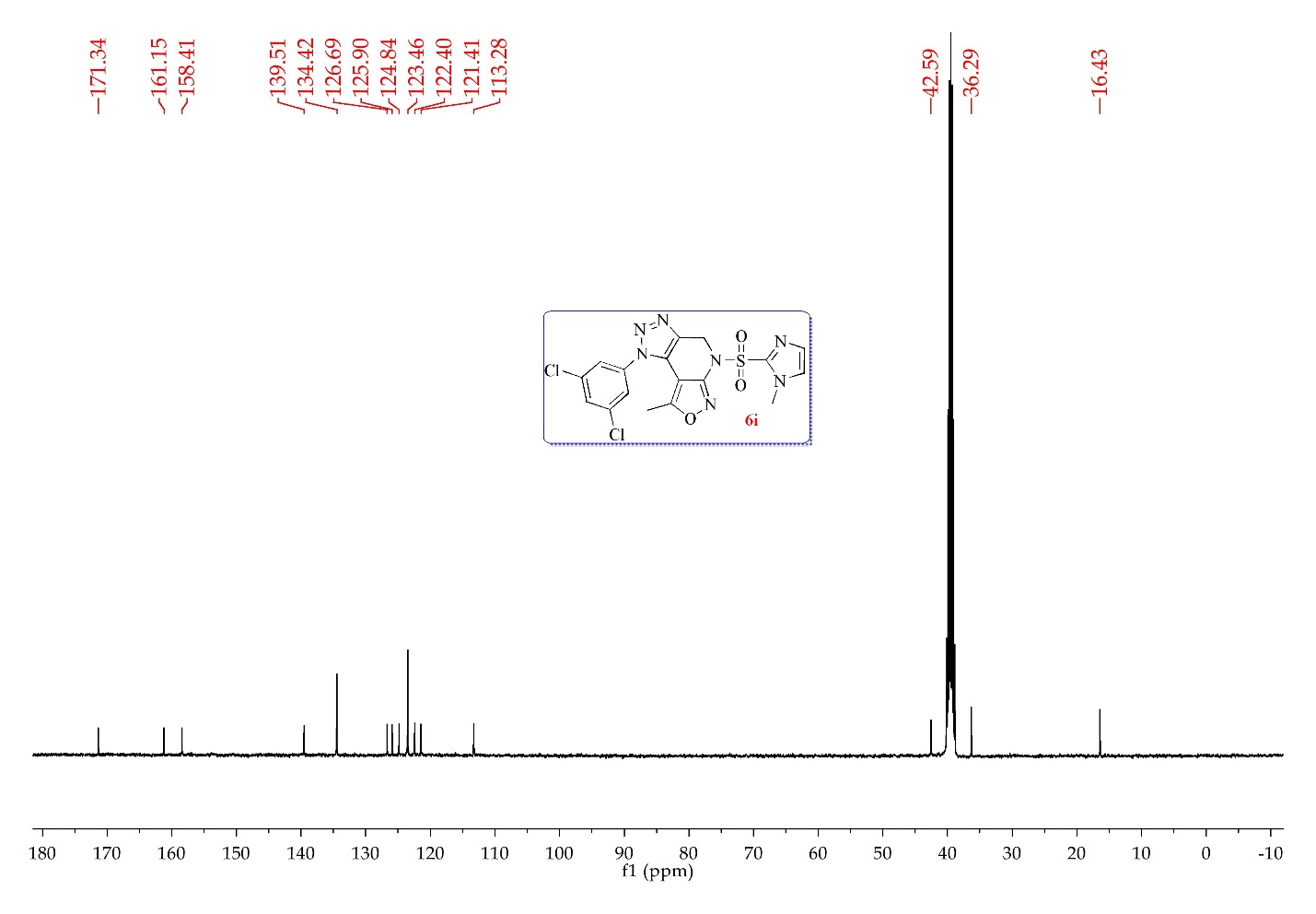

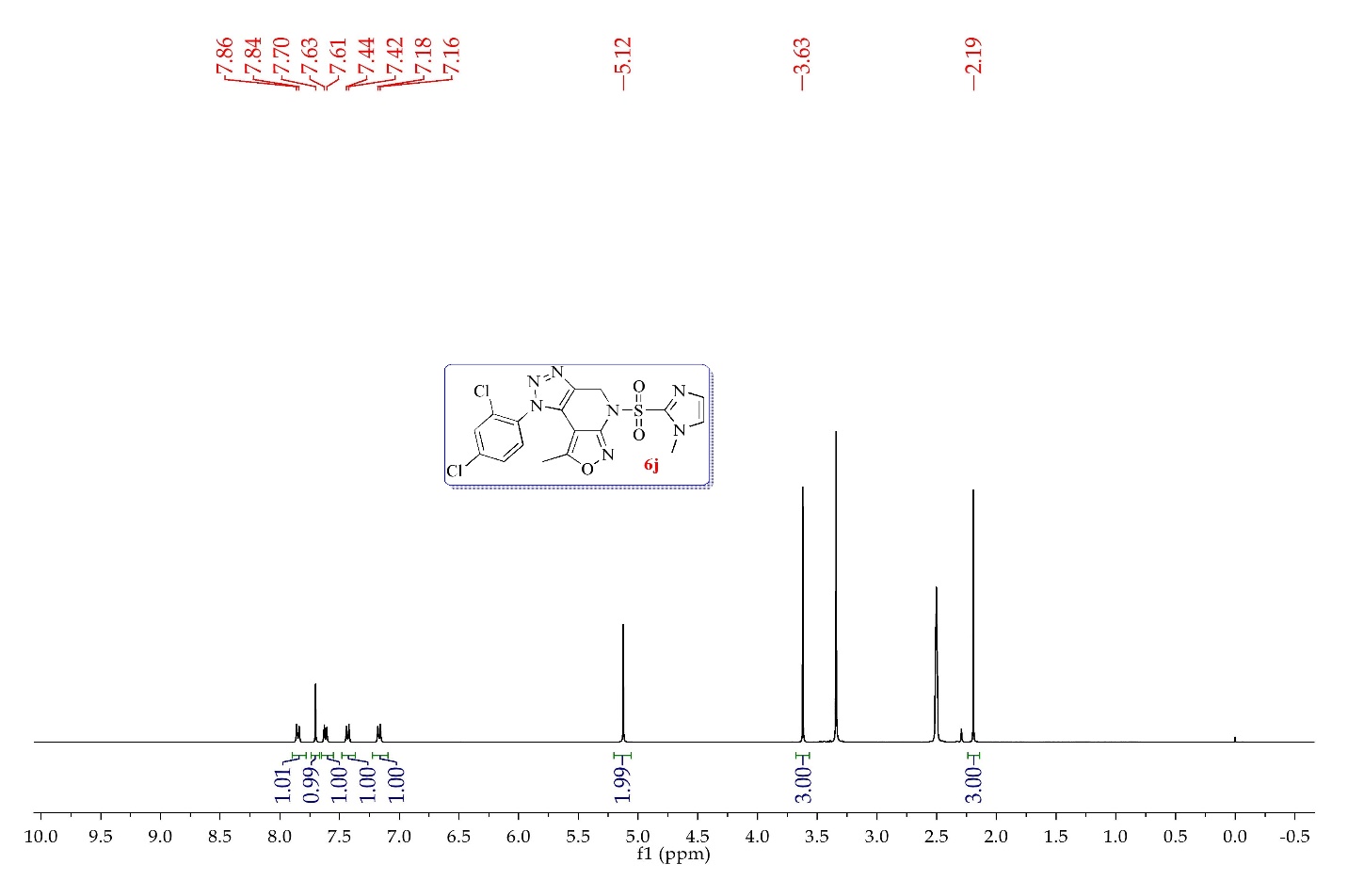

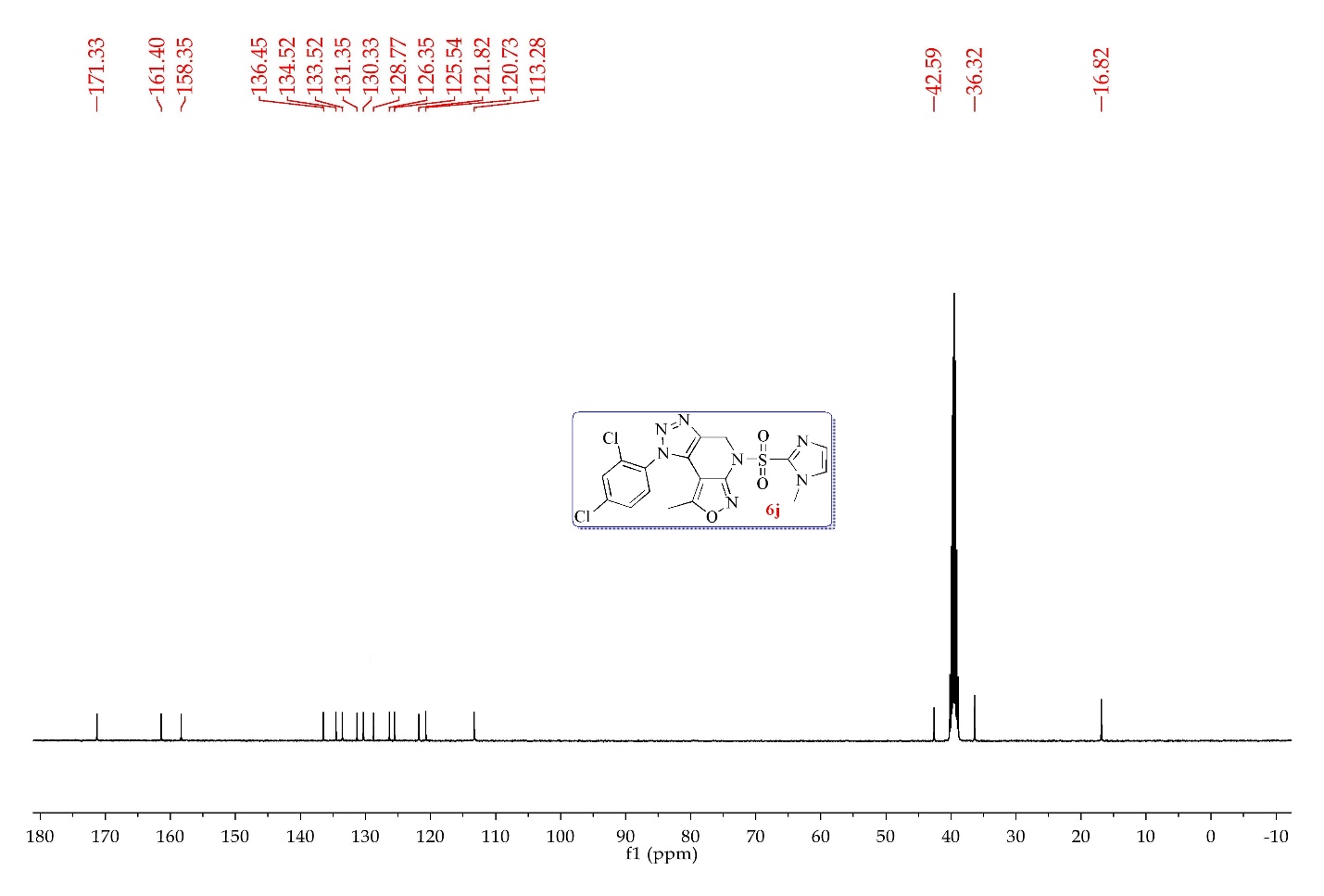

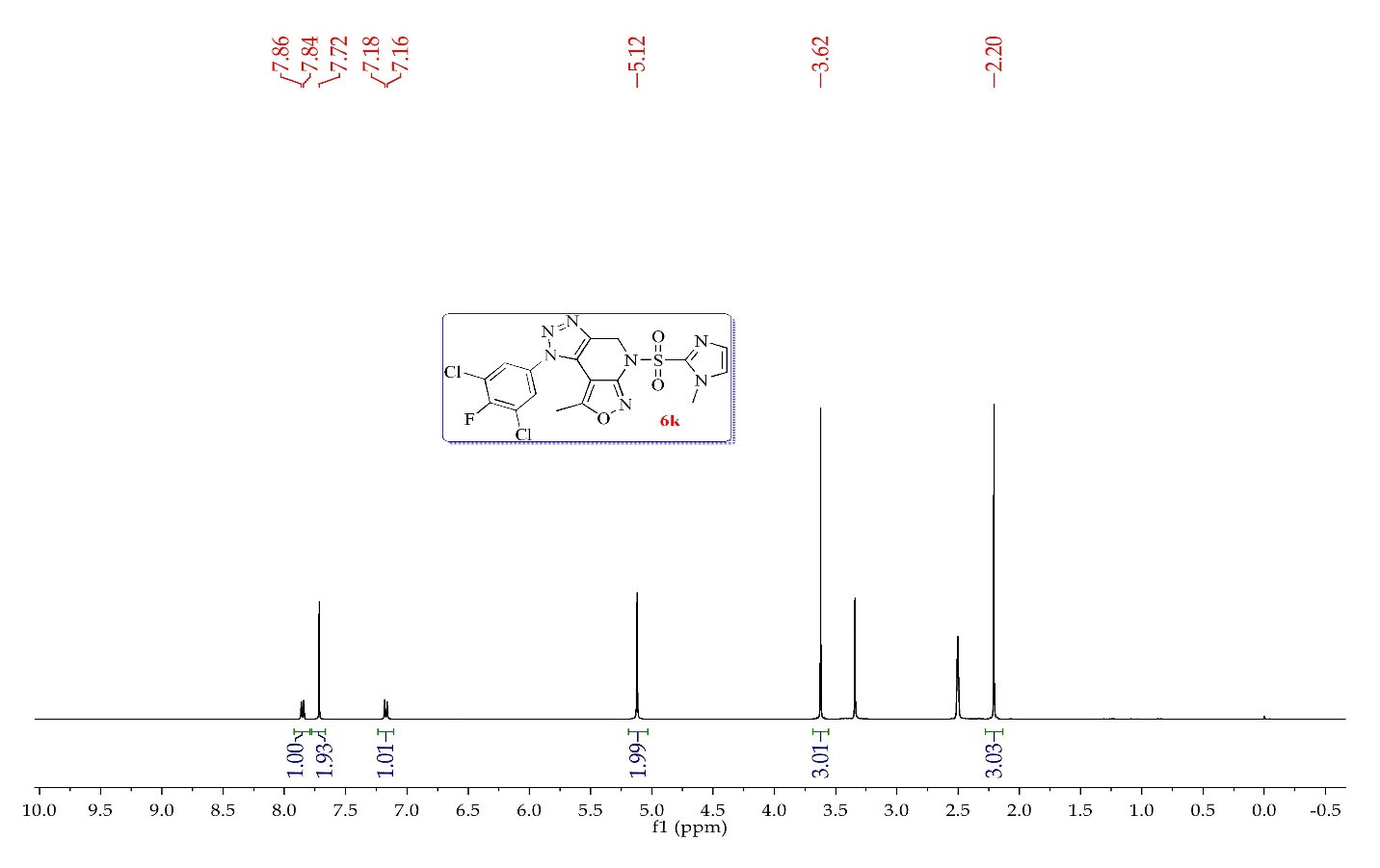

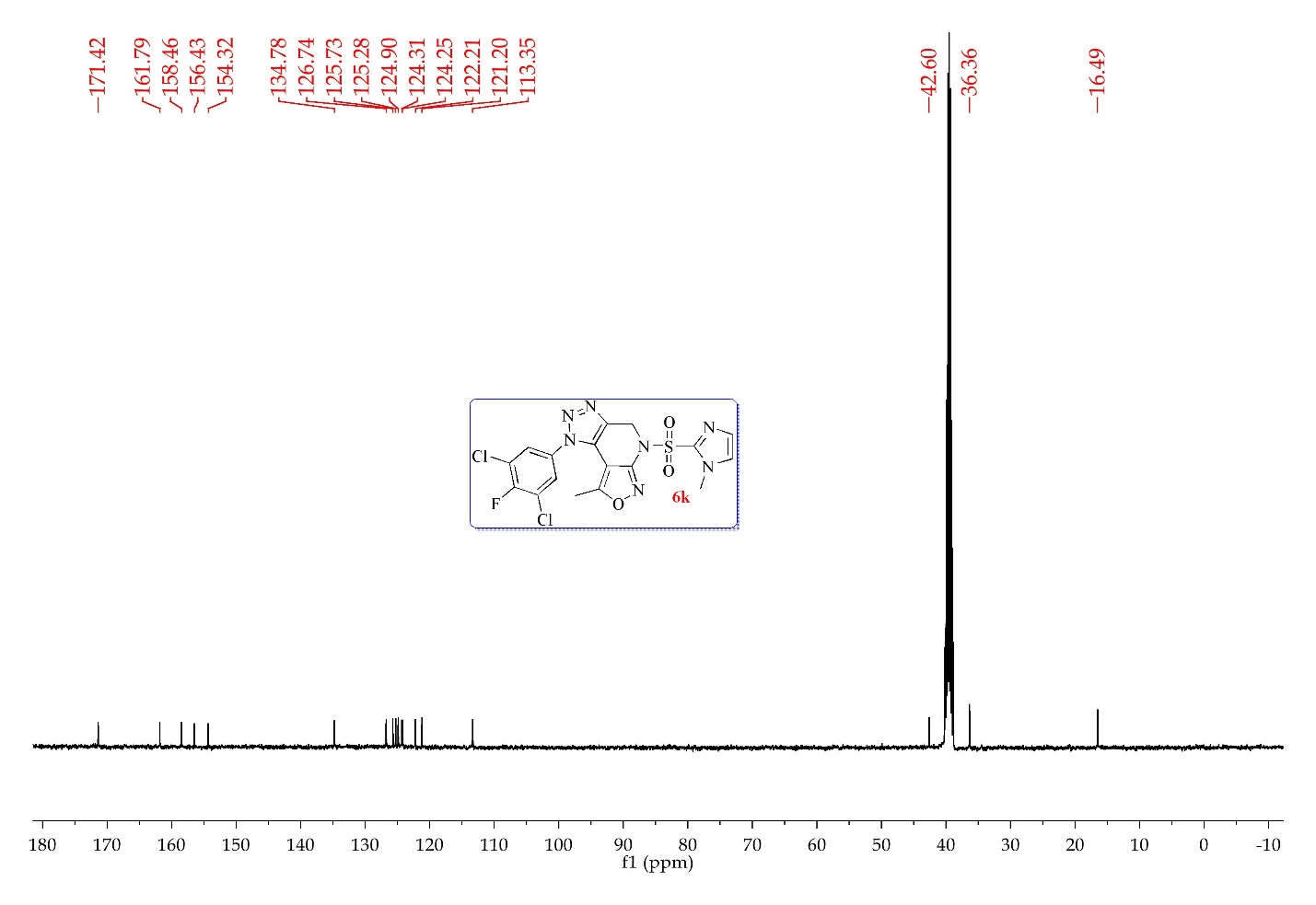

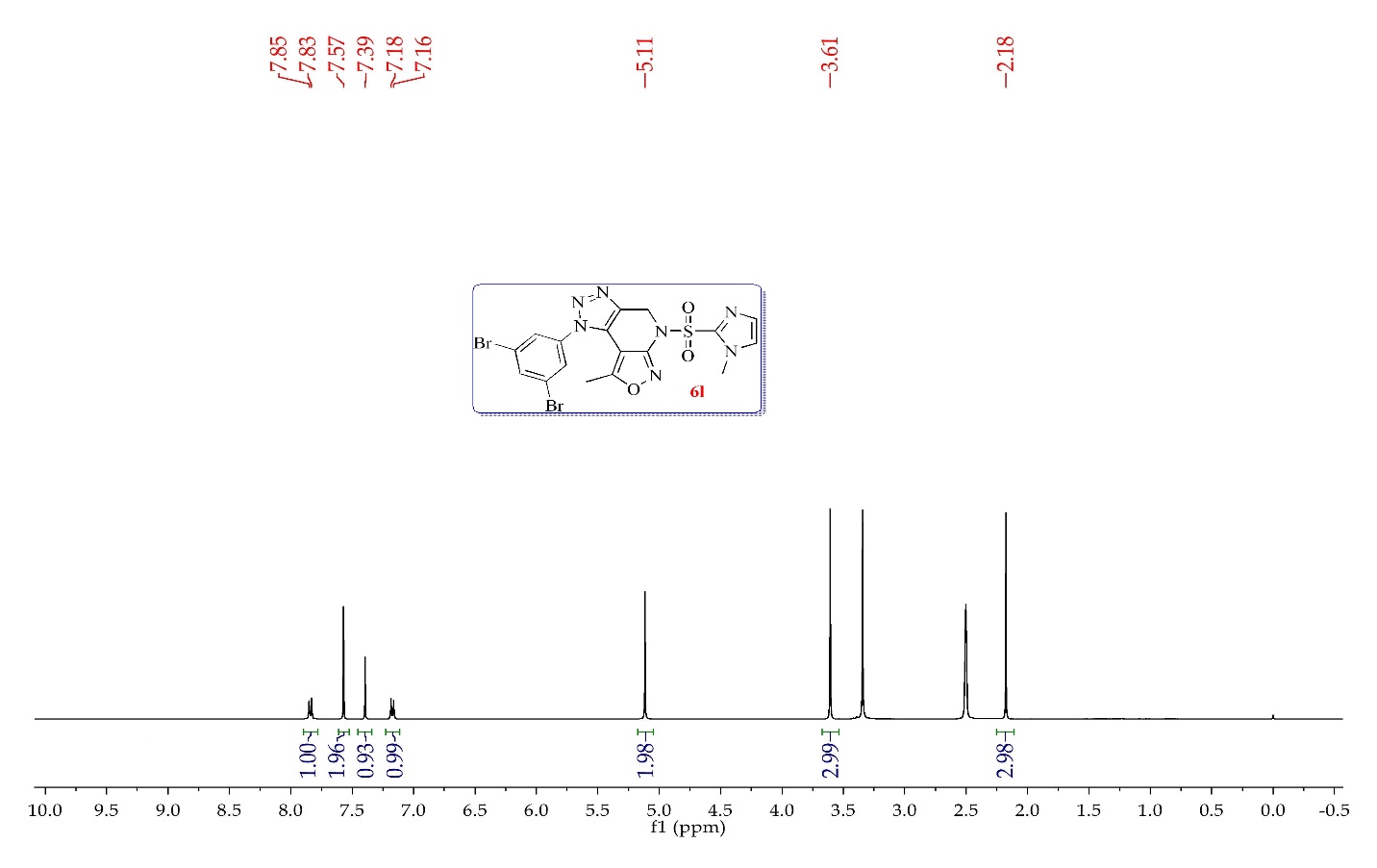

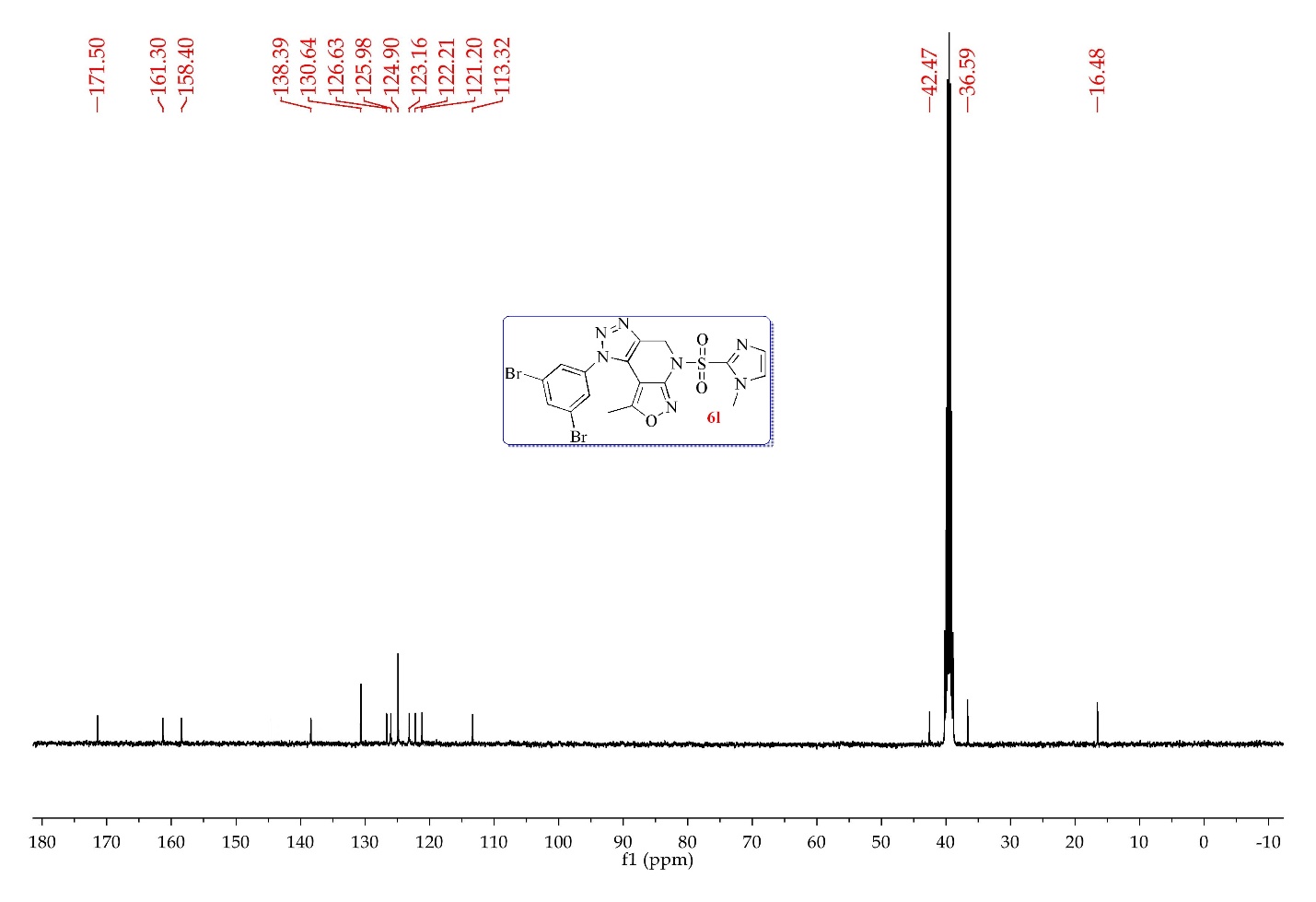

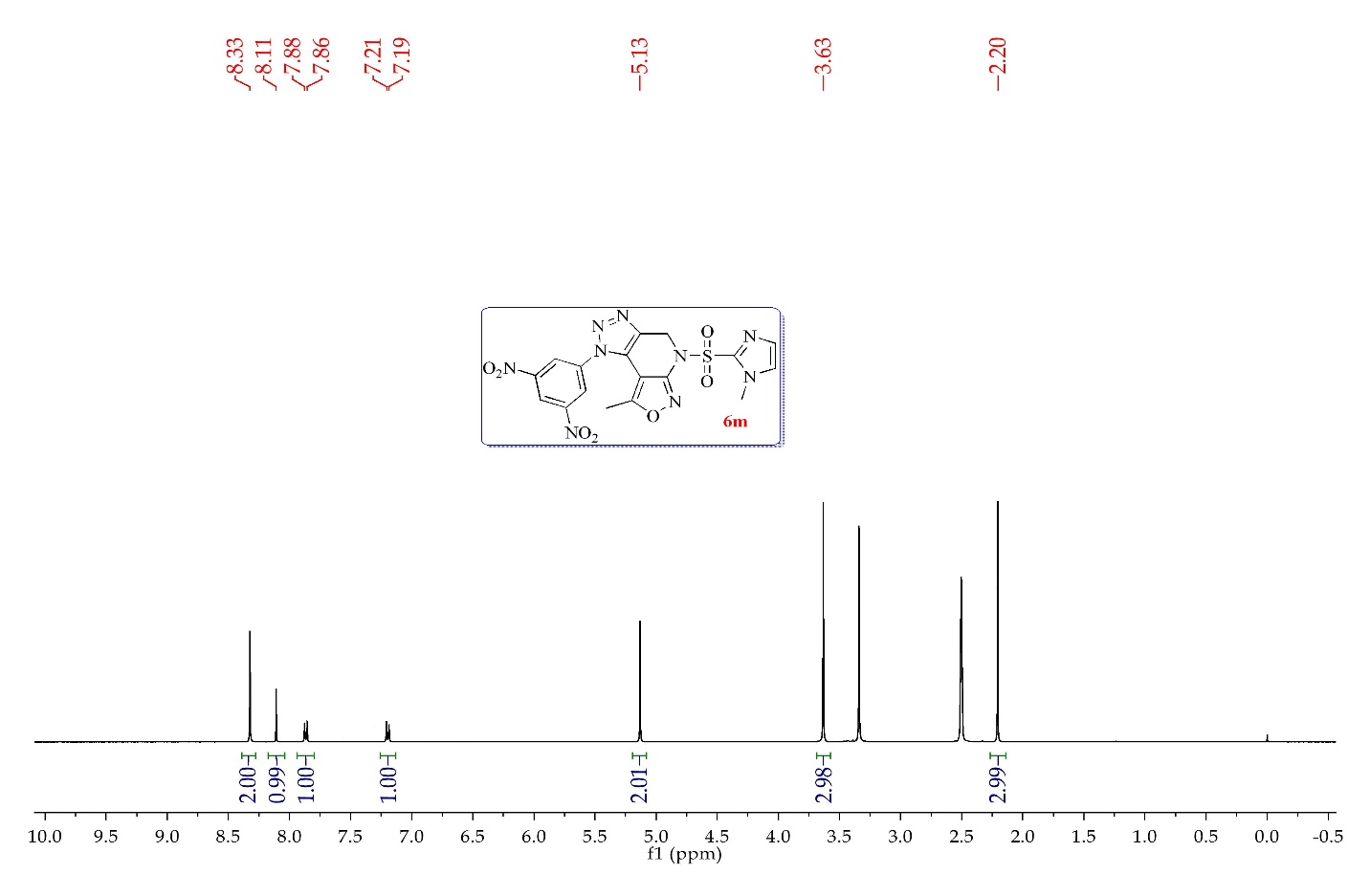

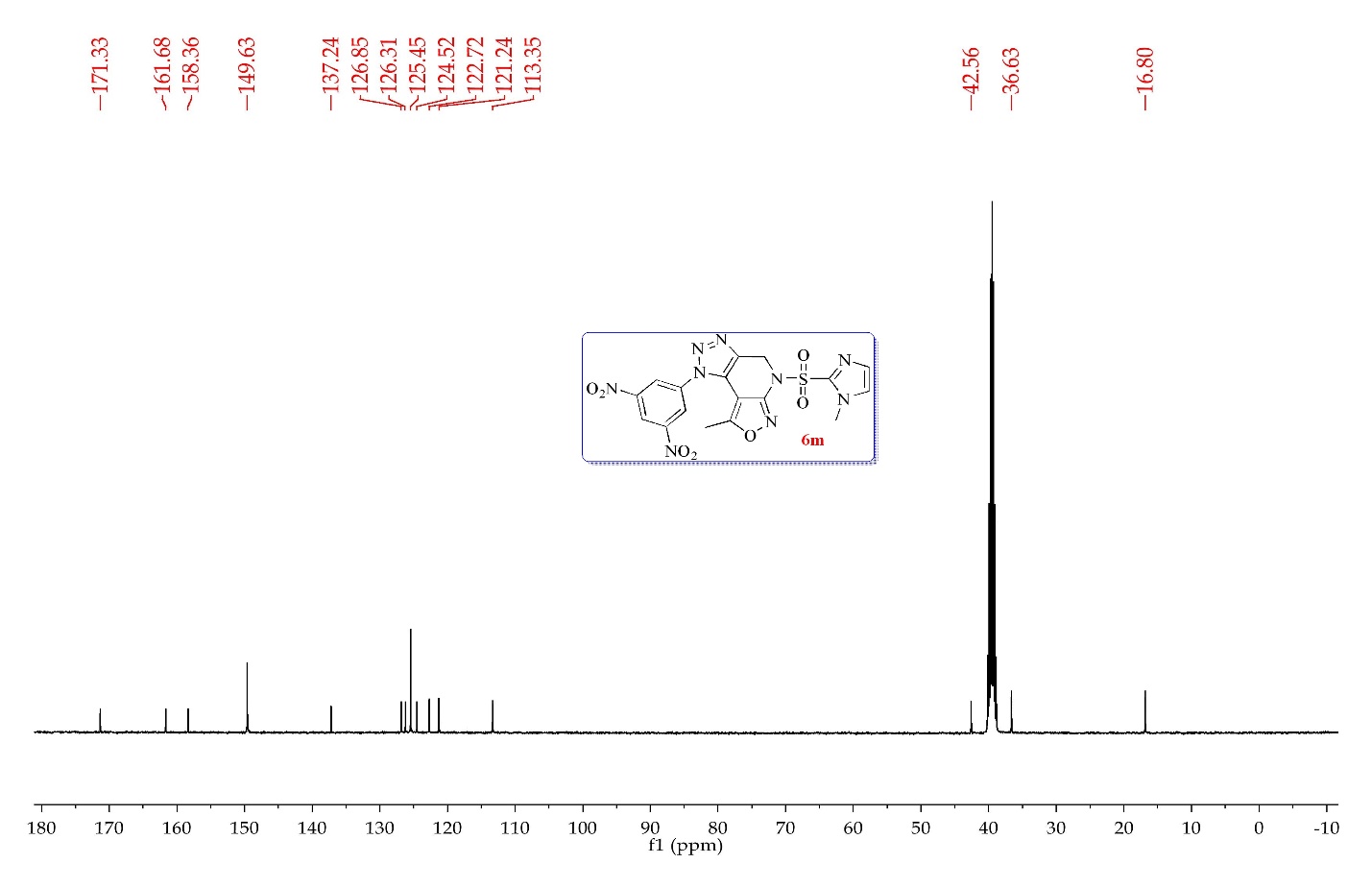

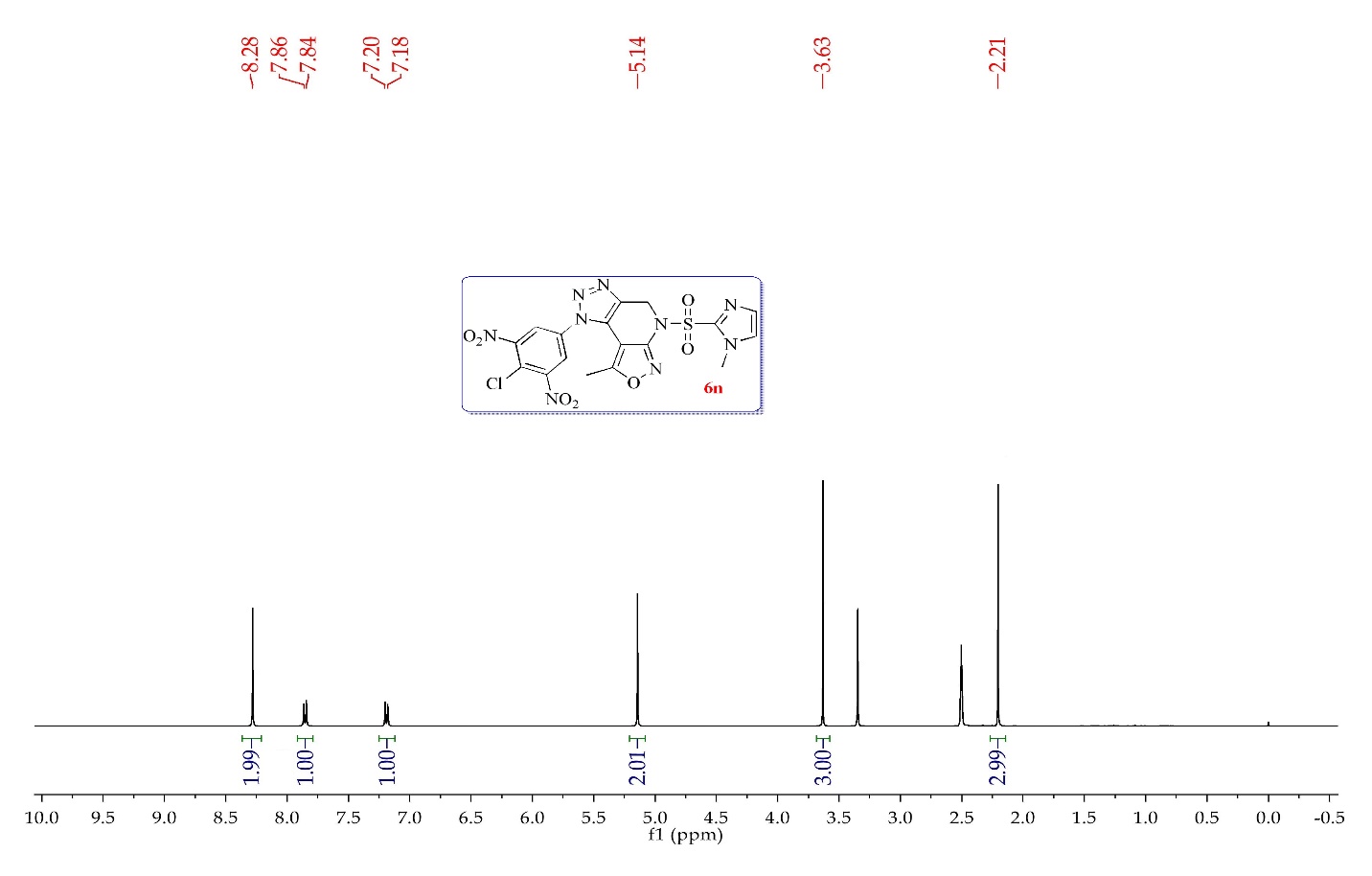

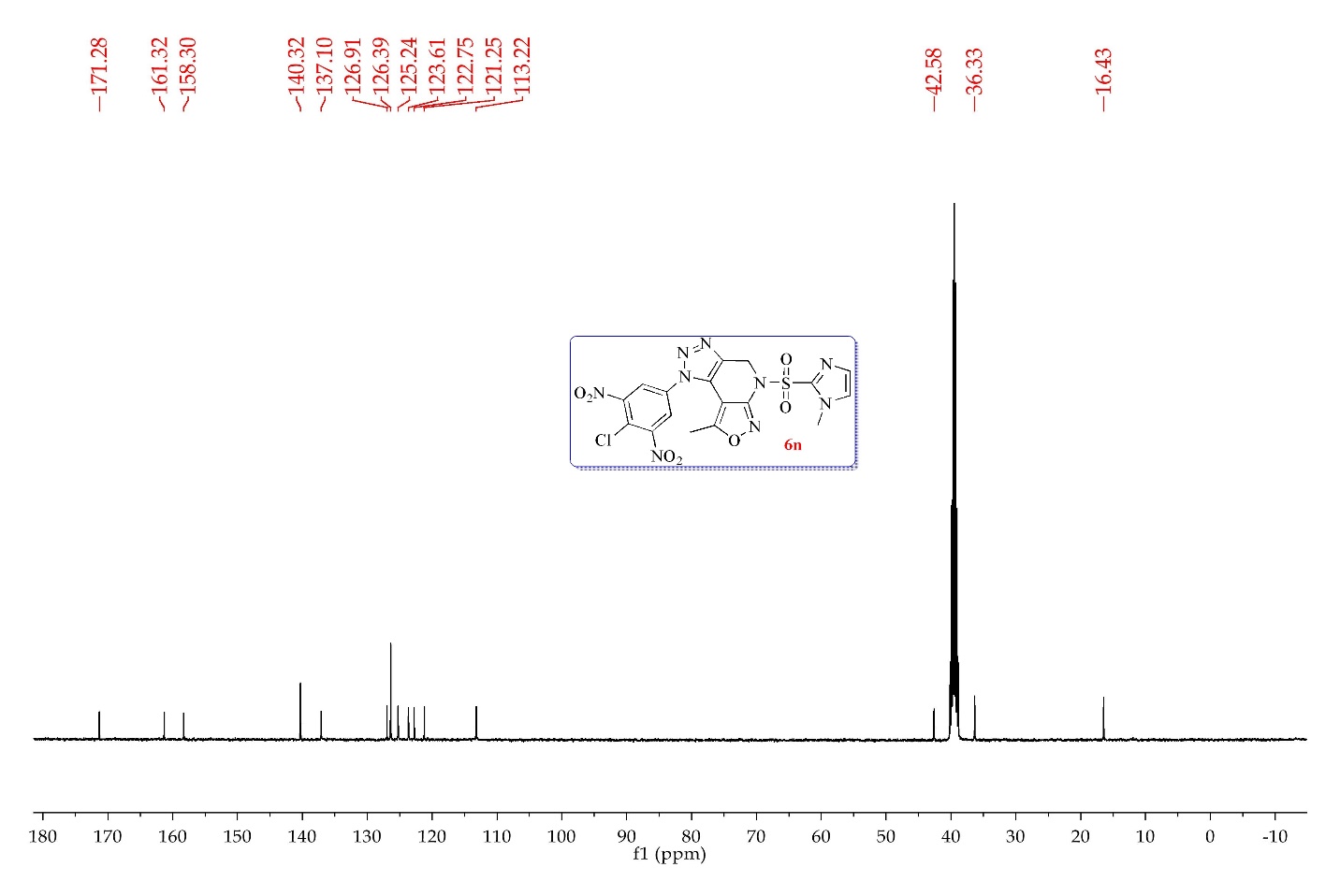

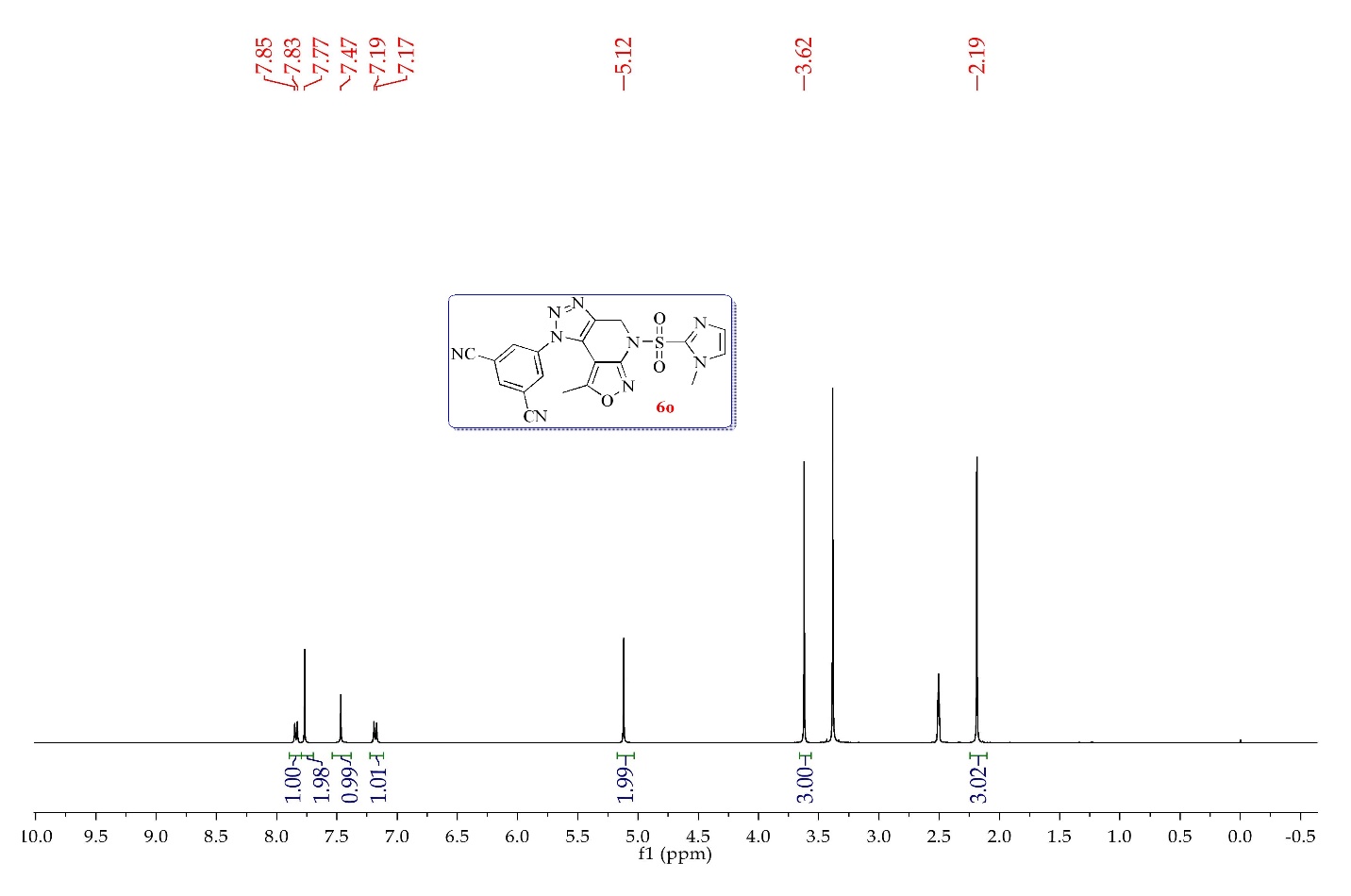

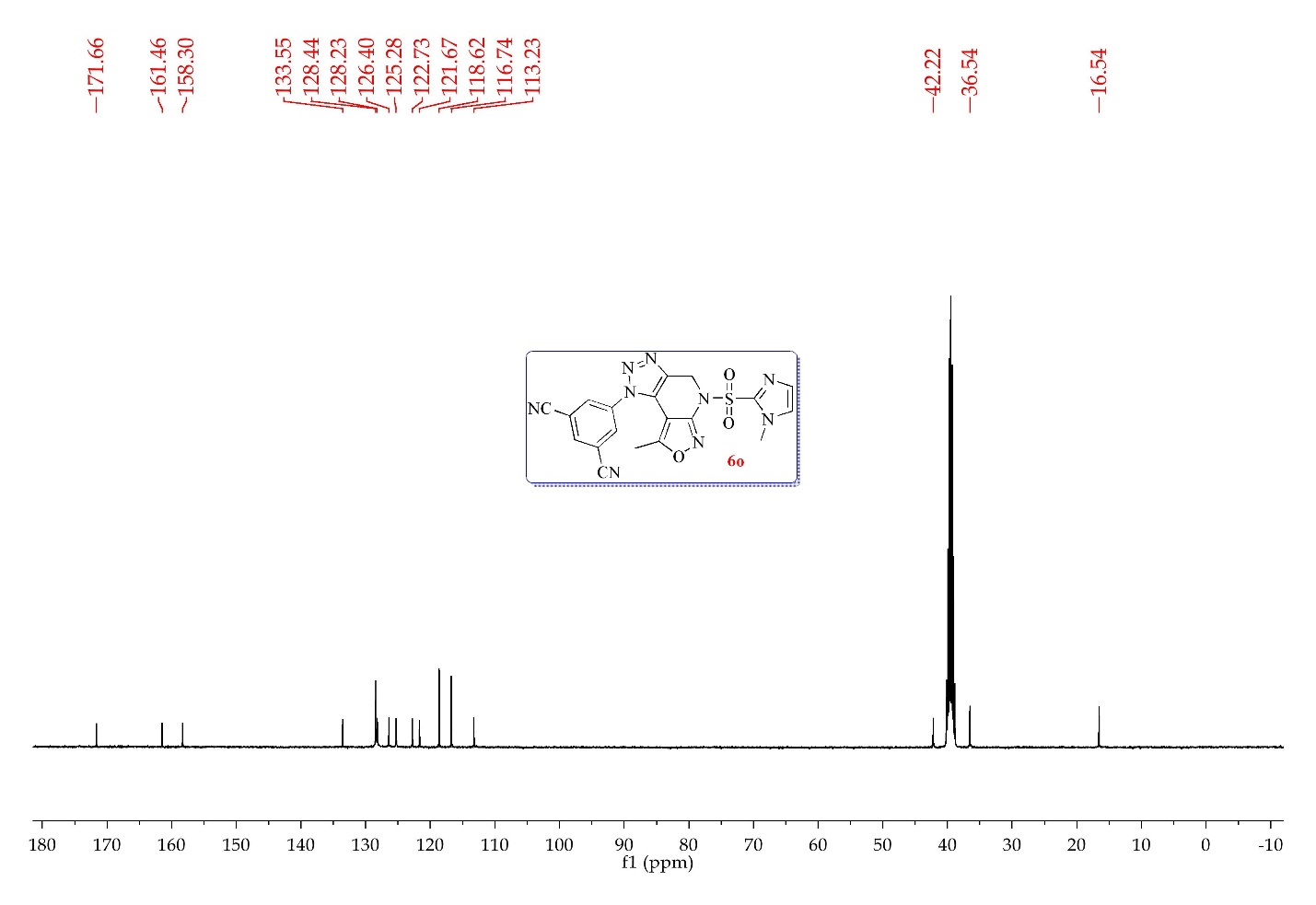


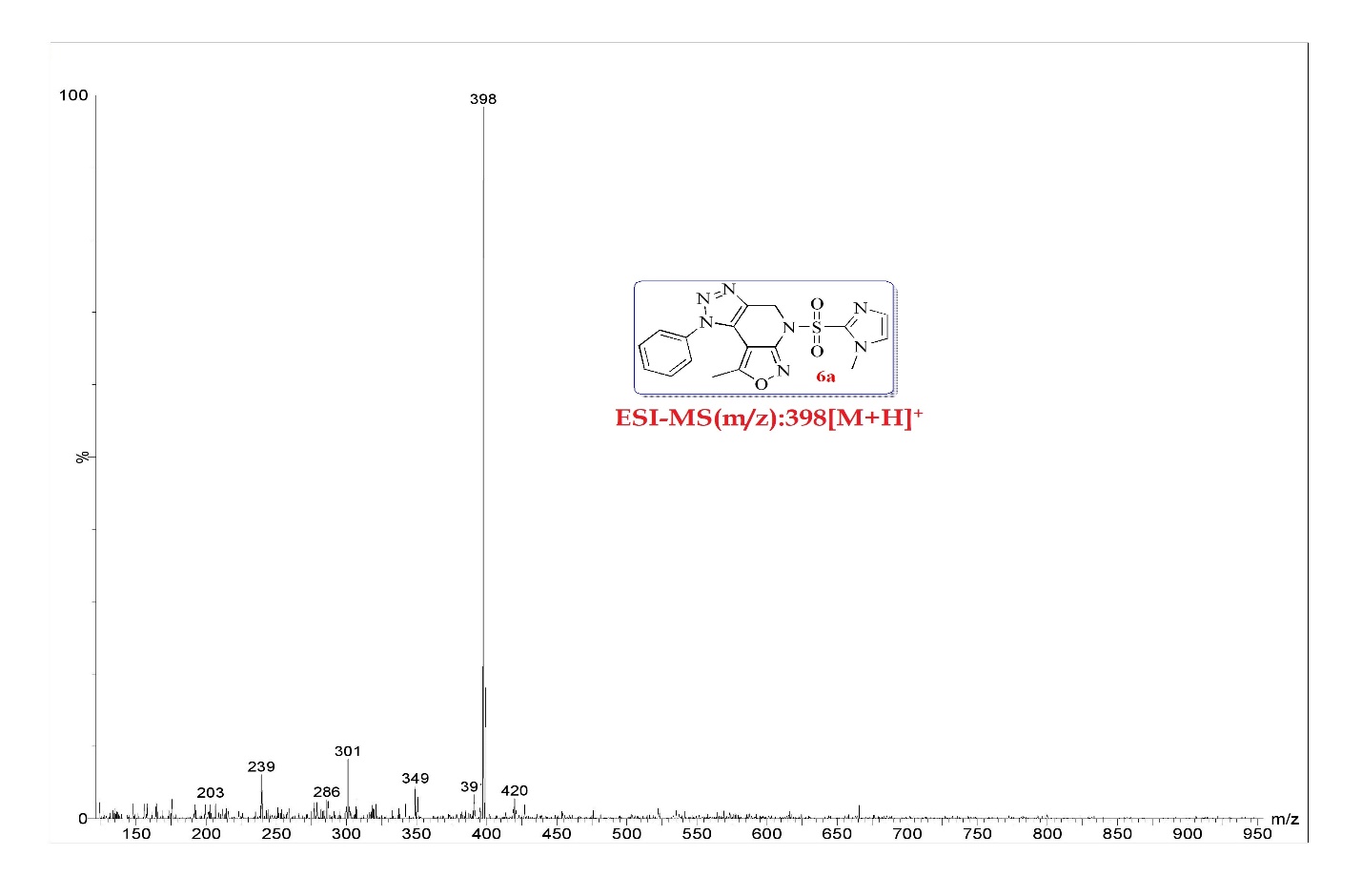

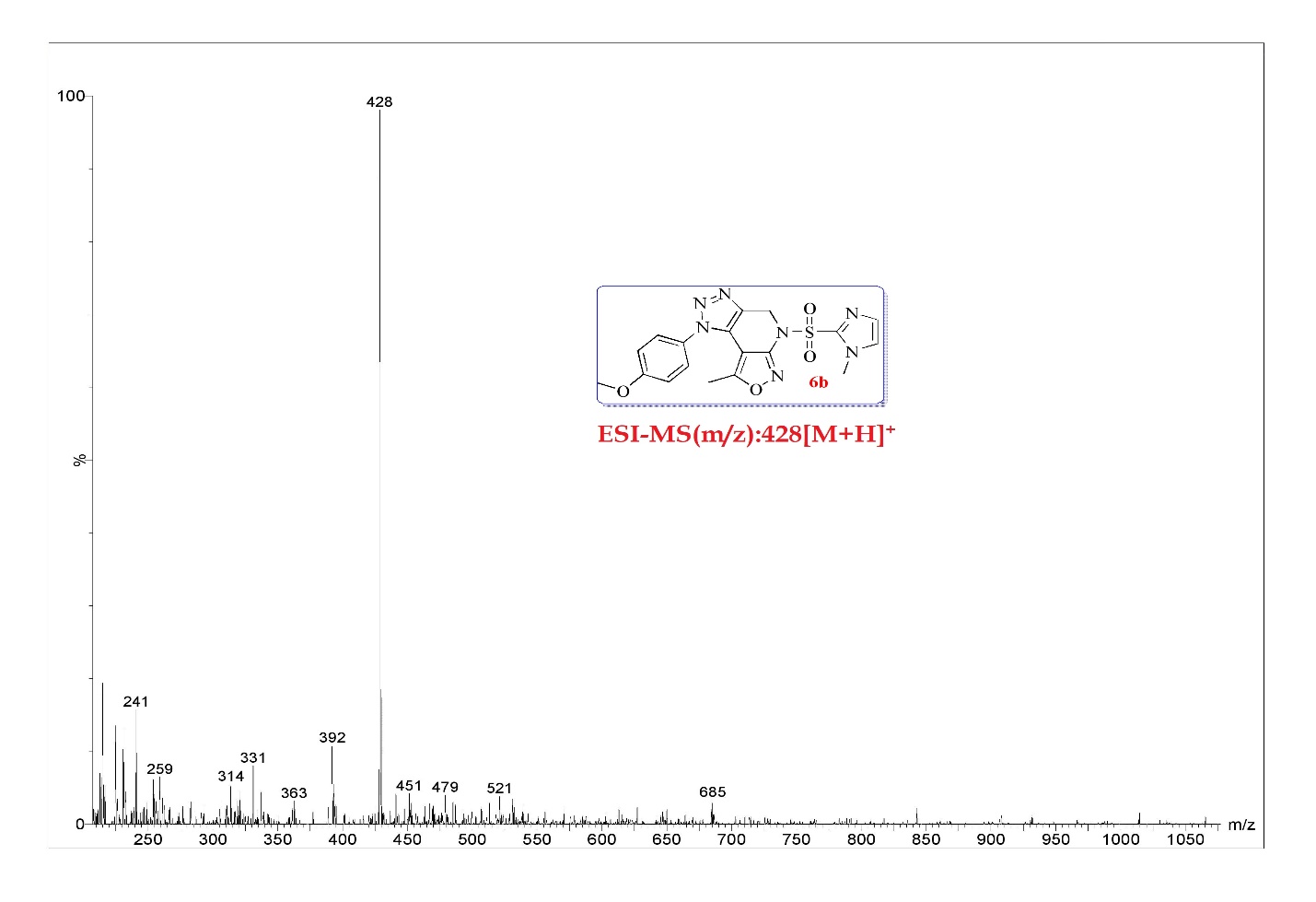

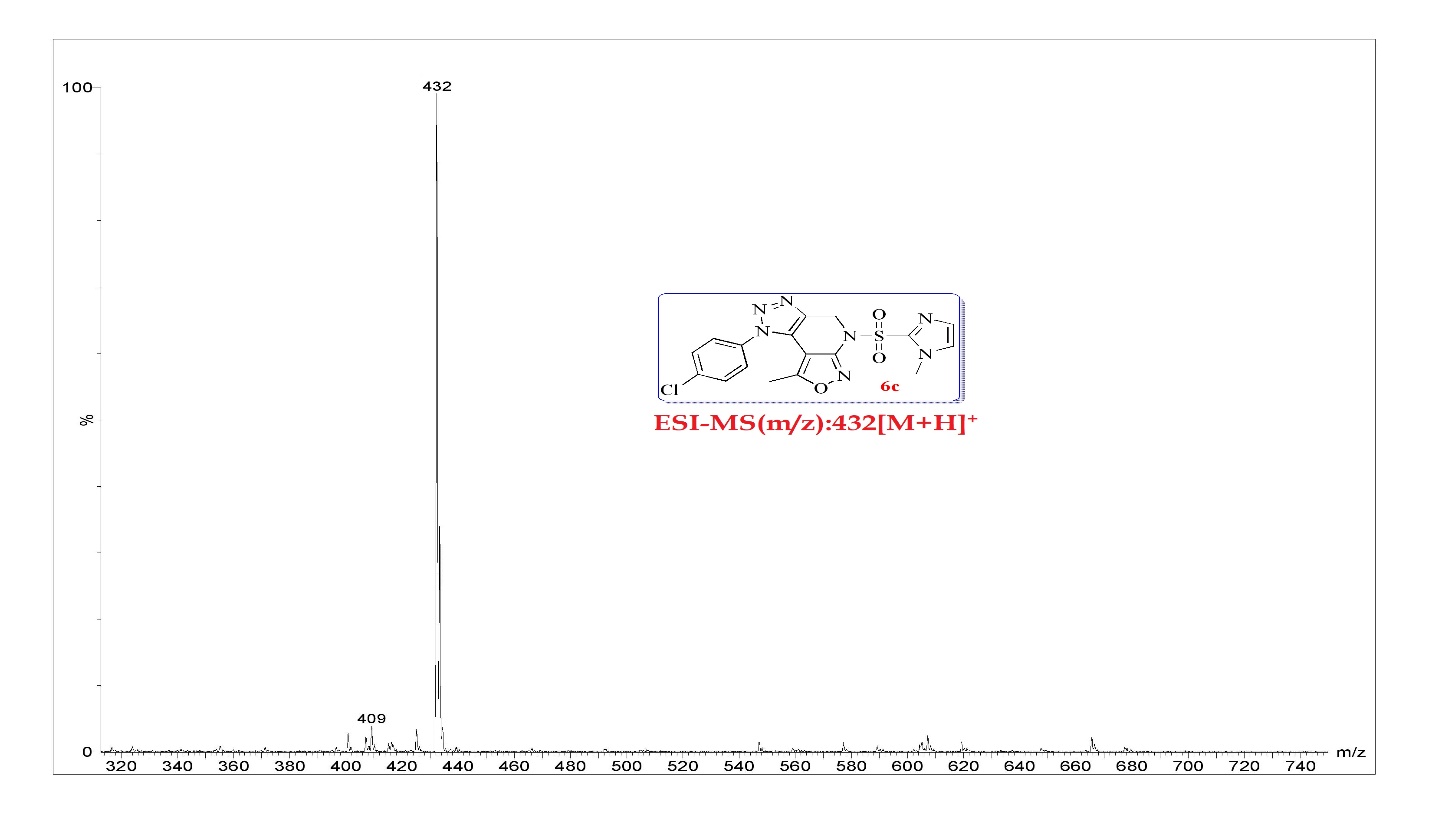

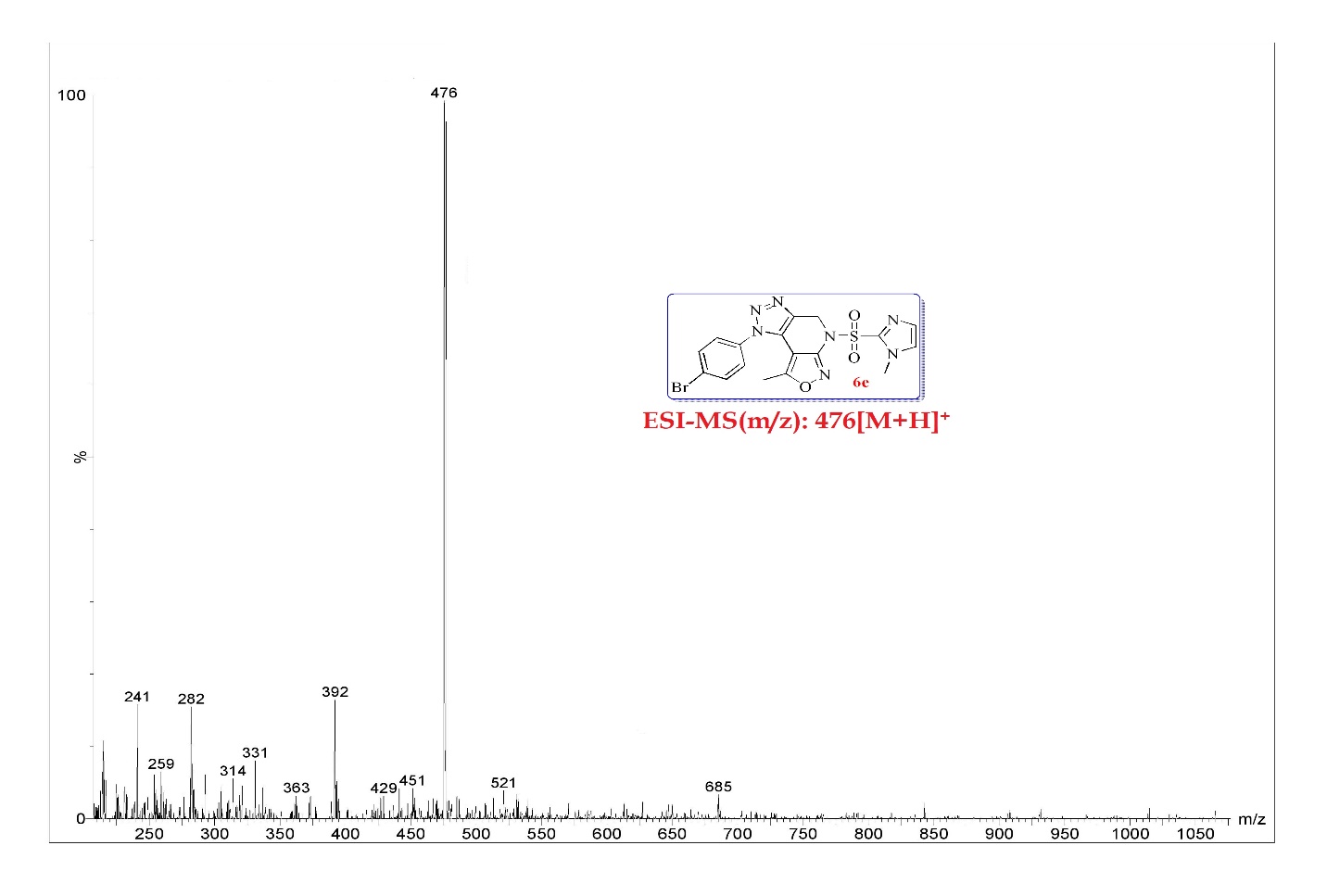

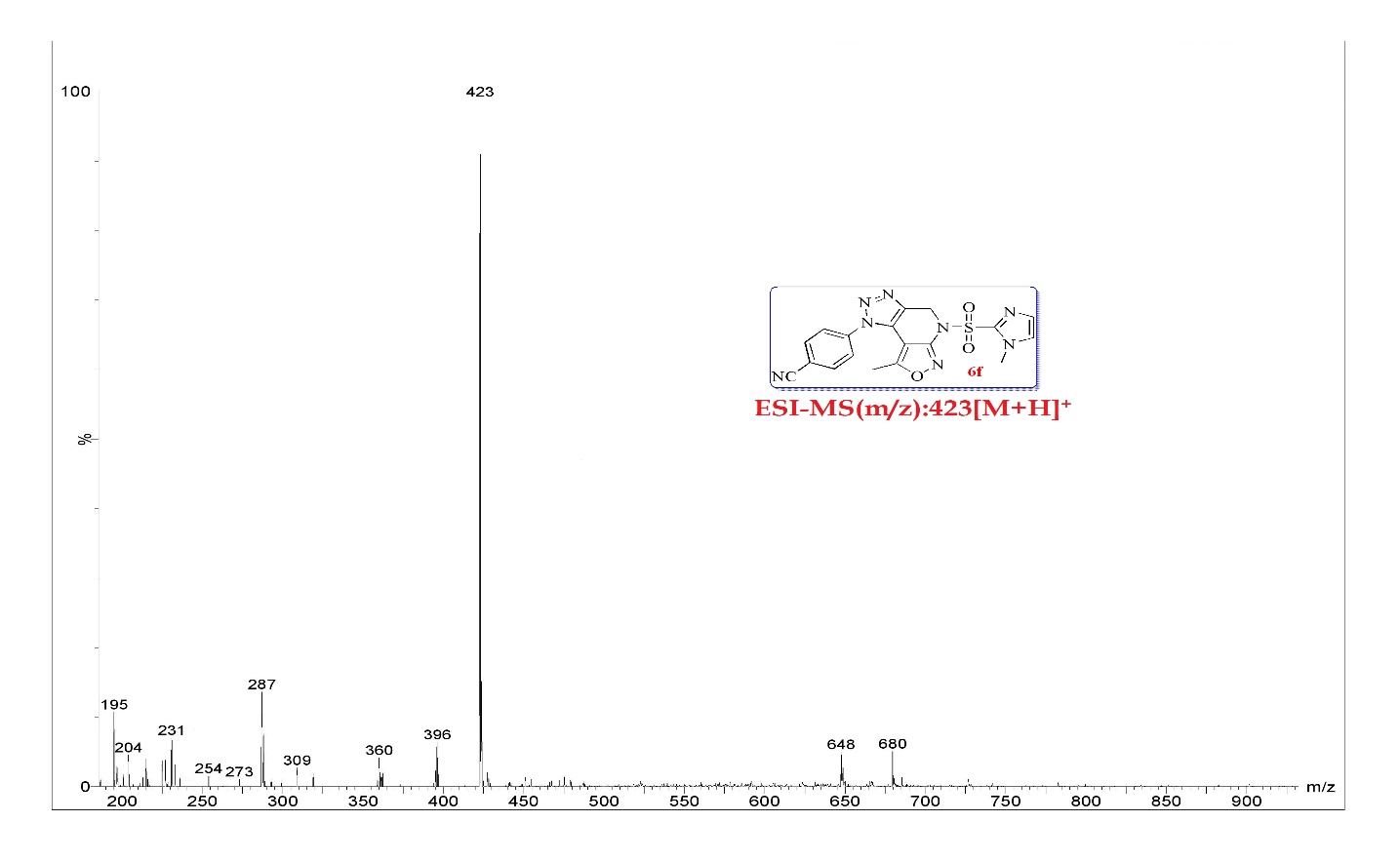

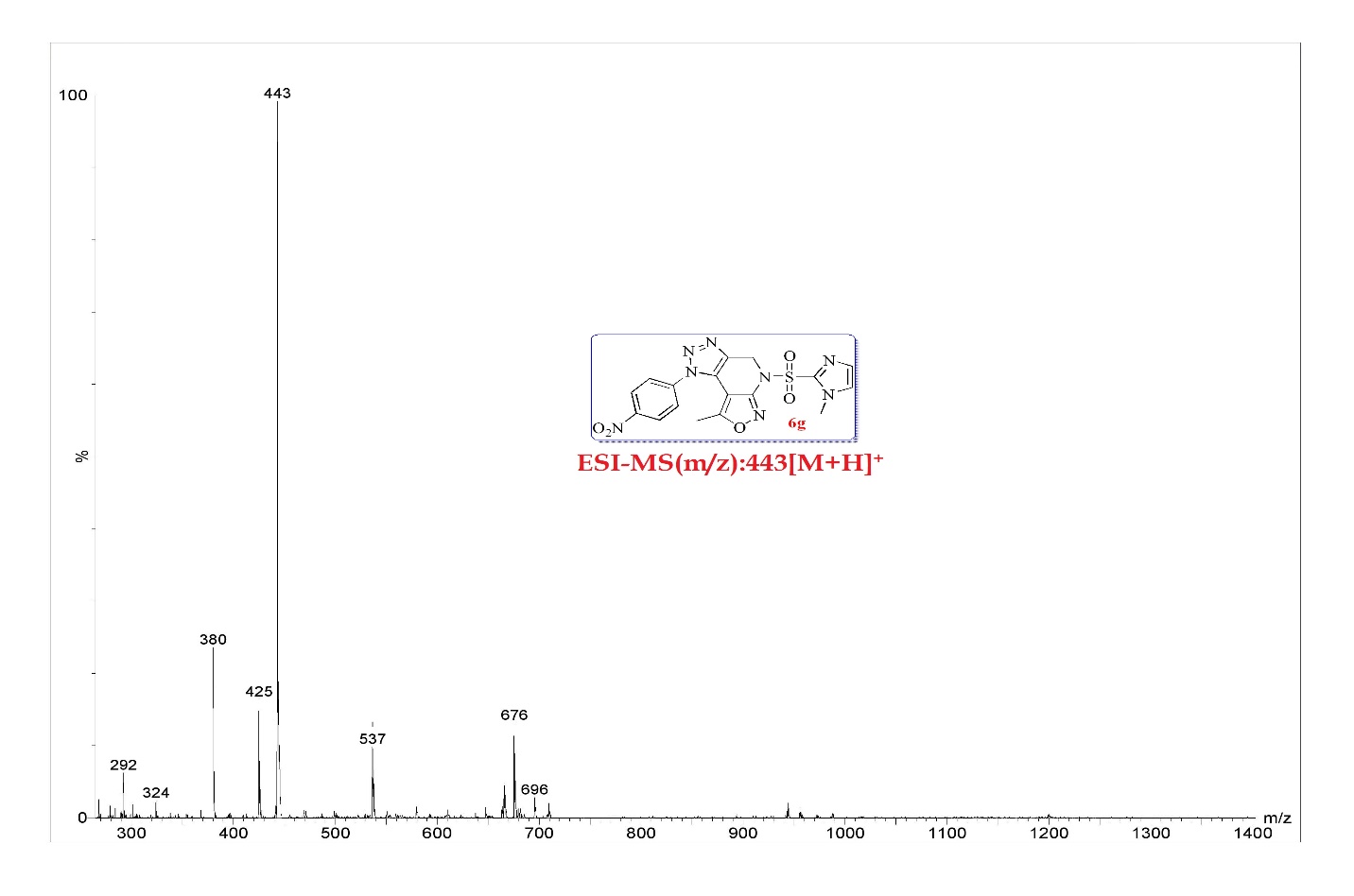

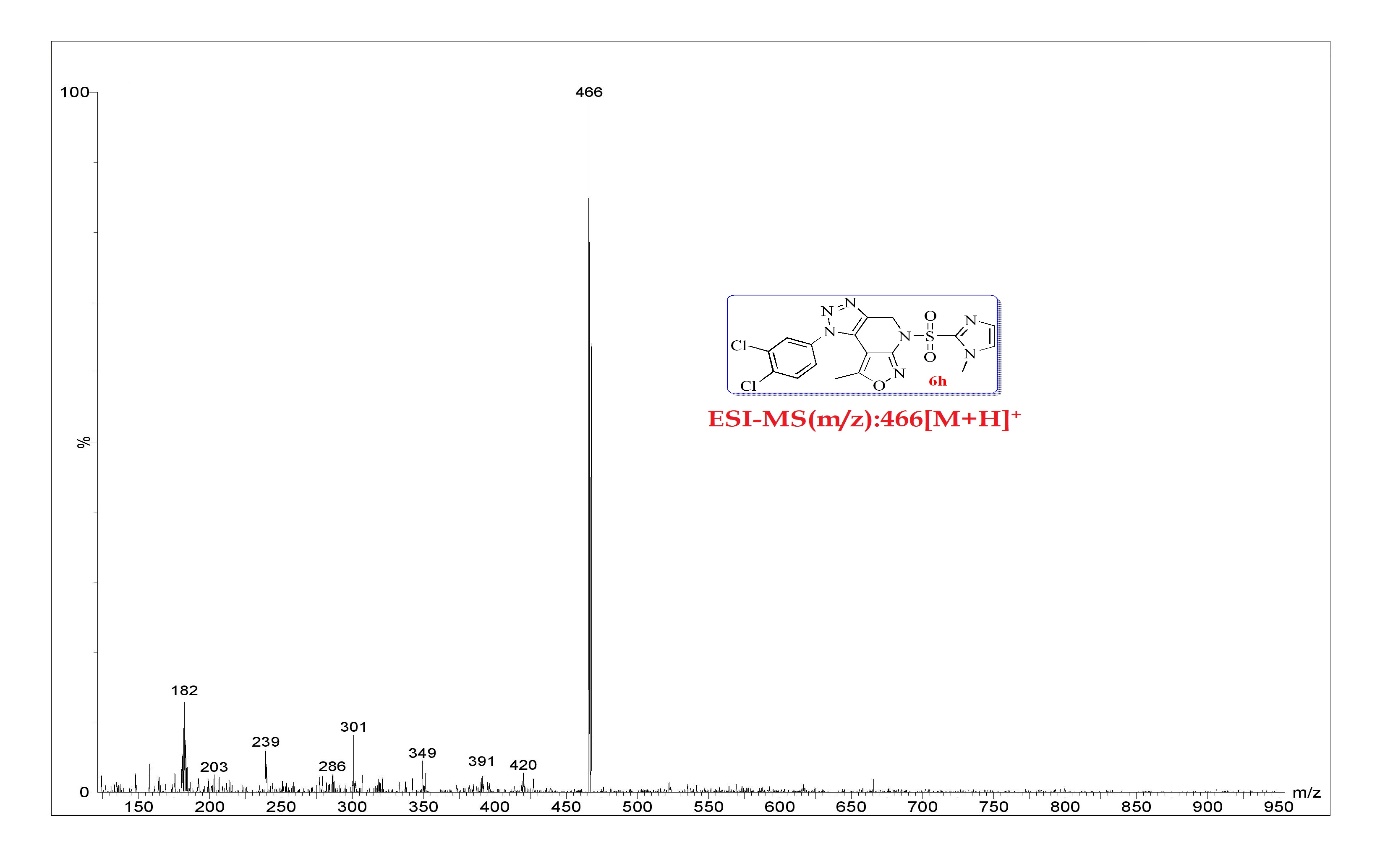

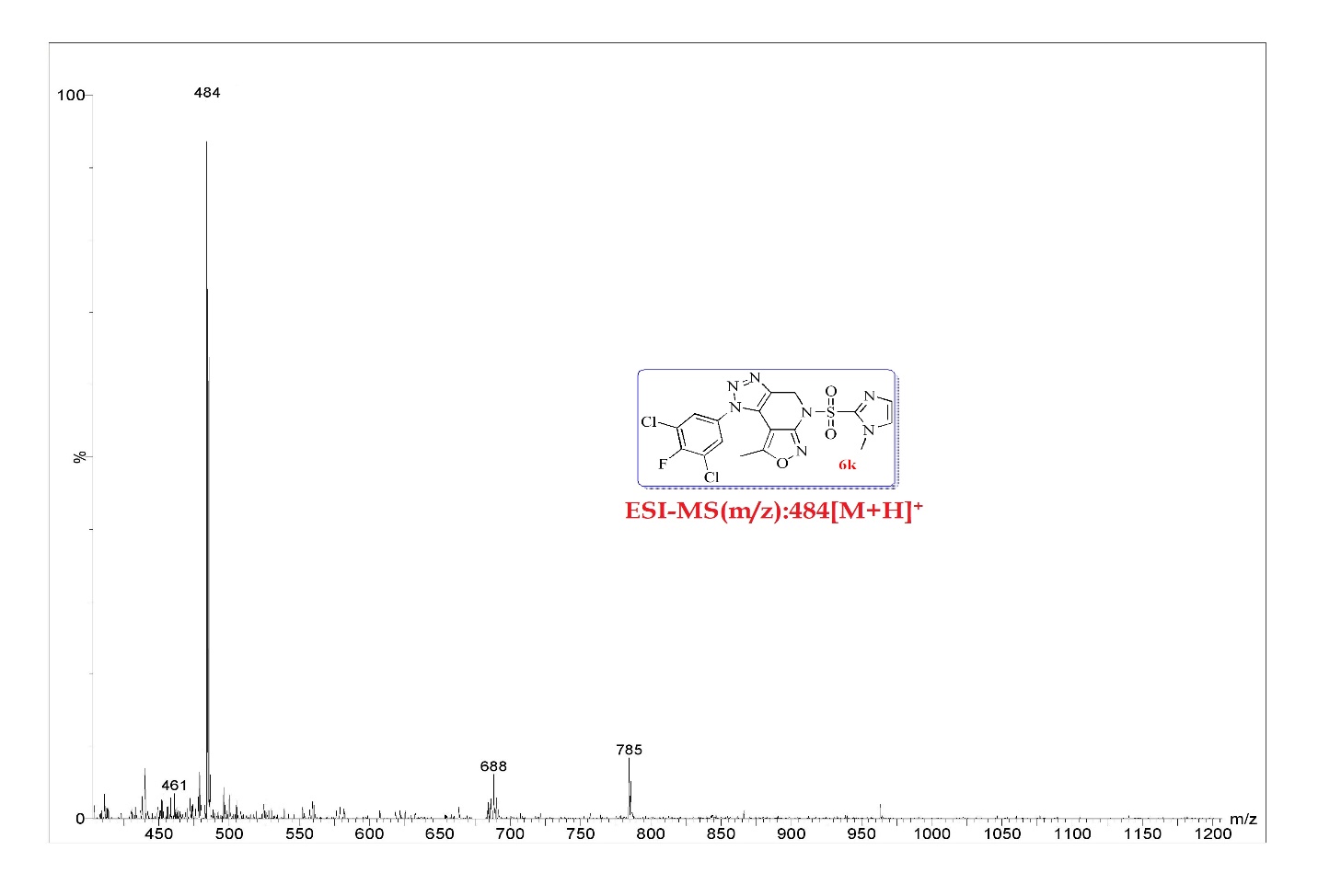

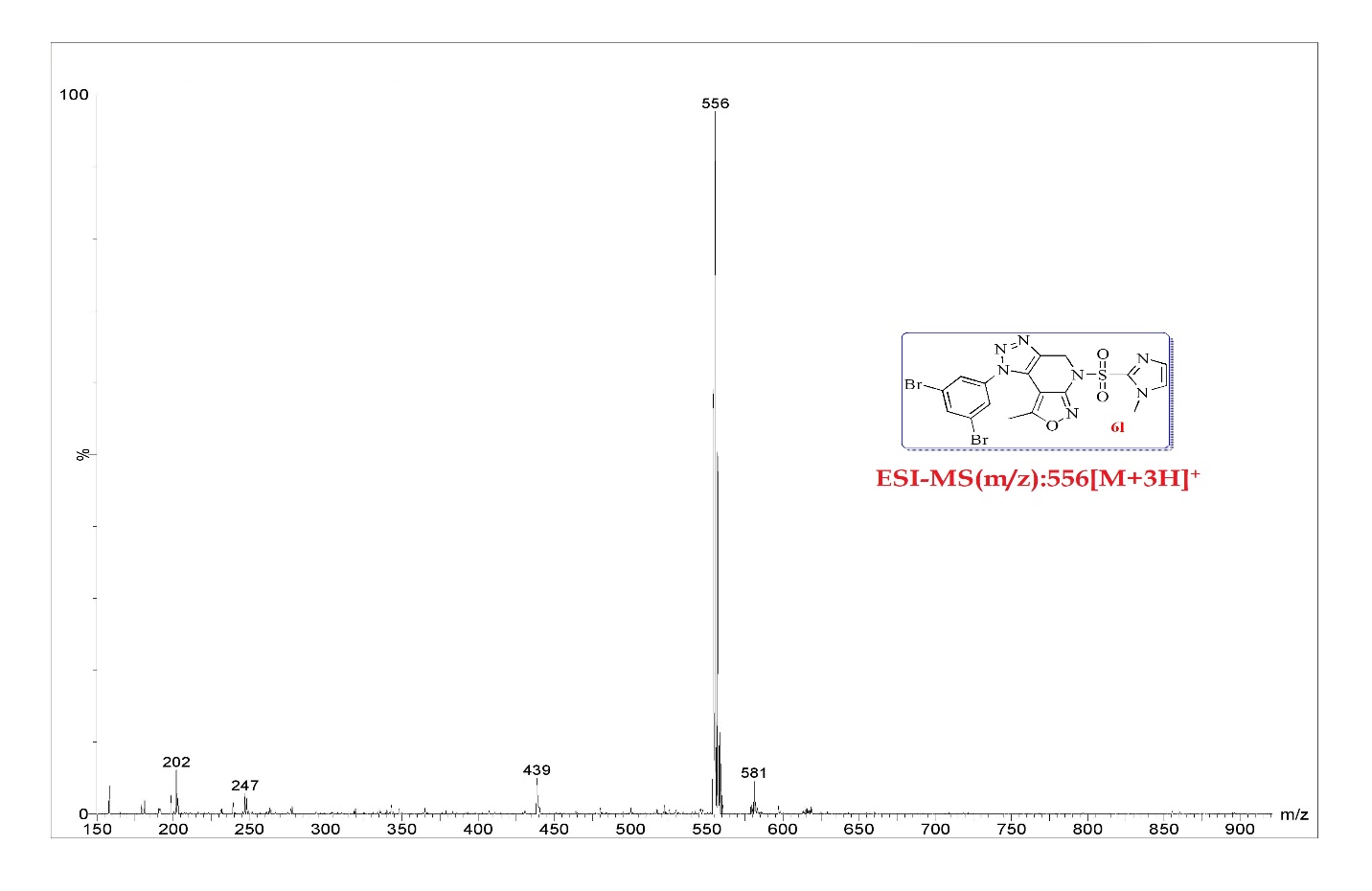

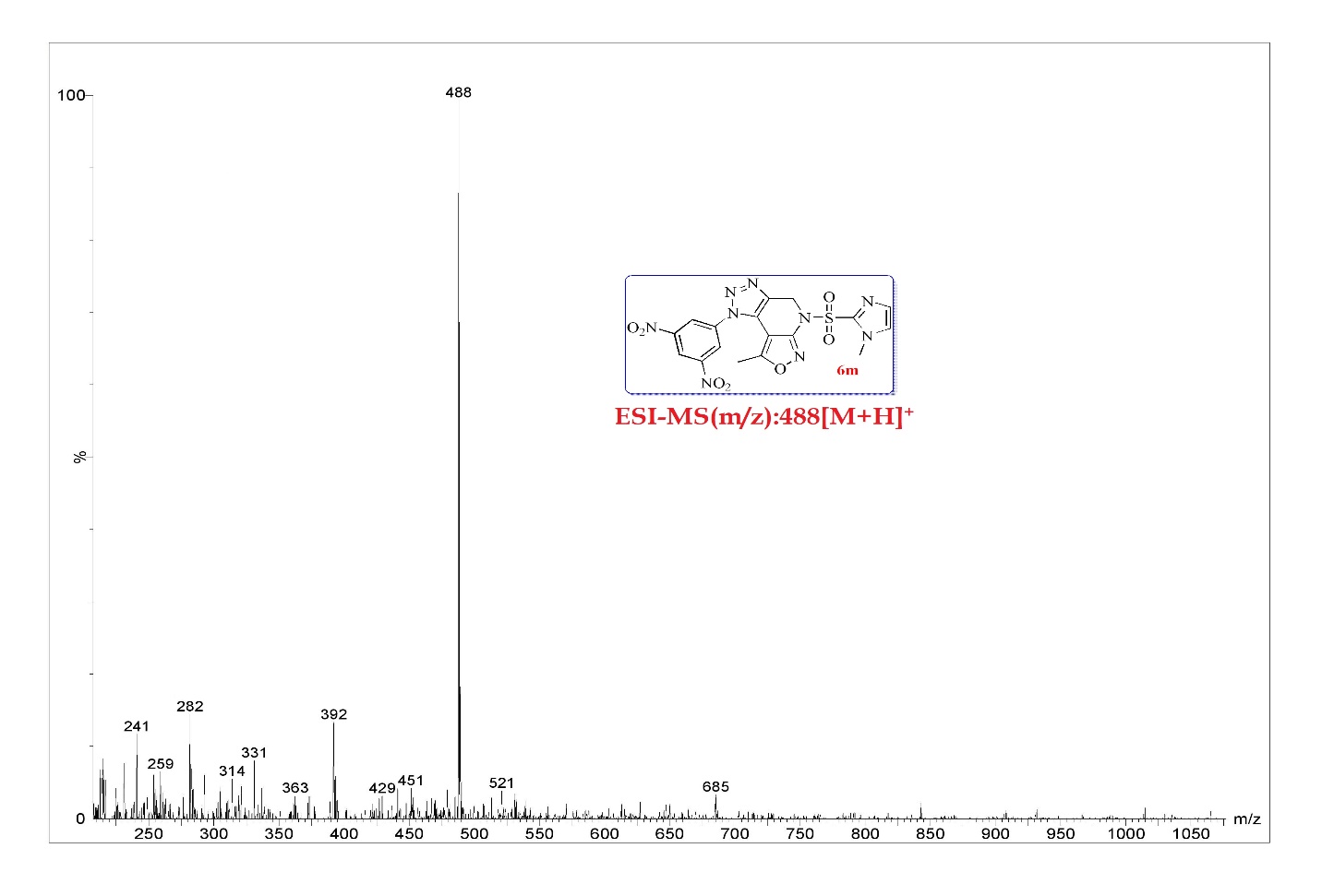

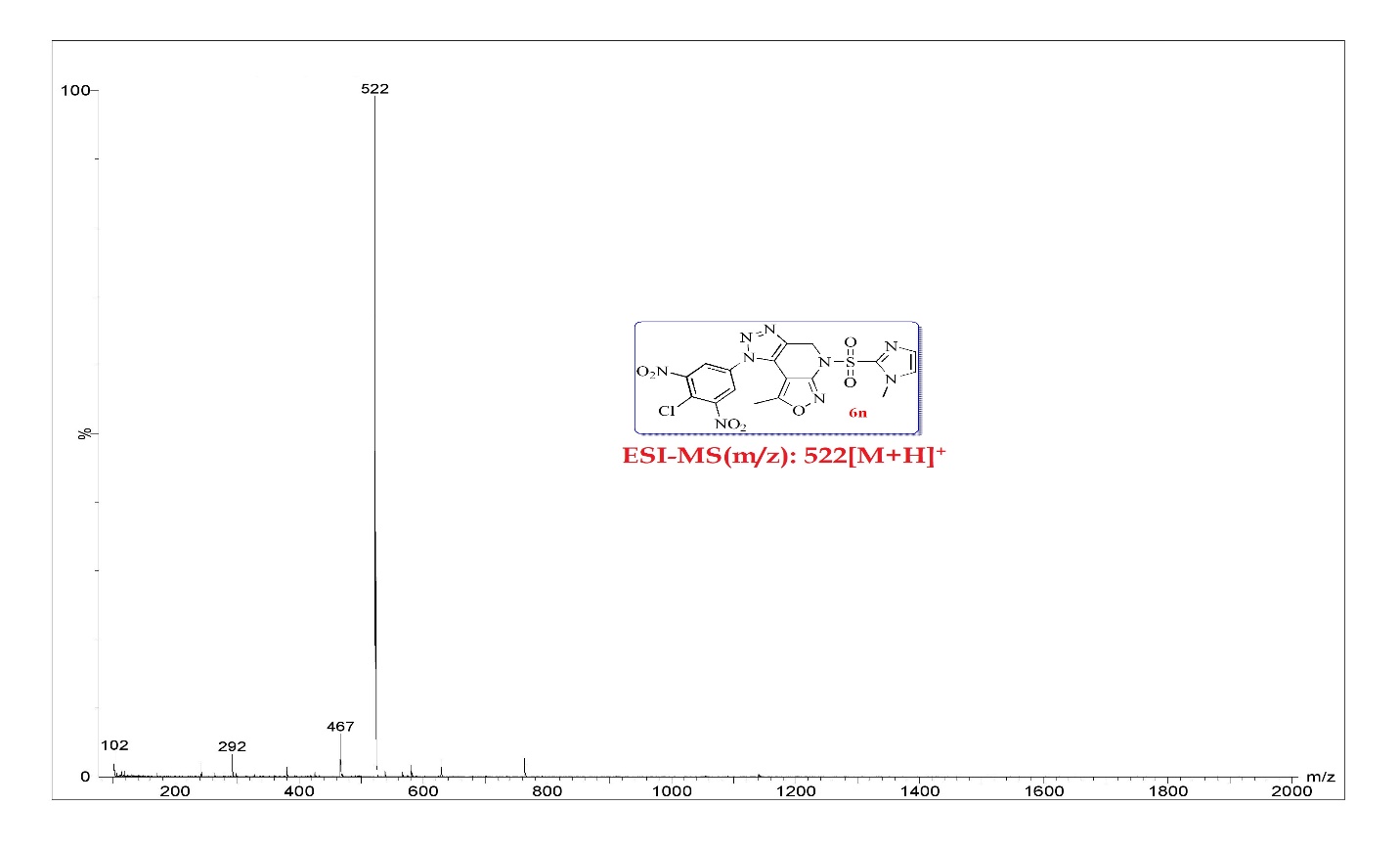

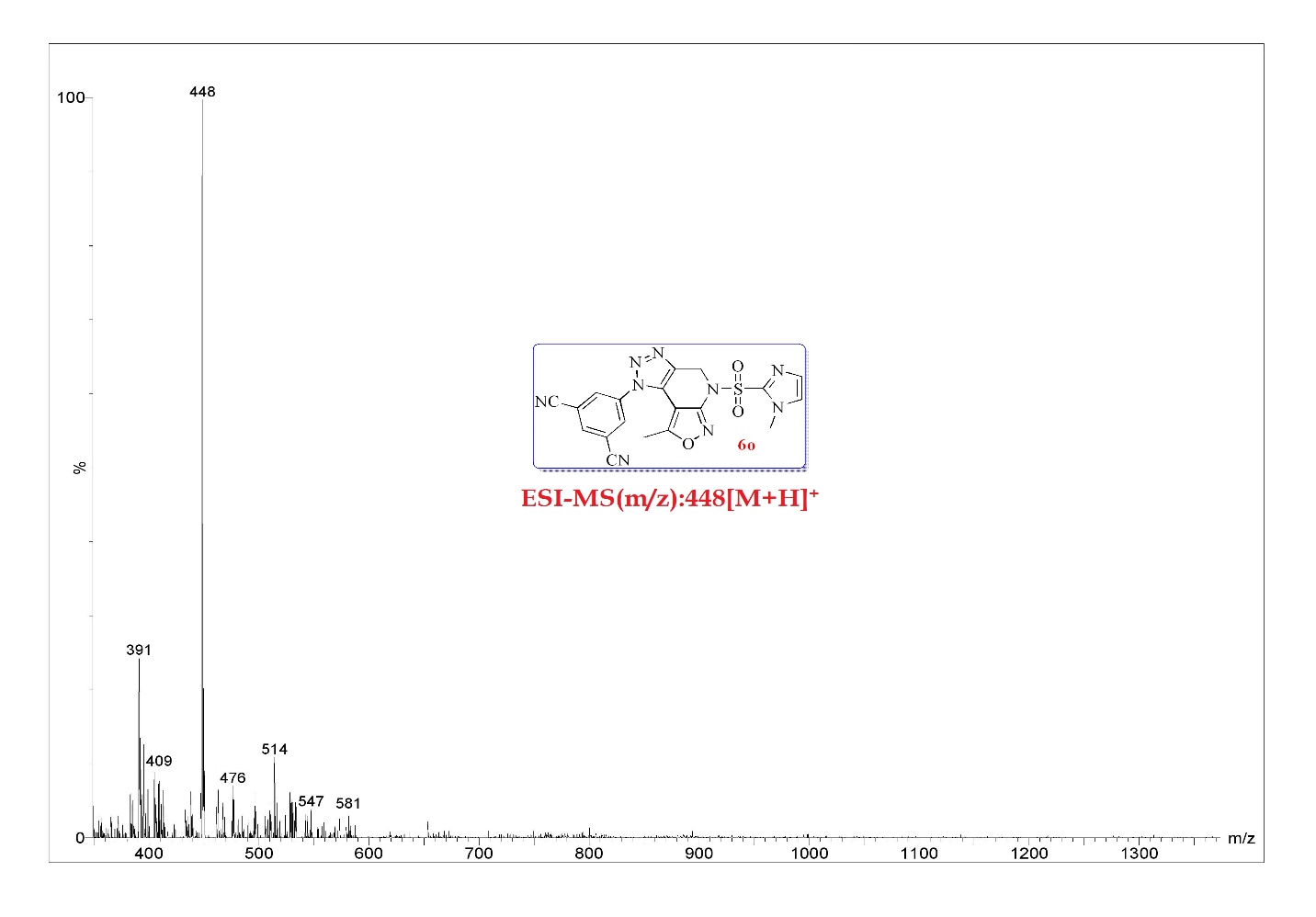

Supplement: Supplementary file 2 [file DataSheet1.docx]
